# New insights into the performance of human whole-exome capture platforms

**DOI:** 10.1093/nar/gkv216

**Published:** 2015-03-27

**Authors:** Janine Meienberg, Katja Zerjavic, Irene Keller, Michal Okoniewski, Andrea Patrignani, Katja Ludin, Zhenyu Xu, Beat Steinmann, Thierry Carrel, Benno Röthlisberger, Ralph Schlapbach, Rémy Bruggmann, Gabor Matyas

**Affiliations:** 1 Center for Cardiovascular Genetics and Gene Diagnostics, Foundation for People with Rare Diseases, Schlieren-Zurich CH-8952, Switzerland; 2 Department of Clinical Research, University of Berne, Berne CH-3010, Switzerland; 3 Functional Genomics Center Zurich, Zurich CH-8057, Switzerland; 4 Division of Scientific IT Services, ETH Zurich, Zurich CH-8092, Switzerland; 5 Division of Medical Genetics, Center for Laboratory Medicine, Aarau CH-5001, Switzerland; 6 Sophia Genetics SA, Lausanne CH-1015, Switzerland; 7 Division of Metabolism, University Children's Hospital, Zurich CH-8032, Switzerland; 8 Department of Cardiovascular Surgery, University Hospital, Berne CH-3010, Switzerland; 9 Interfaculty Bioinformatics Unit and Swiss Institute of Bioinformatics, University of Berne, Berne CH-3012, Switzerland; 10 Zurich Center for Integrative Human Physiology, University of Zurich, Zurich CH-8057, Switzerland

## Abstract

Whole exome sequencing (WES) is increasingly used in research and diagnostics. WES users expect coverage of the entire coding region of known genes as well as sufficient read depth for the covered regions. It is, however, unknown which recent WES platform is most suitable to meet these expectations. We present insights into the performance of the most recent standard exome enrichment platforms from Agilent, NimbleGen and Illumina applied to six different DNA samples by two sequencing vendors per platform. Our results suggest that both Agilent and NimbleGen overall perform better than Illumina and that the high enrichment performance of Agilent is stable among samples and between vendors, whereas NimbleGen is only able to achieve vendor- and sample-specific best exome coverage. Moreover, the recent Agilent platform overall captures more coding exons with sufficient read depth than NimbleGen and Illumina. Due to considerable gaps in effective exome coverage, however, the three platforms cannot capture all known coding exons alone or in combination, requiring improvement. Our data emphasize the importance of evaluation of updated platform versions and suggest that enrichment-free whole genome sequencing can overcome the limitations of WES in sufficiently covering coding exons, especially GC-rich regions, and in characterizing structural variants.

## INTRODUCTION

As a widely used method in genomic research and gene diagnostics, whole exome sequencing (WES) has the potential both to capture the entire coding region of all known genes including flanking intronic regions and to provide sequence data from these enriched genomic regions with sufficient read depth using a high-throughput DNA sequencing technology (1–6). Without enrichment, whole genome sequencing (WGS) is more comprehensive and allows for the characterization of the entire genome (7–10). As the human exome represents only ∼2% of the human genome, but harbours ∼85% of all known disease-causing mutations, WES is an increasingly used alternative to WGS (11–16).

Since performance comparisons of three major commercial exome enrichment platforms from Agilent, NimbleGen and Illumina were reported (2–6), new versions of these platforms have been introduced. To date, however, it has not been shown which new platform is superior in performance and most suitable for diagnostic purposes. For this reason and because our preliminary study (Supplementary Figures S1 and S2) revealed that an updated version does not necessarily lead to performance improvements, we assessed the most recent version (v) of each platform (updated end 2013).

Here, from the perspective of WES users, we present a performance evaluation of exome captures from Agilent (SureSelect v5+UTR), NimbleGen (SeqCap v3+UTR) and Illumina (Nextera Expanded Exome) applied to six human DNA samples extracted from blood, saliva or cultured cells and sequenced by different providers (vendors). One vendor (V1) used all three platforms and applied the same data analysis workflow (e.g. mapping, variant calling), ensuring best comparability. In order to avoid vendor bias and to assess the reproducibility of capture performance, each platform was used by an additional vendor specialized in the respective platform (V2, V3 and V4) as well, resulting in two sets of WES data per platform for all six samples. This study design allowed us to evaluate and compare the platforms not only within the same experimental and bioinformatics setting, similar to studies of previous platform versions (2–6), but also between different settings and among different DNA sources, revealing hitherto unreported performance differences, drawbacks and capabilities. Regarding coverage of the entire coding exome, for two samples we extended the performance evaluation of WES into the area of WGS as well, including data of the most recent HiSeq X Ten system.

## MATERIALS AND METHODS

### Preliminary study

Exomes of eight DNA samples (including all six samples of this study, Table 1) were enriched using previous capture platforms from Agilent (SureSelect Human All Exon kit v4+UTR) and NimbleGen (SeqCap EZ Human Exome v3) according to the manufacturer's instructions. Subsequently, prepared libraries were sequenced with 2 × 100 bp paired-end reads on a HiSeq 2000 sequencer (Illumina) at the Functional Genomics Center Zurich, including data analysis, which revealed distinct differences in the performance of these previous enrichment platforms (Supplementary Figures S1 and S2).

**Table 1.** Experimental design and characteristics of DNA samples used in this study

Sample #	Gender	DNA source	Extraction method	Purified	WES/Vendor^a^	WGS/Vendor (coverage)
44	female	blood	Qiagen column	no		no WGS
280	female	blood	Chemagen	no		no WGS
326	female	fibroblasts	Chemagen	no	Agilent/V1, V2	no WGS
2905	male	blood	Chemagen	yes	NimbleGen/V1, V3	no WGS
7344	female	blood	Chemagen	yes	Illumina/V1, V4	HiSeq/V3 (30×)
7739	female	saliva	Chemagen	yes		HiSeq/V1 (60×), V3 (30×), V4 (30×) and XTen/V4 (60×)

Qiagen column, DNA extraction using Qiagen QIAamp DNA Mini Kit; Chemagen, DNA extraction using PerkinElmer Chemagic Magnetic Separation Module I; Purified, purification of the extracted DNA by re-extraction using Qiagen QIAamp DNA Mini Kit; WES Agilent, SureSelect Human All Exon kit v5+UTR; WES NimbleGen, SeqCap EZ Exome (v3) +UTR; WES Illumina, Nextera Rapid Capture Expanded Exome; V1–V4, vendors 1–4; WGS HiSeq, TruSeq DNA PCR-Free Sample Preparation Kit on a HiSeq2000/2500 system; WGS XTen, TruSeq Nano DNA Sample Preparation Kit on a HiSeq X Ten system.

^a^For all six samples.

### Samples

DNA samples of six unrelated individuals, from which informed consent was obtained, were selected for this study. In each of these samples, Sanger sequencing and, if available, multiplex ligation-dependent probe amplification (MLPA) was previously performed for at least a subset of genes listed in Supplementary Table S1 (17–20).

Genomic DNA was extracted from EDTA-anticoagulated peripheral whole blood samples, saliva and cells cultured from aortic walls or skin biopsies (fibroblasts) using either QIAamp DNA Mini kit (Qiagen) or Chemagic Magnetic Separation Module I (Chemagen, Perkin Elmer) according to the manufacturer's instructions. Subsequently, some of the Chemagic extracted DNA samples were purified using QIAamp DNA Mini kit as well (Table 1). DNAs were quantified by OD measurements (NanoDrop 2000, Thermo Scientific) and used for exome enrichment according to the platform's standard requirements, i.e. 3 μg for Agilent, 1 μg for NimbleGen and 50 ng for Illumina.

### Exome enrichment and high-throughput sequencing

Exome enrichment was performed using the most recent versions (available by the end of 2013) of commercial sequence capture kits from Agilent (SureSelect Human All Exon kit v5+UTR), NimbleGen (SeqCap EZ Exome (v3) +UTR) and Illumina (Nextera Rapid Capture Expanded Exome). The captured libraries were sequenced using a HiSeq 2000/2500 sequencer (Illumina) and 2 × 100 bp paired-end sequencing according to the manufacturers’ recommendations. As the size of the designed target regions differs among platforms (Supplementary Table S2), the total expected read number was adjusted by the vendors to obtain 100× coverage in all cases (i.e. sequencing at 100× was requested for each sample). In addition to or instead of this comparable coverage for each platform, the usage of comparable number of reads was not pursued as this would favour the platform with the smallest exome design (Illumina).

Library preparation, sequence capture and high-throughput sequencing was performed by four different sequencing service providers (vendors V1–V4) according to their standard workflow. One vendor (V1) sequenced all six genomic DNA samples using all three exome enrichment platforms, whereas the three other vendors sequenced the six DNAs using only one platform in which they were specialized (V2: Agilent; V3: NimbleGen; V4: Illumina; Table 1). To extend our evaluation of the effect of DNA sources, 24 additional DNA samples extracted from blood (18 samples) or saliva (6 samples) were sequenced by V2 at 60× using Agilent capture (SureSelect v5) (Supplementary Figure S3).

In addition to the three updated standard exome enrichment platforms, the targeted (focused) enrichment of ∼7600 clinically relevant genes was exemplified for sample 7739 and two additional DNA samples using the Accuracy and Content Enhanced (ACE) clinical exome platform of Personalis with ∼4 μg DNA sequenced at 60× on a HiSeq2500 system (ACEv2, www.personalis.com). Moreover, for two out of the six DNA samples (7344 and 7739) WGS was also performed by three vendors (V1, V3 and V4) using Illumina's TruSeq DNA PCR-Free Sample Preparation Kit on a HiSeq 2000/2500 sequencer (Table 1). V1 performed WGS at 60× coverage using 4 μg DNA (sample 7739), whereas V3 (samples 7344 and 7739) and V4 (sample 7739) carried out sequencing at 30× using 2 and 4 μg DNA, respectively. Furthermore, for the DNA sample 7739 WGS was also performed at 60× on a most recent HiSeq X Ten sequencer (Illumina) by V4 using Illumina's TruSeq Nano DNA Sample Preparation Kit, which was not polymerase chain reaction (PCR)-free but at the time of library preparation the only kit compatible with the HiSeq X Ten system, according to the manufacturer's instructions for 350-bp insert size.

### Data analysis

Raw data processing, sequence read alignment from FASTQ to BAM format and variant calling to generate VCF files were performed by the four vendors (Supplementary Table S3). For our downstream analyses, aligned BAM files with removed duplicated reads were used, which were either directly provided by vendors (V3 and V4) or deduplicated by us (V1 and V2) using Picard tools version 1.108 or 1.118 (http://picard.sourceforge.net). To determine the number of reads and the coverage at defined minimal read depth (1, 10, 15 and 20×) of platform-specific target regions, RefSeq exons and the subset of exons analysed by Sanger sequencing and the ACE platform of Personalis, we used the SeqMonk program version v0.25.0/v0.26.0/v0.27.0 (http://www.bioinformatics.babraham.ac.uk/projects/seqmonk) with deduplicated BAM files and the settings ‘single-end reads’ for data import to enable the counting of individual reads and ‘remove exact duplicates’ for the option ‘feature probe generator’ (Supplementary Tables S4–S15). Genomic positions of target regions were downloaded from the platforms’ websites, whereas genomic coordinates of RefSeq exons were obtained from the UCSC Genome Browser (http://genome.ucsc.edu/, version December 2013). The GC content of RefSeq exons was calculated using the Galaxy platform (http://usegalaxy.org). Data from the X and Y chromosomes were included in our data analyses. For arithmetic means, upper and lower confidence limits (95% confidence intervals) were calculated using critical values of paired *t*-test distribution (*P* = 0.05) and indicated where appropriate.

In order to ensure the comparability of the platforms’ mutant (non-reference) allele enrichment performance, we restricted our analyses of the provided VCF files (filtered, if available, Supplementary Table S3) to shared sequence variants targeted by the design of each platform and located in RefSeq coding exons completely covered at 20× by all six platform-vendor combinations. The restriction to such shared sequence variants should largely exclude possible false-positive allele calls. Moreover, in order to avoid the influence of the vendors’ different data analysis workflows, we generated genome VCF (gVCF) files, which store sequencing information for both variant and non-variant positions, by applying the same in-house bioinformatics pipeline to all six FASTQ files provided by V1–V4 for each DNA sample (Supplementary Table S3). These gVCF files were filtered to include only positions with ≥20 reads and >30 quality scores in all samples and a non-reference allele (relative allele proportion 10–90%) detected in at least one of the six platform-vendor combinations within the platform's target region and 50-bp flanking sequences.

From our Sanger sequencing data, a total of 78 different single nucleotide variants (SNVs) and 11 different indels are known to be heterozygous in at least one of the six DNA samples, including clinically relevant, disease-causing mutations (Supplementary Tables S1 and S16). Thirty-five variants (30 SNVs and 5 indels) are located within our region of interest (ROI) for clinical sequencing, which consists of the entire protein-coding exonic region with −50 and +20-bp flanking intronic sequences (Supplementary Figure S4) and five SNVs affect UTR (Supplementary Table S1). Using deduplicated BAM and unfiltered VCF files (Supplementary Table S3), these variant positions were analysed for read depth, calling and fraction of non-reference (alternative, mutant) allele and GC content of 30-bp flanking sequences (Supplementary Figures S5–S11 and Supplementary Tables S17–S20).

In addition, three samples (44, 7344 and 7739) were run on a NimbleGen CGH/LOH 6 × 630K array (Roche) according to the manufacturer's instruction. This array contains probes for a total of 501 common SNVs within RefSeq coding exons. Array data of these SNVs located within the designed target region of each platform and exons completely covered at 20× by all six platform-vendor combinations (93, 101 and 53 SNVs for samples 44, 7344 and 7739, respectively) were compared to the corresponding WES variant calls in the provided unfiltered and recalibrated VCF files (Supplementary Table S3). In this comparison, array positions with no array results or false-negative or false-positive calls in all three platforms were excluded (81, 93 and 39 array positions remained for the samples 44, 7344 and 7739, respectively).

The copy number variant (CNV) detection capability of the platforms was assessed by comparing the relative read depth of exons with known deletions (i.e. one copy) to the read depth of normal flanking exons (i.e. two copies) in affected samples and controls using Integrative Genomics Viewer (IGV, Broad Institute). In addition, we also assessed the detection of these deletions by applying the WES-specific CNV calling tools cnMOPS (21) and XHMM (22) for appropriate samples as well as the WGS-specific algorithm BreakDancer (23) for corresponding chromosomes in all four PCR-free WGS datasets. All three CNV detection tools were used with default settings according to the developers’ instructions. Moreover, we calculated normalized relative base counts for RefSeq exons on autosomes according to MLPA data analysis (19). In details, the base counts of 21 769 exons completely (100%) covered at 20× in all 36 provided WES BAM files (i.e. in all combinations of the three platforms, six DNA samples and two vendors) were used for normalization. Copy number calculations were performed for the samples 44, 280, 2905, 7344 and 7739 relative to the sample 326, thereby only considering exons that in sample 326 achieved a coverage of 20× for at least one base and a total base count of ≥1000 in order to reduce the misleading effect of incorrectly mapped reads. Using this calculation, in order to further evaluate the CNV detection properties of the three updated exome enrichment platforms, the relative base counts of 182 exons in 12 different genomic regions with copy numbers known from a NimbleGen CGH 2.1M/4.2M array (Roche, Supplementary Table S21) were assessed. In WGS, using the same 21 769 exons for normalization as in WES, the reproducibility of copy number calculation was assessed based on the base counts of sample 7739 and five additional DNA samples sequenced at 60× on a HiSeq X Ten system as performed for sample 7739.

## RESULTS

### Platform design

In contrast to the designs (target regions) of previous platform versions, NimbleGen now offers the largest target region including coding and untranslated regions (UTR), covering 96 Mb (64 Mb coding + 32 Mb UTR) compared to 75 Mb (50 Mb coding + 25 Mb UTR) of Agilent and 62 Mb (42 Mb coding + 20 Mb UTR) of Illumina. Moreover, NimbleGen promises to capture the largest portion of the entire RefSeq coding exome (98%) followed by Illumina with 95% and Agilent with <94% (Table 2, Supplementary Figure S12 and Supplementary Table S2). Almost each individual RefSeq exon is completely targeted by the designs of NimbleGen (98%) and Illumina (94%) but, notably, in nearly half of the cases (46%) only partially covered by the design of Agilent (Supplementary Table S2).

**Table 2.** Overview of studies evaluating exome enrichment platforms as well as summary of which of the platforms performed best for the assessed aspects

	This study	Clark *et al*. 2011 (3)	Asan *et al*. 2011 (2)	Parla *et al*. 2011 (4)	Sulonen *et al*. 2011 (5)	Chilamakuri *et al*. 2014 (6)
Enrichment platforms	Agilent v5+UTR, NimbleGen v3+UTR and Illumina Nextera Expanded Exome	Agilent v3, NimbleGen v2 and Illumina TruSeq Exome	Agilent v1, NimbleGen v1 (in-solution), 2.1M array	Agilent v1 and NimbleGen v1	Agilent v1, v3 and NimbleGen v1, v2	Agilent v4, NimbleGen v3, Illumina TruSeq Exome and Illumina Nextera Expanded Exome
Sequencing platform	Illumina HiSeq 2000/2500 paired-end 100-bp reads	Illumina HiSeq 2000 paired-end 100-bp reads	Illumina HiSeq 2000 paired-end 90-bp reads	Illumina GAIIx, paired-end 76-bp reads	Illumina GAIIx, paired-end 82-bp reads	Illumina HiSeq 2000 paired-end 100-bp reads
DNA samples	Six samples performed by different vendors, 24 samples performed by one vendor using Agilent	One sample	One sample	Six HapMap samples (two for both platforms and four only for NimbleGen)	One sample for all platforms, 25 samples for one platform	One sample with two technical replicates per platform
Region for sequence variant calling	Common designed target region in RefSeq coding exons 100% covered at 20× by all platform-vendor combinations	Genome-wide	Designed target region with 200-bp flanking sequences	CCDS	Genome-wide, designed target region (individual and common), and CCDS	Designed target region (individual and common), CCDS, RefSeq (coding and UTR) and Ensembl
Largest designed target region	NimbleGen	Illumina	Agilent	Agilent	Agilent v2	NimbleGen
Largest coding region (reference)	NimbleGen (RefSeq)	Agilent (RefSeq, Ensembl CDS)	Agilent (CCDS)	Agilent (CCDS)	Agilent v2 (CCDS)	Illumina (CCDS, RefSeq, Ensembl)
Best designed target enrichment efficiency	Agilent	NimbleGen	NimbleGen (array and in-solution)	NimbleGen	NimbleGen v2	Agilent
Lowest off-target enrichment	Agilent and NimbleGen	NimbleGen	NimbleGen (array and in-solution)	NimbleGen	NimbleGen v1	Agilent and NimbleGen^a^
Best GC-rich region enrichment	Agilent	Agilent	NimbleGen array	No data	NimbleGen v2	Illumina Nextera
Highest accuracy of SNV detection (benchmark)	Agilent (Sanger sequencing, MLPA and SNP array)	Agilent (SNP array)	No clear difference among platforms (SNP array and WGS)	Agilent (HapMap and 1000 Genome Project data)	NimbleGen v2 (SNP array)	No determination of accuracy by comparison to a benchmark (only calling of SNVs)

^a^Estimated from provided figures, as off-target reads were reported as relative proportion of filtered reads rather than total mapped reads; CCDS, Consensus Coding Sequences.

### Exome enrichment performance

**Figure 1.**
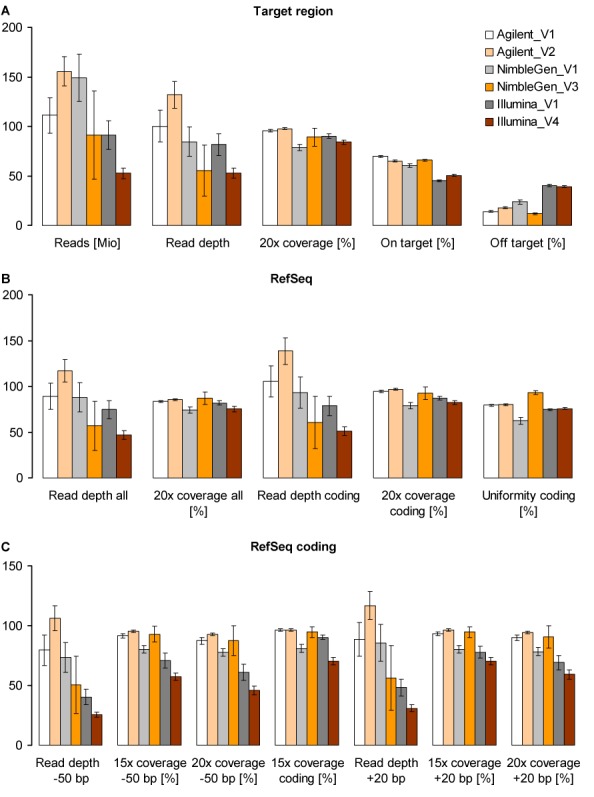
Enrichment efficiency of the three updated exome enrichment platforms (Agilent, NimbleGen and Illumina) performed by four vendors (V1, V2, V3 and V4). (**A**) Mean number of aligned reads (as million reads), mean read depth and percentage of coverage at 20× for each designed target region as well as mean percentage of on-target reads (i.e. within designed target regions) and mean percentage of off-target reads (i.e. within regions more than ±500 bp outside the designed target regions). Note that values for aligned reads indicate the total number of mapped reads without duplicates for V1 and V2 and only uniquely mapped reads without duplicates for V3 and V4 (Supplementary Table S3). (**B**) Mean read depth and percentage of coverage at 20× for all and only coding exons of the RefSeq database as well as uniformity of the coverage of RefSeq coding exons calculated as the fraction of exons reaching an average read depth within ±70% of mean read depth over all coding exons (uniformity coding). (**C**) Mean read depth and percentage of coverage at 15 and 20× for RefSeq coding exons as well as for −50-bp and +20-bp flanking intronic regions. Given are means of all six DNA samples (*n* = 6); error bars indicate 95% confidence intervals. Values were calculated using the SeqMonk program (http://www.bioinformatics.babraham.ac.uk/projects/seqmonk/) and are presented in Supplementary Tables S4-S8 and S12–S13. For complete coverage of RefSeq coding exons see Figure 3.

Platform designs may let users expect certain WES performances. However, the question is whether expectations derived from platform designs meet the real laboratory performances. In WES, captured DNA fragments sequenced with sufficient quality result in reads which can be aligned to the reference genome sequence (i.e. mapped), producing an appropriate alignment (BAM) file per DNA sample. In this study, for the six platform-vendor combinations, on average 55.2–90.4% of the raw reads remained after mapping and deduplication, with duplicates accounting for 8.4–37% of the mapped reads. Applying the same platform, the number of deduplicated mapped reads was significantly different between V1 and V2 (Agilent) as well as between V1 and V4 (Illumina). This difference is at least partially due to variation in the proportion of duplicated reads, suggesting the impact of laboratory workflow on WES (Figure 1A, Supplementary Figure S13 and Supplementary Tables S22–S24). Indeed, duplicated reads may rather depend on a laboratory workflow, whereas variation in the proportion of unaligned reads may rather be due to alignment tools as demonstrated by comparing provided and in-house generated BAM files (Supplementary Figure S13). Off-target enrichment was assessed as the fraction of total aligned reads which mapped more than 500 bp outside the designed target regions. As in 2011 (3), Illumina showed the highest proportion of off-target reads (∼40%) compared to Agilent and NimbleGen (Figure 1A and Supplementary Tables S4 and S5).

**Figure 2.**
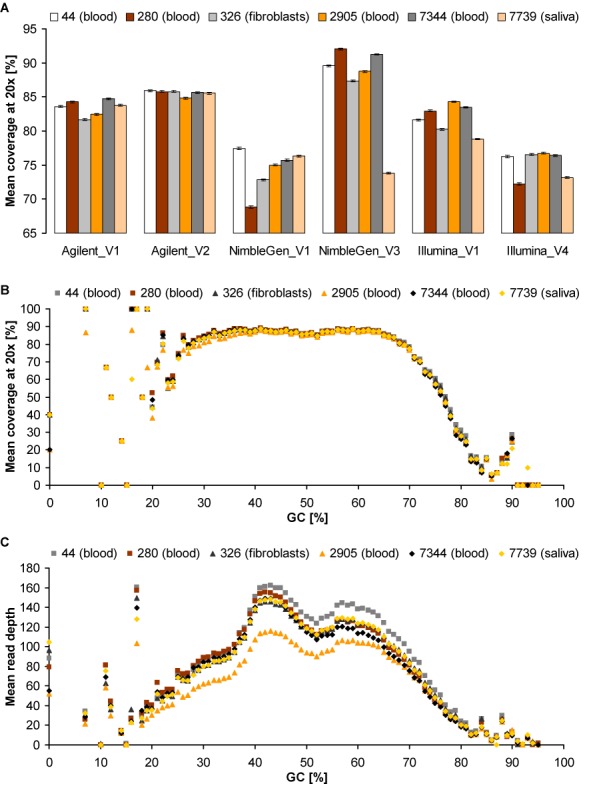
Differences among DNA samples. (**A**) Mean coverage of RefSeq exons (*n* = 233 644) at 20× (expressed in percentage of the entire exon length) for all six platform-vendor combinations and DNA samples (44, 280, 326, 2905, 7344 and 7739) derived from blood, fibroblasts or saliva. Values were obtained by using the SeqMonk program (www.bioinformatics.babraham.ac.uk/projects/seqmonk) and are presented in Supplementary Tables S6 and S12. Error bars indicate 95% confidence intervals for the arithmetic means of all corresponding exons. (**B** and **C**) Mean coverage at ≥20 reads (B) and mean read depth (C) of RefSeq exons per GC content for each DNA sample exemplified by the WES data of V2 using Agilent, demonstrating its high performance stability across samples.

While previous versions of NimbleGen showed the highest enrichment efficiency (2–5), the current version of NimbleGen was only able to achieve best exome coverage in some samples and vendor (Figures 1 and 2A). Similarly, also Illumina showed distinct inter-sample and inter-vendor variation. In contrast, for the Agilent platform the high enrichment efficiency of target and coding regions was stable between the two respective vendors and among all six DNA samples (Figures 1 and 2, Supplementary Figure S14). The latter result we confirmed by analysing 24 additional DNA samples extracted from blood or saliva (Supplementary Figure S3). This high enrichment performance and superior robustness of Agilent represents a clear improvement of previous versions (Table 2 and Supplementary Figures S1 and S2).

The comparison of the enrichment performances of the three platforms used with the same experimental setting by the same vendor (V1) revealed that Agilent reached the expected mean read depth of 100 (100×) for RefSeq coding region (105 ± 17), 94.7 ± 1.2% of which were sequenced at 20×, i.e. with a read depth of at least 20 (Figure 1B and Supplementary Table S6). NimbleGen with a mean read depth of 93 ± 17 showed the lowest 20× coverage of RefSeq coding region (79.0 ± 3.5%), whereas Illumina with lowest mean read depth (79 ± 11) overall achieved significantly higher 20× RefSeq coverage (87.0 ± 2.3%). Similar results for read depth and overall coverage performance was revealed by V1 also for the platforms’ target regions (Figure 1A and Supplementary Table S4). The discrepancy between read depth and coverage performance observed for NimbleGen and Illumina is most likely due to unequal read distribution, which can be driven by the GC content of genomic sequences (24).

The uniformity of the coverage of RefSeq coding exons was assessed by calculating the fraction of exons reaching an average read depth within ±70% of mean read depth over all coding exons (2). With the highest mean read depth, Agilent reached a fraction of ∼80% regardless of vendor. Similar results were obtained for Illumina (∼75%). In contrast, NimbleGen showed a high inter-vendor variation with ∼62% for V1 and ∼93% for V3 (Figure 1B and Supplementary Table S8), whereas WGS resulted in superior uniformity of the coverage of RefSeq coding exons with at least 97% (Supplementary Table S9).

**Figure 3.**
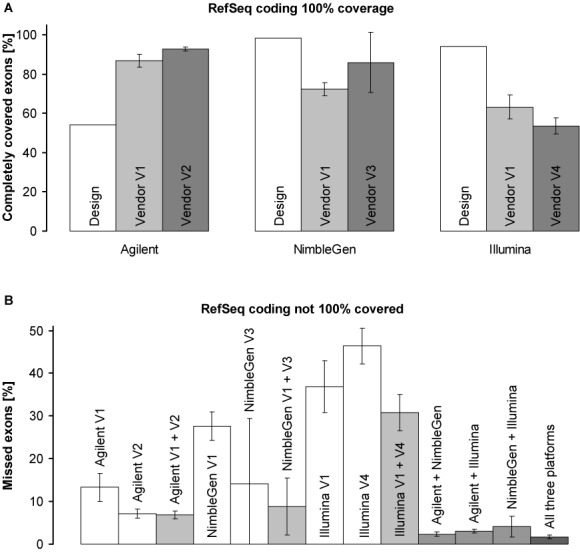
Complete (i.e. 100%) coverage of RefSeq coding exons. (**A**) Proportion of RefSeq coding exons 100% covered by each designed target region (design) and by ≥20 reads effectively produced by each vendor (vendors V1–V4). (**B**) Proportions of RefSeq coding exons not 100% covered at 20× (missed exons). If not otherwise indicated, data of all corresponding vendors are included. Given are means of all six DNA samples (*n* = 6); error bars indicate 95% confidence intervals. Values were calculated using the SeqMonk program (http://www.bioinformatics.babraham.ac.uk/projects/seqmonk/) and are presented in Supplementary Tables S2, S8 and S10.

In addition, we assessed what proportion of RefSeq coding exons are completely (i.e. 100%) covered at ≥20× and hence suitable for clinical WES (5). Regardless of vendor, Agilent performed best, however still far from 100%, with an average of 86.7 ± 3.3% (V1) and 92.8 ± 1.0% (V2) followed by NimbleGen with 72.4 ± 3.4% (V1) and 85.9 ± 14.1% (V3) as well as by Illumina with 63.1 ± 6.1% (V1) and 53.6 ± 4.2% (V4). Only 28.7 ± 5.8% of all RefSeq coding exons are completely covered at ≥20× by all three platforms (Figure 3A and Supplementary Tables S8 and S10). In fact, Agilent exceeded the expectation of complete (100%) exon coverage from the capture design (cf. only 54.2% of the RefSeq exons are covered 100% by the target design of Agilent), whereas NimbleGen (98.3% expected) and Illumina (94.0% expected) failed to reach their promised coverage (Figure 3A and Supplementary Table S2). Our data suggest that the proportion of incompletely covered exons can be reduced by combining platform, rather than vendor, performances. Indeed, the combination of the two best (Agilent and NimbleGen) and all three platforms left only 2.3 ± 0.5% and 1.7 ± 0.3% of the RefSeq coding exons uncovered at ≥20×, respectively, whereas the combined performance of both vendors using Agilent (V1 and V2) could only reduce the proportion of not completely covered exons from 7.2 ± 1.1% (Agilent alone by V2) to 6.8 ± 0.9% (Figure 3B and Supplementary Table S10). Alternatively, particularly focused enrichment can help to improve the standard WES coverage of exons of interest as exemplified using a clinical exome platform (Supplementary Table S11). However, in comparison to all exome enrichment platforms used in this study, WGS (60×) showed fewer uncovered exons at comparable read depth (i.e. at 10–15×; Supplementary Tables S9 and S11).

**Figure 4.**
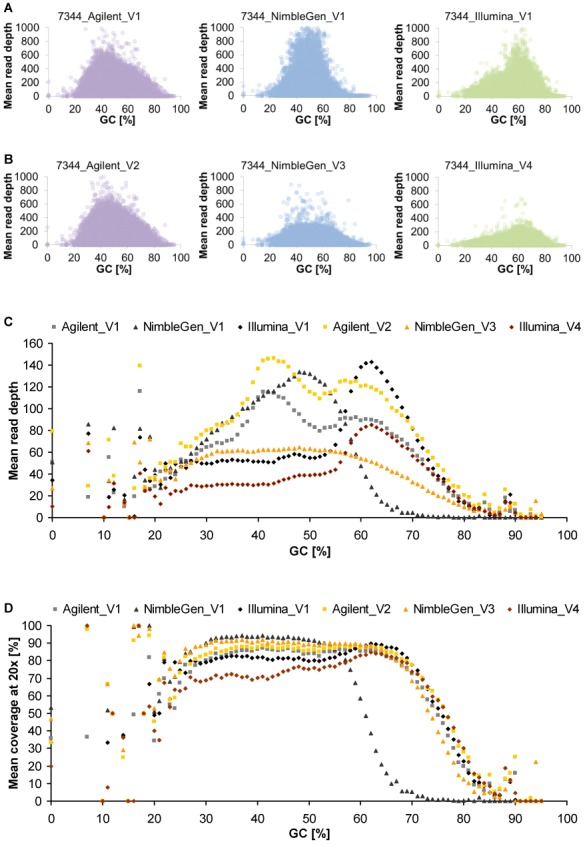
Differences in sensitivity to GC content among all platform-vendor combinations (average of all six DNA samples). (**A** and **B**) Scatter plot showing GC content and achieved read depth of RefSeq exons (coding and UTR) for the three updated exome enrichment platforms performed by the same vendor (V1, A) and different vendors (V2–V4, B), exemplified for sample 7344 (plots of all six samples are shown in Supplementary Figures S15 and S16). (**C**) Mean read depth of RefSeq exons per GC content shown as means of all samples. (**D**) Mean 20× coverage of RefSeq exons per GC content shown as means of all samples.

**Figure 5.**
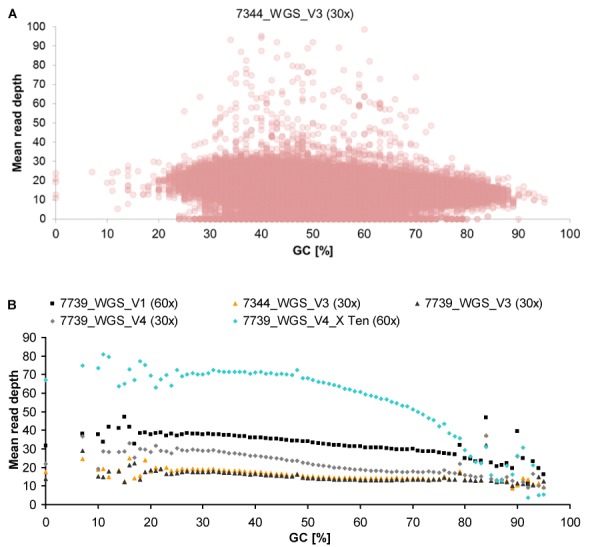
Influence of GC content on mean read depth in WGS. (**A**) GC content and achieved read depth of RefSeq exons (coding and UTR) exemplified by WGS of sample 7344 performed by V3 (plots of all WGS datasets are shown in Supplementary Figure S17). (**B**) Means of read depths of RefSeq exons per GC content. X Ten, HiSeq X Ten system.

To assess the impact of GC content on enrichment performance, we plotted mean read depth as well as mean coverage at 20× against the GC content of RefSeq exons (Figures 2B, C and 4 and Supplementary Figures S15 and S16). Both Agilent and Illumina suggested correlation between GC content and read depth regardless of vendor, whereas NimbleGen resulted in clear differences between vendors and when performed by V3, also among samples. Read depth of exons with very low (<20%) or high (>80%) GC content was variable or low for all platforms, however, Agilent thereby performed more robust and slightly better. This limitation of WES in capturing GC-rich regions also differently affected previous platform versions (Table 2) and can be overcome by WGS, as it is free from genomic hybridization/capture, especially by PCR-free WGS. Indeed, our PCR-free WGS data showed no distinct negative effect of high or low GC content on the mean read depth of RefSeq exons, covering GC-rich regions (>80%) much better than WES. Non-PCR-free WGS (HiSeq X Ten) achieved slightly lower enrichment of GC-rich exons but the observed bias was far less pronounced than in WES (Figure 5 and Supplementary Figure S17).

### Enrichment and detection of non-reference alleles

**Figure 6.**
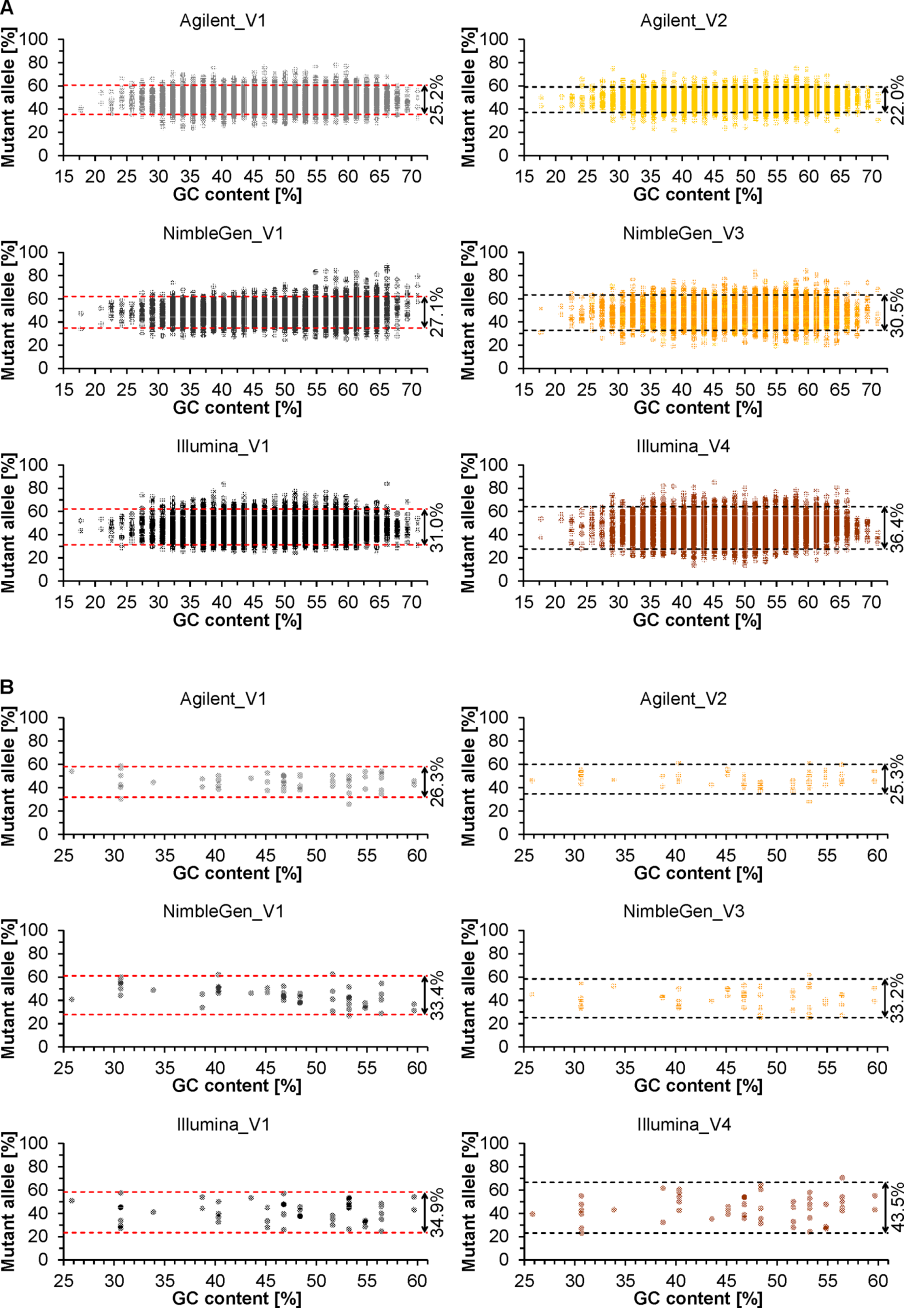
Relative proportions of non-reference (mutant) alleles called in the VCF files provided by vendors (V1–V4). The analysis was restricted to shared heterozygous variants within the designed target regions of the three platforms (Agilent, NimbleGen and Illumina) located in exons completely (100%) covered at 20× by all six platform-vendor combinations. (**A** and **B**) Heterozygous SNVs (A) and indels (B) listed according to GC content of 30-bp flanking sequences (for indel lengths see Supplementary Figure S18). Shown are values of all six DNA samples. Dashed lines indicate an interval within which 95% of the relative proportions of non-reference alleles lie (calculated according to the Student's *t* distribution as the mean of *n* percentage values ± critical *t*-value (*t*_crit,n-1_) × *SD* using *n* = 8 687, *t*_crit_ = 1.960 and *n* = 51, *t*_crit_ = 2.009 for A and B, respectively).

Sequence variant detection by WES requires both the experimental enrichment and bioinformatics calling of mutant alleles. As read alignment and variant calling can considerably influence sequence variant detection (http://genomeinabottle.org; http://www.bioplanet.com/gcat; 25), we focussed on data generated by the same bioinformatics workflow for the assessment of the enrichment of non-reference (mutant) alleles. Accordingly, we focused on filtered VCF files generated by the bioinformatics workflow of V1 for all three platforms as well as gVCF files created by our in-house data analysis pipeline for all six platform-vendor combinations. We restricted the analysis of the provided VCF files to RefSeq coding regions, whereas we created our gVCF files for the entire target region of each platform including 50-bp flanking sequences. In both cases, for each DNA sample only non-reference alleles covered and called by all six platform-vendor combinations were considered in order to reduce the possibility of miscalling. In the provided VCF files, by assessing the mean relative proportion of non-reference alleles (26), no considerable difference was observed among platforms neither for all variants nor for indels only, suggesting comparable sensitivity to the detection of mosaicism (Figure 6). Nevertheless, the enrichment of non-reference alleles was more stable for Agilent, resulting in reproducibly lower variation in the relative proportions of alternative alleles compared to NimbleGen and Illumina (Figure 6, Supplementary Figure S18 and Supplementary Table S25). Moreover, as one might expect considering hybridization mismatches, the enrichment of non-reference alleles was rather lower (<50%) than the capture of reference ones (>50%). All these results derived from the analysis of provided VCF files are supported by comparable findings obtained from heterozygous SNVs characterized by Sanger sequencing (Supplementary Figure S10) as well as from shared heterozygous SNVs and indels in our in-house generated gVCF files (Supplementary Figures S19 and S20 and Supplementary Table S26).

To determine the accuracy of WES variant detection, we analysed heterozygous SNVs and small indels, including clinically relevant mutations, previously characterized by Sanger sequencing in our DNA samples. Unlike previous studies (Table 2), we restricted this analysis to variants in a ROI relevant for clinical sequencing, which comprises exons with −50-bp and +20-bp flanking intronic sequences, as well as to UTR variants. This Sanger-based benchmarking provided not only the most accurate reference genotypes (25) but also allowed us to compare WES with Sanger sequencing with respect to clinical use in our set of genes. For all assessed heterozygous exonic positions, a minimum read depth of 20 was achieved by all three updated platforms. The coverage of intronic positions, however, varied among platforms and DNA sources similar to the performance observed genome-wide (Supplementary Figures S5 and S6). Regardless of vendor and hence also of mapping and variant calling workflow, Agilent correctly detected all our SNVs and called no false-positive variants, whereas NimbleGen (V1) failed to detect one SNV in UTR and Illumina (V4) identified three false-positive heterozygous SNVs (Supplementary Tables S17–S19). By assessing calling accuracy for indels, which was not analysed in previous studies (2–6), we found that all three platforms failed to detect some small indels, although Agilent identified the highest number of indels in ROI (Supplementary Table S20). All analysed SNVs covered by array data (81, 93 and 39 for samples 44, 7344 and 7739, respectively) were called correctly in WES regardless of platform and vendor (Supplementary Table S27).

Moreover, our gVCF files generated by using the same bioinformatics pipeline also allowed a comparative assessment of variant detection in all six platforms-vendor combinations. We thereby focussed on positions with ≥20 reads and >30 quality scores which were called as heterozygous by only one or by all but one platform and hence indicate possibly false-positive or false-negative variant calls. Agilent resulted in the lowest proportion of such putative calling errors (0.82 ± 0.04% of all >14 000 analysed variant positions) followed by NimbleGen (1.52 ± 0.10%) and Illumina (1.66 ± 0.19%) (Supplementary Figure S21 and Supplementary Table S28). However, high quality variants (≥20 reads and quality >30) called by all platforms may not be sufficient to exclude all putative alignment errors and other variant calling pipelines may also lead to different results. Indeed, WES users should be aware that for the same raw reads (FASTQ file) different read aligner and variant caller combinations may result in considerably different number of true and false variants (Genome Comparison & Analysis Testing at http://www.bioplanet.com/gcat). The assessment of the effect of bioinformatics pipelines on WES variant detection, however, is beyond the scope of this study and should be addressed by further works focusing on this topic.

The quantitative, CNV detection properties of the platforms (27,28) were not evaluated in previous studies (2–6). Thus, by using IGV we analysed one DNA sample (sample 44) that harbours a previously characterized heterozygous 27-kb deletion affecting the complete exon 1 of the *FBN1* gene (19). Compared to the other five samples captured by using Agilent and Illumina, the read depth of the deleted exon 1 was distinctly lower than the read depth of the flanking undeleted exons 2–5. For NimbleGen, however, the distribution of read depth among samples and exons was less stable (regardless of alignment tool as confirmed by our in-house generated BAM files) and thus the heterozygous one-exon deletion was not clearly detectable (Supplementary Figure S22). Moreover, by using IGV we analysed WES and WGS data for a 7-kb deletion to compare their potential to detect large deletions (Supplementary Figure S23). Both sequencing strategies allowed the detection of large deletions by a reduction of read depth compared to flanking exons and sequences, respectively. However, by using WGS a more accurate determination of breakpoint positions is possible, whereas by using WES additional methods may be needed (29). Using CNV calling tools, none of the two tested deletions were detected in the WES datasets by the WES-specific cnMOPS (21) and XHMM (22) methods. In contrast, the 7-kb deletion was called in all three PCR-free WGS data sets (V1, V3 and V4) using the algorithm BreakDancer (23) (data not shown).

At genome-wide level, we extended the evaluation of the CNV detection properties of the three updated exome enrichment platforms not only for a large number of exons and different DNA samples but also for more known CNVs. The comparison of the relative base counts of 21 769 RefSeq exons completely covered at 20× in all 36 platform-vendor-sample combinations showed the lowest variation for Agilent regardless of vendor and revealed comparable variation between WES and non-PCR-free WGS (Supplementary Figures S24 and S25 and Supplementary Table S29). The lowest variation of Agilent and thus its potential for best CNV detection was confirmed by assessing 182 exons with copy numbers known from array CGH (Supplementary Figure S26 and Supplementary Table S30).

## DISCUSSION

Users of WES expect coverage of the entire coding region of all known genes and sufficient read depth for the covered regions. This comparative study provides the most recent and comprehensive data to answer the question of which current standard WES enrichment platform is most suitable to meet these expectations. By including different sequencing providers (vendors) and samples into our performance comparison, our study design allowed us to evaluate the three most recent standard exome enrichment platforms not only within the same experimental and bioinformatics setting but also between different settings and among different DNA sources. Our study focuses on the enrichment of both reference and non-reference alleles, keeping the influence of bioinformatics workflow, which is a matter of ongoing research, as low as possible. We observed that the Agilent SureSelect Human All Exon v5+UTR enrichment platform is superior to the other two platforms with regard to overall performance and robustness. This superior performance of the most recent Agilent platform is, however, not applicable to its previous versions as shown by comparisons in 2011 (Table 2) and our preliminary study (Supplementary Figures S1 and S2).

Although the NimbleGen platform is designed to enrich the largest target region and the highest proportion of the coding region of the genome, the most recent version of the Agilent platform produces higher and more consistent exome coverage. This discrepancy may at least partially be explained by different exome designs. Whereas the designed target regions of NimbleGen and Illumina represent the region intended to be enriched, the target region specified by Agilent only includes sequences effectively covered by hybridization probes. Thus, the designed exome coverage does not completely reflect effective hybridization probe coverage and exome enrichment efficiency (Figure 3A, Supplementary Figures S4 and S12, and Supplementary Table S2). This illustrates that information on the designed target region provided by platform selling companies may be misleading for WES users, emphasizing the need for laboratory evaluation of real platform enrichment performances. From a technical point of view, the performance of Agilent may also, at least partially, be explained by the relatively long RNA baits of this platform. It appears that longer RNA baits lead to better hybridization and enrichment efficiency as well as tolerate larger hybridization mismatches and thus provide more stable post-capture representation of non-reference alleles harbouring sequence variants, especially indels (Figure 6, Supplementary Figures S10 and S18–S20, and Supplementary Table S20). However, we observed that the higher hybridization efficiency and mismatch tolerance of Agilent does not result in increased, unspecific off-target capture (Figure 1A).

Alternatively, NimbleGen may be the enrichment method of choice for users interested in regions exclusively covered by this platform. However, users should be aware of more pronounced bias for GC-rich regions and potentially reduced enrichment efficiency due to sensitivity to vendor and DNA sources (Figures 1, 2 and 4). This bias and sensitivity may be explained, at least partially if not all, by the relatively low hybridization temperature during exome enrichment (47°C compared to 65°C and 58°C in Agilent and Illumina, respectively) and/or by the relatively short size of hybridization probes (55–105 bp DNA compared to 90/120 bp RNA and 95 bp DNA in Agilent and Illumina, respectively; Supplementary Table S2). For applications with limited starting material, Illumina with 50 ng required DNA amount is superior to NimbleGen and Agilent, both of which require more DNA as standard input (Supplementary Table S2). For clinical WES, particularly focused exome enrichment or the combination of Agilent and NimbleGen or of all three platforms can result in 100% sequence coverage of more exons than by using the standard platforms alone. As the three platforms neither alone nor in combination can completely capture all coding exons, the enrichment performance of each platform requires improvement, at least for exons of clinically relevant genes (Figure 3B, Supplementary Figure S27 and Supplementary Tables S9–S11).

Like Sanger sequencing, WES allows the simultaneous analysis not only of coding exons but also of flanking intronic sequences involved in normal splicing as well as the sufficient enrichment of non-reference alleles harbouring SNVs and small indels, all of which are essential for the detection of disease-causing mutations. Thus, the question arises whether or not WES can replace Sanger sequencing in mutation detection (30). Our data suggest that with respect to RefSeq coding exons and flanking intronic sequences none of the three updated WES platforms is suitable to replace Sanger sequencing, although Agilent appears to be more suitable, not least due to its superior robustness (Figure 2). Hence, for best results, particularly in clinical WES, the complete representation and sufficient coverage of each tested gene region has to be ensured, especially for GC-rich regions and deeper intronic positions.

Indeed, WES may fail to capture such regions and have difficulties to detect CNVs. We observed that capture-free, especially PCR-free, WGS can overcome these limitations, making WGS superior to WES, at least in those cases. Furthermore, the exome is not a fixed entity and still subject to changes. Projects such as ENCODE (31) will enhance the interpretation of non-coding, regulatory variants and their importance in genomic research and gene diagnostics will increase. With such changes in knowledge, WES capture design will require constant adaptation and for unsolved cases a re-run of WES will have to be considered, whereas by using WGS the entire genomic information is largely present so that only data analysis has to be repeated. One may assume that WGS will become less expensive, as shown by the recent introduction of the HiSeq X Ten system, and thus be more popular in the near future. However, it remains to be answered whether WGS will complement or replace WES. It is probable that WES with less and better interpretable sequencing data and emerging better enrichment performance will be widely used as an effective alternative to WGS in both research and diagnostics. Nevertheless, the clinical application of these powerful tools should proceed with care and be supported by the patient's health insurance, especially in testing a large number of exons and genes as well as in cases in which no *a* *priori* knowledge of gene(s) responsible for a particular disease phenotype exists.

## SUPPLEMENTARY DATA

Supplementary Data are available at NAR Online.

SUPPLEMENTARY DATA
