# Supplementary material for: New insights into the performance of human whole-exome capture platforms
Source: Nucleic Acids Res. 2015 Mar 27;43(11):e76. doi: 10.1093/nar/gkv216 (PMC4477645; doi:10.1093/nar/gkv216)
Supplement: SUPPLEMENTARY DATA [file supp_43_11_e76__index.html]

New insights into the performance of human whole-exome capture platforms — SUPPLEMENTARY DATA 

# New insights into the performance of human whole-exome capture platforms

## SUPPLEMENTARY DATA

**Files in this Data Supplement:**

- SUPPLEMENTARY DATA
