# Supplementary material for: New insights into the performance of human whole-exome capture platforms
Source: Nucleic Acids Res. 2015 Mar 27;43(11):e76. doi: 10.1093/nar/gkv216 (PMC4477645; doi:10.1093/nar/gkv216)
Supplement: SUPPLEMENTARY DATA [file supp_gkv216_nar-03332-met-g-2014-File002.pdf]

Nucleic Acids Research - Methods

SUPPLEMENTARY INFORMATION

New insights into the performance of human whole-exome capture platforms

Janine Meienberg[1,#], Katja Zerjavic[1,#], Irene Keller[2], Michal Okoniewski[3,4], Andrea Patrignani[3], Katja Ludin[5], Zhenyu Xu[6], Beat Steinmann[7], Thierry Carrel[8], Benno Röthlisberger[5], Ralph Schlapbach[3], Rémy Bruggmann[9], and Gabor Matyas[1,8,10,*]

[1] Center for Cardiovascular Genetics and Gene Diagnostics, Foundation for People with Rare Diseases, Schlieren-Zurich, CH-8952, Switzerland
[2] Department of Clinical Research, University of Berne, Berne, CH-3010, Switzerland
[3] Functional Genomics Center Zurich, Zurich, CH-8057, Switzerland
[4] Division of Scientific IT Services, ETH Zurich, Zurich, CH-8092, Switzerland
[5] Division of Medical Genetics, Center for Laboratory Medicine, Aarau, CH-5001, Switzerland
[6] Sophia Genetics SA, Lausanne, CH-1015, Switzerland
[7] Division of Metabolism, University Children's Hospital, Zurich, CH-8032, Switzerland
[8] Department of Cardiovascular Surgery, University Hospital, Berne, CH-3010, Switzerland
[9] Interfaculty Bioinformatics Unit and Swiss Institute of Bioinformatics, University of Berne, Berne, CH-3012, Switzerland
[10] Zurich Center of Integrative Human Physiology, University of Zurich, Zurich, CH-8057, Switzerland

* To whom correspondence should be addressed. Tel: +41 43 433 86 86; Fax: +41 43 433 86 85; Email: matyas@genetikzentrum.ch

[#] The authors wish it to be known that, in their opinion, the first 2 authors should be regarded as joint First Authors.

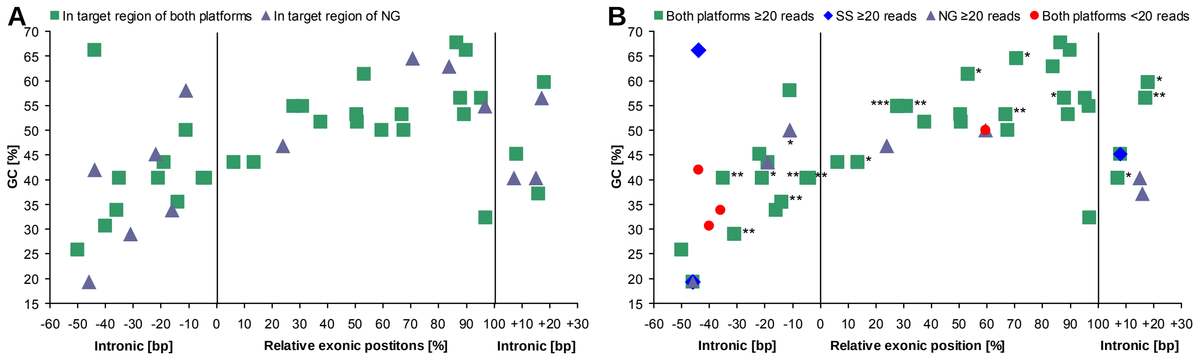

Figure S1. Preliminary study: Positions of selected heterozygous sequence variants detected by Sanger sequencing in our region of interest (exons with -50-bp and +20-bp flanking intronic sequences) as a function of the GC content of 30-bp flanking sequences. (**A**) Occurrence of variants in the designed target region of each platform. (**B**) Platforms providing ≥20 reads at heterozygous variant positions. Note that, contrary to expectations, not all exonic positions are within the designed target region of the Agilent platform. Furthermore, none of the two platforms achieved complete coverage of all exonic positions at ≥20 reads. Exonic positions are given in percentage [%] relative to the length of the corresponding exon, whereas intronic positions are given as absolute positions in base pairs [bp]. SS, Agilent SureSelect v4+UTR; NG, NimbleGen SeqCap v3; *, heterozygous in one additional DNA sample with the same symbol; **, heterozygous in two additional DNA samples with the same symbol; ***, heterozygous in three additional DNA samples with the same symbol.

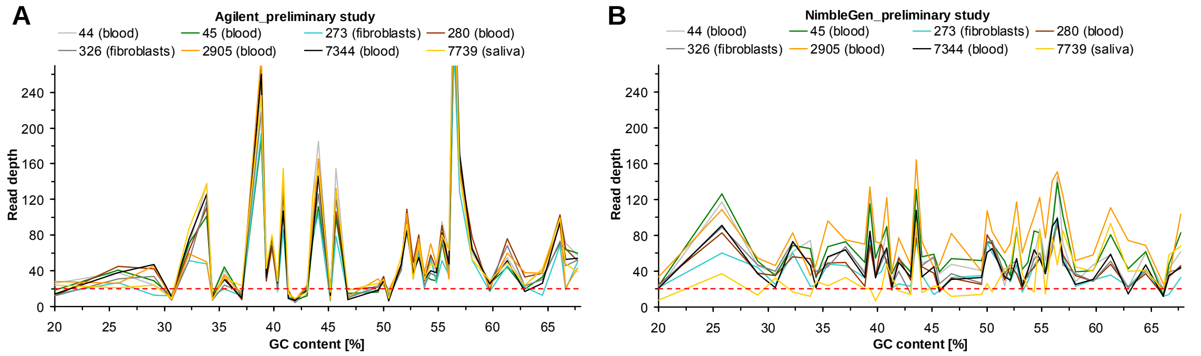

Figure S2. Preliminary study: Read depth for 44 different SNV positions within our region of interest (coding exons and -50-bp and +20-bp flanking intronic sequences) called as heterozygous in at least one of the eight samples (the six samples from this study as well as 45 and 273) plotted against the GC content of 30-bp flanking sequences. (**A**) Agilent (SureSelect Human All Exon kit v4+UTR). (**B**) NimbleGen (SeqCap EZ Exome v3). Red dashed line indicates our limit of 20 reads. Note the high number of positions failed to reach the limit of 20 reads by the Agilent platform.

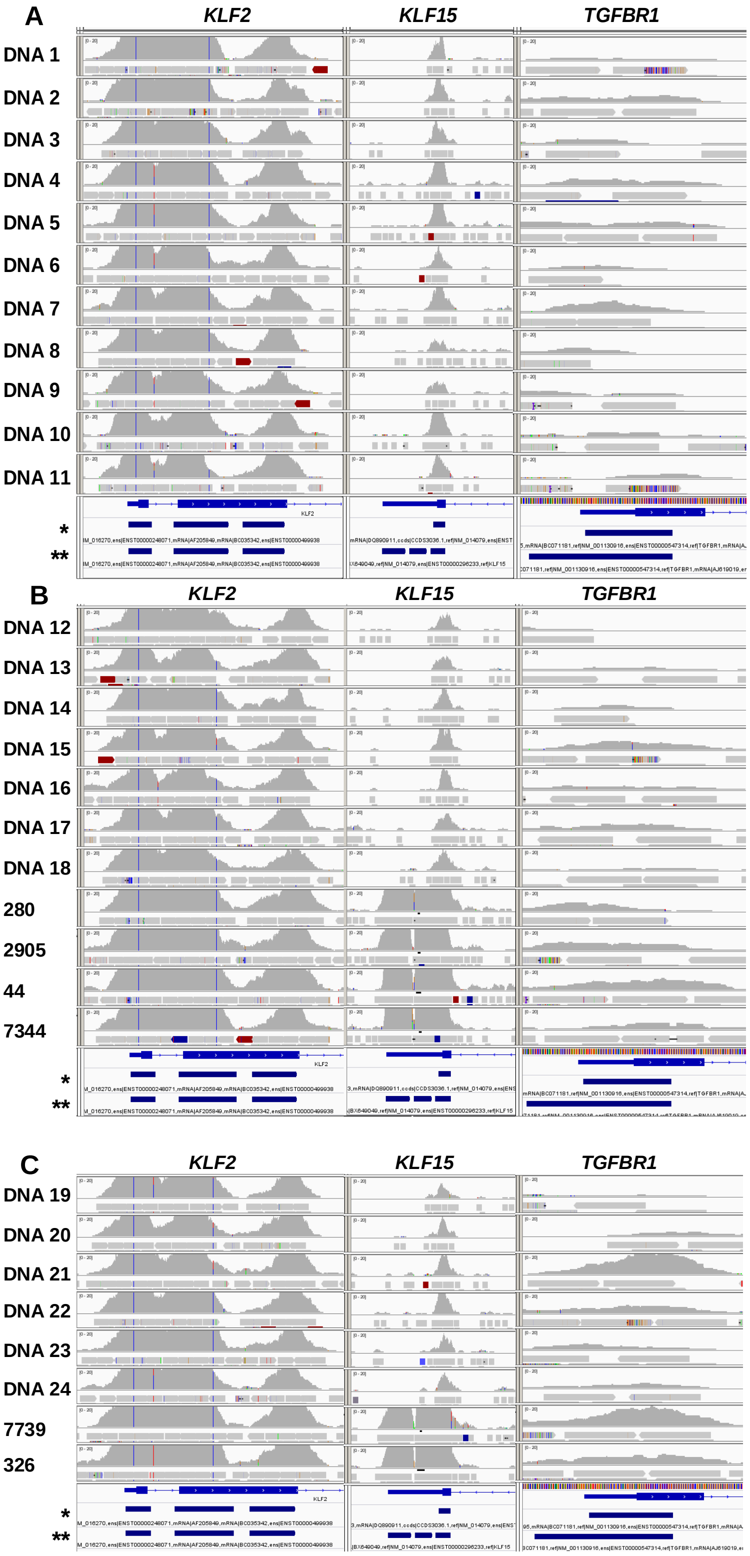

Although DNA samples extracted from blood performed slightly better, no distinct performance difference among the 24 additional DNA samples sequenced at 60× using Agilent was observed in selected GC-rich exons of *KLF2*, *KLF15*, and *TGFBR1*, confirming our observations on the six samples sequenced at 100× using Agilent. Unexpectedly, for one extremely GC-rich (83%) exon, which was not covered sufficiently even when sequenced at 100×, one DNA extracted from saliva and sequenced at 60× achieved a mean of 18 reads (*TGFBR1* in DNA 21).

Figure S3. Comparison of enrichment efficiency for GC-rich exons in the six samples of the present study and 24 additional DNA samples. Integrative Genomics Viewer (IGV) print screens showing coverage track (0-20 reads) for *KLF2* exons 1 and 2, *KLF15* exon 3, and *TGFBR1* exon 1, which have high GC content. (**A**, **B**) DNA samples extracted from blood. (**C**) DNA samples extracted from saliva (DNA 19-24, 7739) or fibroblasts (326). For the six samples of this study (44, 280, 326, 2905, 7344, 7739), WES data of V2 using Agilent SureSelect v5+UTR at 100× are shown. The additional 24 samples (DNA 1-24) were sequenced by V2 using Agilent SureSelect v5 at 60×. *, designed target region of Agilent SureSelect v5; **, designed target region of Agilent SureSelect v5+UTR.

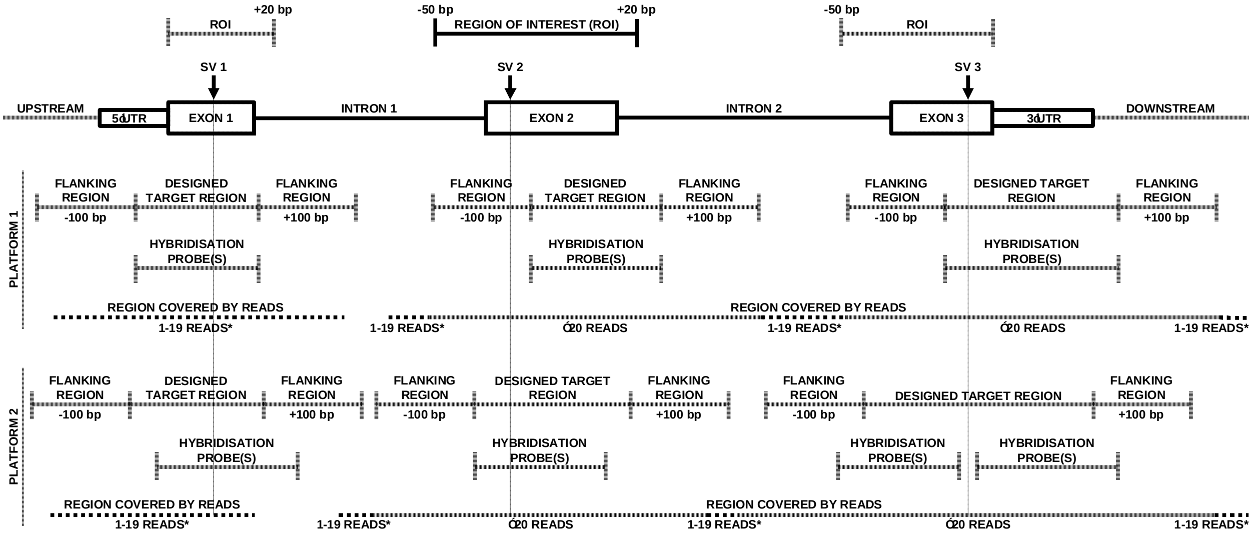

Figure S4. Schematic presentation of terms used in this study. SV, sequence variant; *insufficient coverage e.g. due to GC-rich regions or distance to hybridisation probes; closed lines, regions with defined length and positions; open lines, regions with variable length and positions. Note that according to Agilent's definition the designed target region is completely covered by hybridisation probes (cf. platform 1), whereas NimbleGen and Illumina define it as the region intended/designed to be enriched (cf. platform 2).

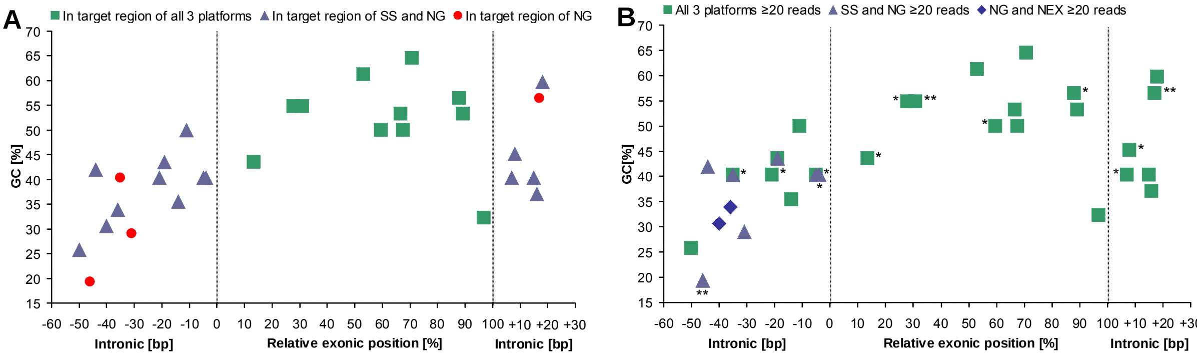

Figure S5. Positions of selected heterozygous sequence variants detected by Sanger sequencing in a region of interest most relevant for mutation screening (exons with -50-bp and +20-bp flanking intronic sequences) as a function of the GC content of 30-bp flanking sequences. (**A**) Occurrence of variants in the designed target region of each platform. (**B**) Platforms providing ≥20 reads at heterozygous variant positions (results per vendor are shown in Supplementary Figure S6). Note that all exonic positions are in designed target regions and completely covered by all three enrichment platforms with ≥20 reads and that Agilent and particularly Illumina covered variants outside its designed target region with ≥20 reads. Exonic positions are given in percentages [%] relative to the length of the corresponding exon, whereas intronic positions are given as absolute positions in base pairs [bp]. SS, Agilent SureSelect v5+UTR; NG, NimbleGen SeqCap v3+UTR; NEX, Illumina Nextera Expanded Exome; *, heterozygous in one additional DNA sample with the same symbol; **, heterozygous in two additional DNA samples with the same symbol.

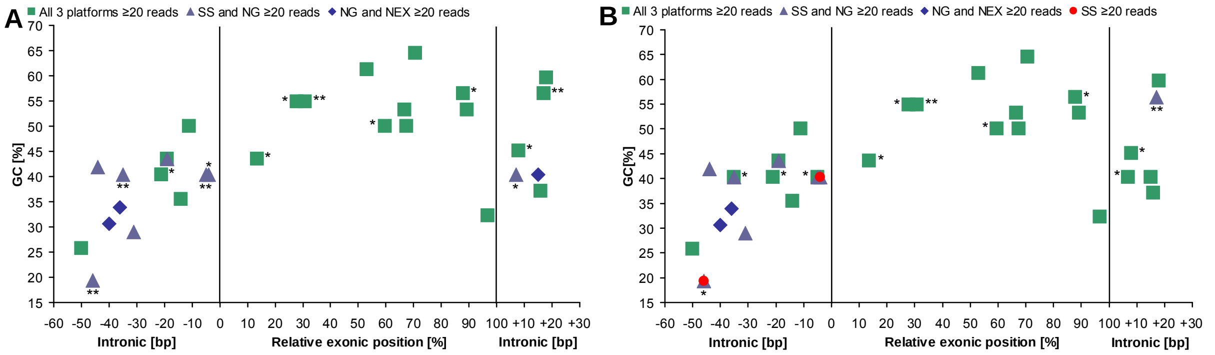

Figure S6. Enrichment performance per vendor for selected heterozygous sequence variants detected by Sanger sequencing in our region of interest (exons with -50-bp and +20-bp flanking intronic sequences) as a function of the GC content of 30-bp flanking sequences. (**A**) Platforms performed by the same vendor (V1) providing ≥20 reads at heterozygous variant positions. (**B**) Platforms performed by different vendors (V2-V4) providing ≥20 reads at heterozygous variant positions. Note that all exonic positions are completely covered by all three enrichment platforms with ≥20 reads (regardless of vendor). Exonic positions are given in percentage [%] relative to the length of the corresponding exon, whereas intronic positions are given as absolute positions in base pairs [bp]. SS, Agilent SureSelect v5+UTR; NG, NimbleGen SeqCap v3+UTR; NEX, Illumina Nextera Expanded Exome; *, heterozygous in one additional DNA sample with the same symbol; **, heterozygous in two additional DNA samples with the same symbol.

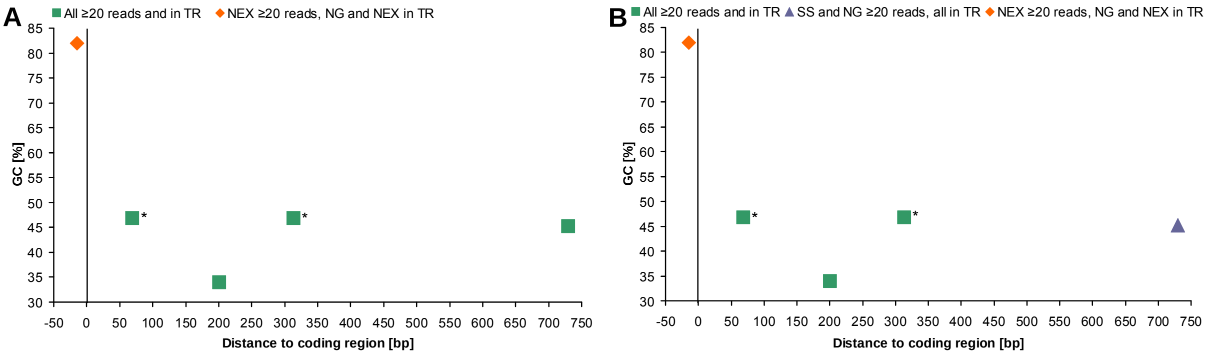

Figure S7. Enrichment performance per vendor for DNA samples with selected heterozygous sequence variants detected by Sanger sequencing in UTR as a function of the GC content of 30-bp flanking sequences. (**A**) Results of the same vendor for all platforms (V1). (**B**) Data of different vendors (V2-V4). Positions of variants are given as distance to coding region in base pairs [bp] (i.e. -50 to -1 for 5'UTR and 1 to 750 for 3'UTR). TR, designed target region; SS, Agilent SureSelect v5+UTR; NG, NimbleGen SeqCap v3+UTR; NEX, Illumina Nextera Expanded Exome; *, heterozygous in one additional DNA sample with the same symbol. Note that one variant position in a GC-rich (82%) exon (orange) is located within the designed target region of both NimbleGen (NG) and Illumina (NEX) but is only covered by Illumina (NEX) at 20× (regardless of vendor).

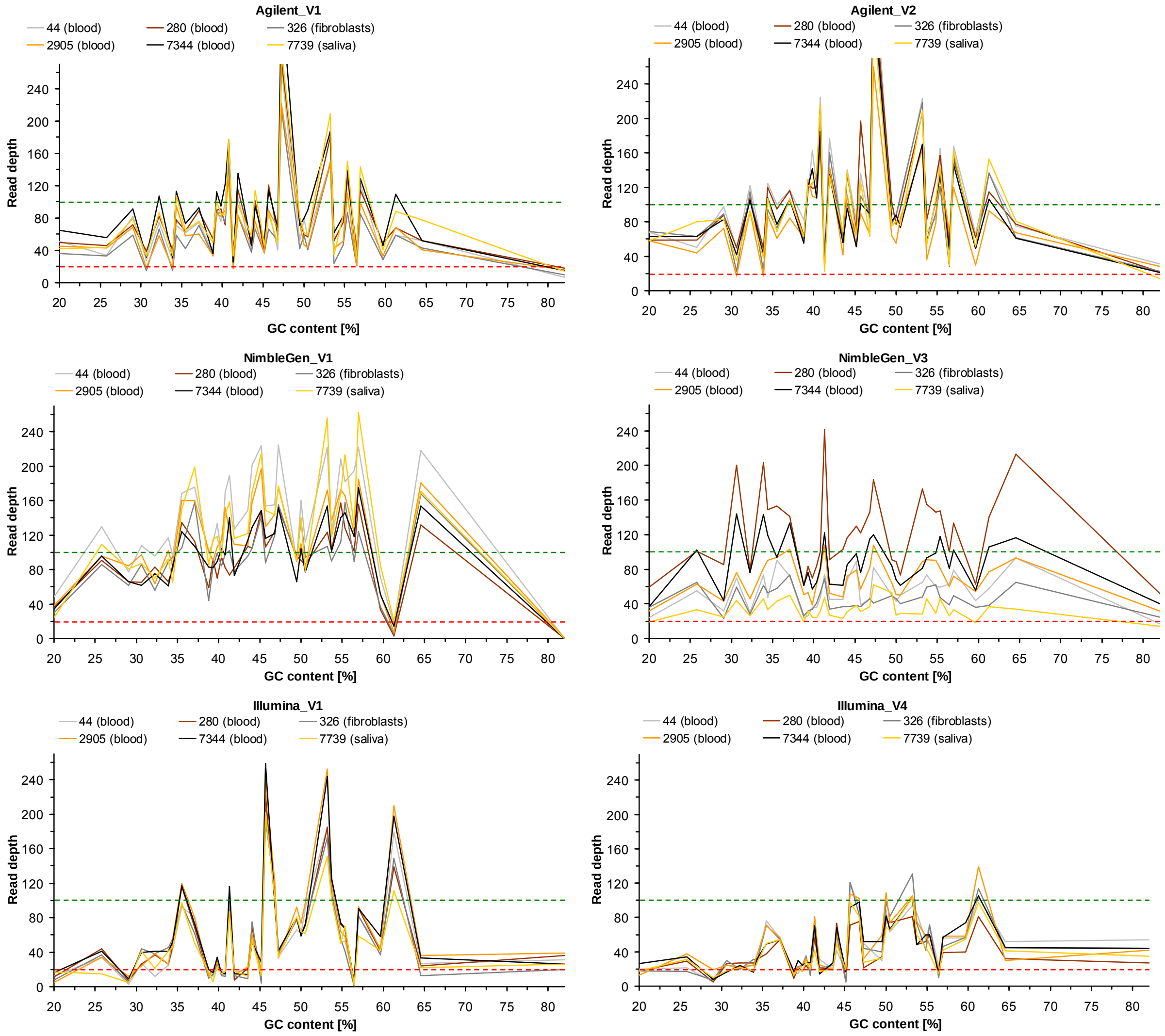

Figure S8. Read depth of all six DNA samples for 30 different SNV positions within our region of interest (coding exons and -50-bp and +20-bp flanking intronic sequences) and five in UTR plotted against the GC content of 30-bp flanking sequences. Green dashed lines indicate expected read depth of 100 (reads) and red dashed lines denote read depth of 20 (reads). Note the performance of Agilent and NimbleGen compared to Illumina as well as the intersample variation in the performance of V3 using the NimbleGen platform.

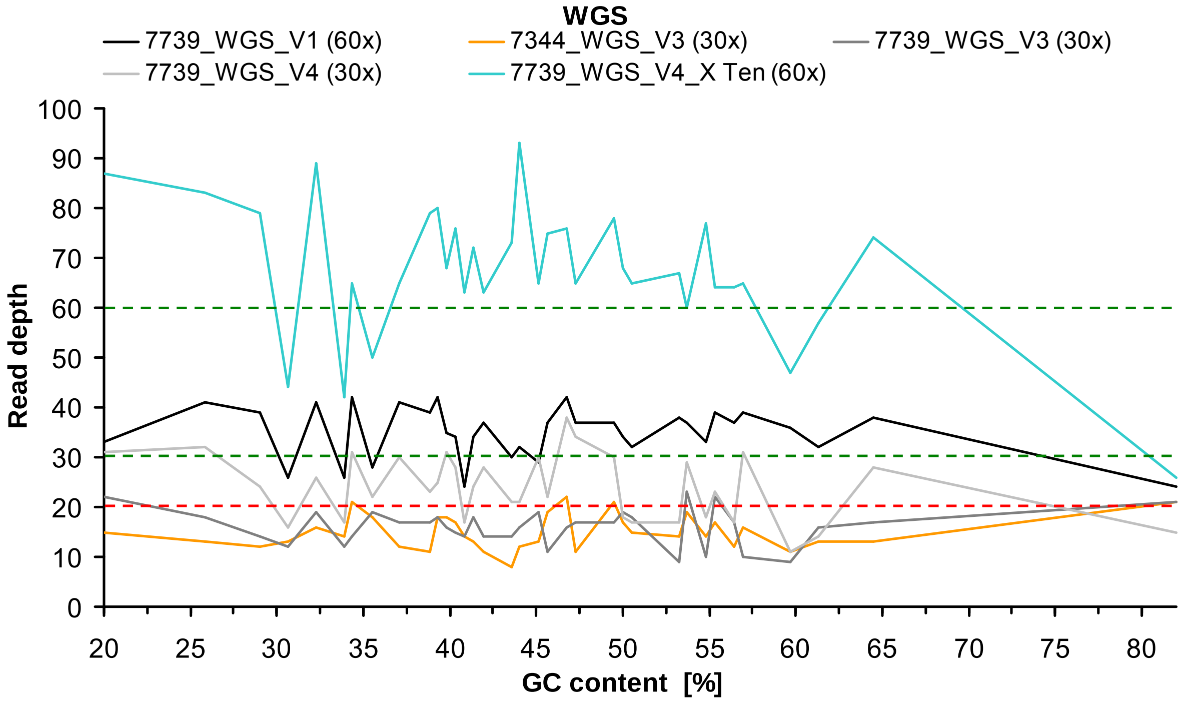

Figure S9. Read depth of all five WGS data sets for 30 different SNV positions within our region of interest (coding exons and -50-bp and +20-bp flanking intronic sequences) and five in UTR plotted against the GC content of 30-bp flanking sequences. Green dashed lines indicate expected read depth of 60 and 30 (reads), respectively, and red dashed line denotes read depth of 20 (reads). Note that at 30× V4 performed better than V3 and that only WGS data at 60× (V1 and V4_X Ten) reached for all positions the limit of 20 reads (cf. Supplementary Figure S17). X Ten, HiSeq X Ten system.

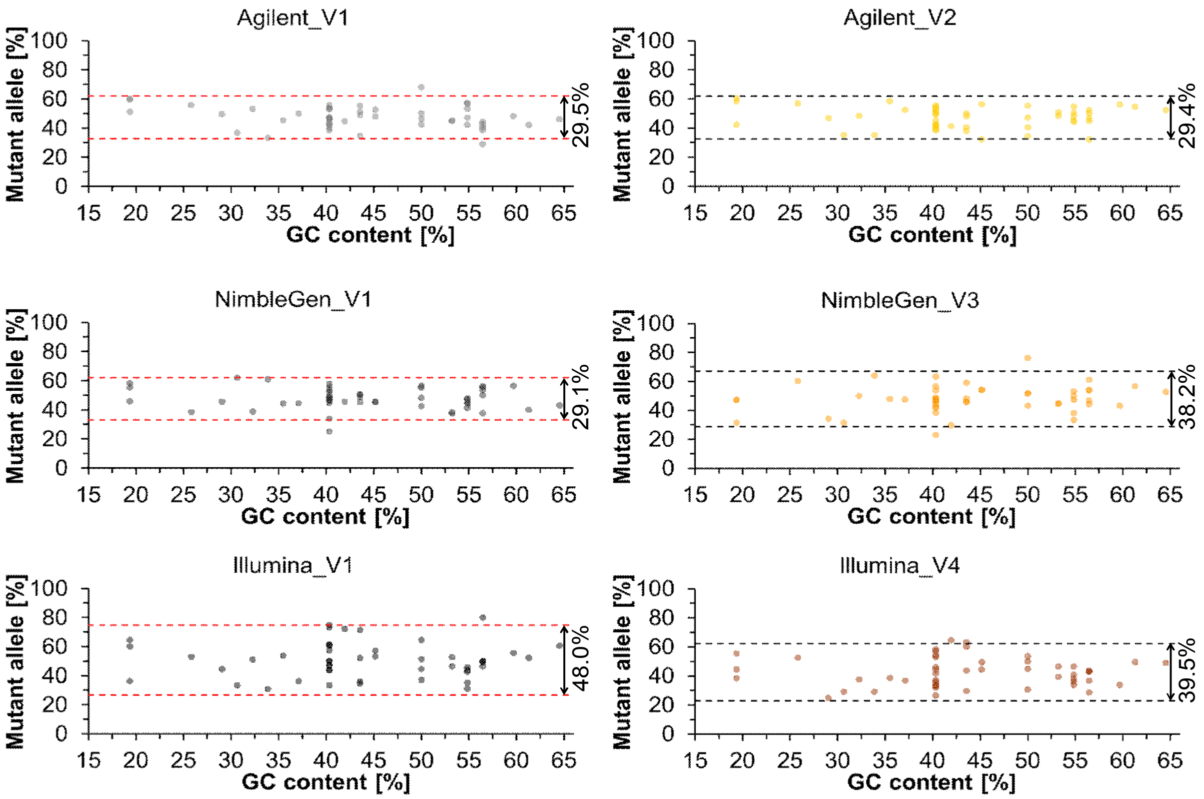

Figure S10. Percentage of non-reference (mutant) alleles called for heterozygous variants detected by Sanger sequencing in our region of interest (exons with -50-bp and +20-bp flanking intronic sequences) displayed for all six platform-vendor combinations. 30 different heterozygous SNVs listed according to GC content of 30-bp flanking sequences. Shown are values of all six DNA samples. Dashed lines indicate an interval within which 95% of the percentage values of non-reference alleles lie (calculated according to the Student's t distribution as the mean of n percentage values ± critical t value ($t_{crit,n-1}$) × SD using n = 49, t_{crit} = 2.011).

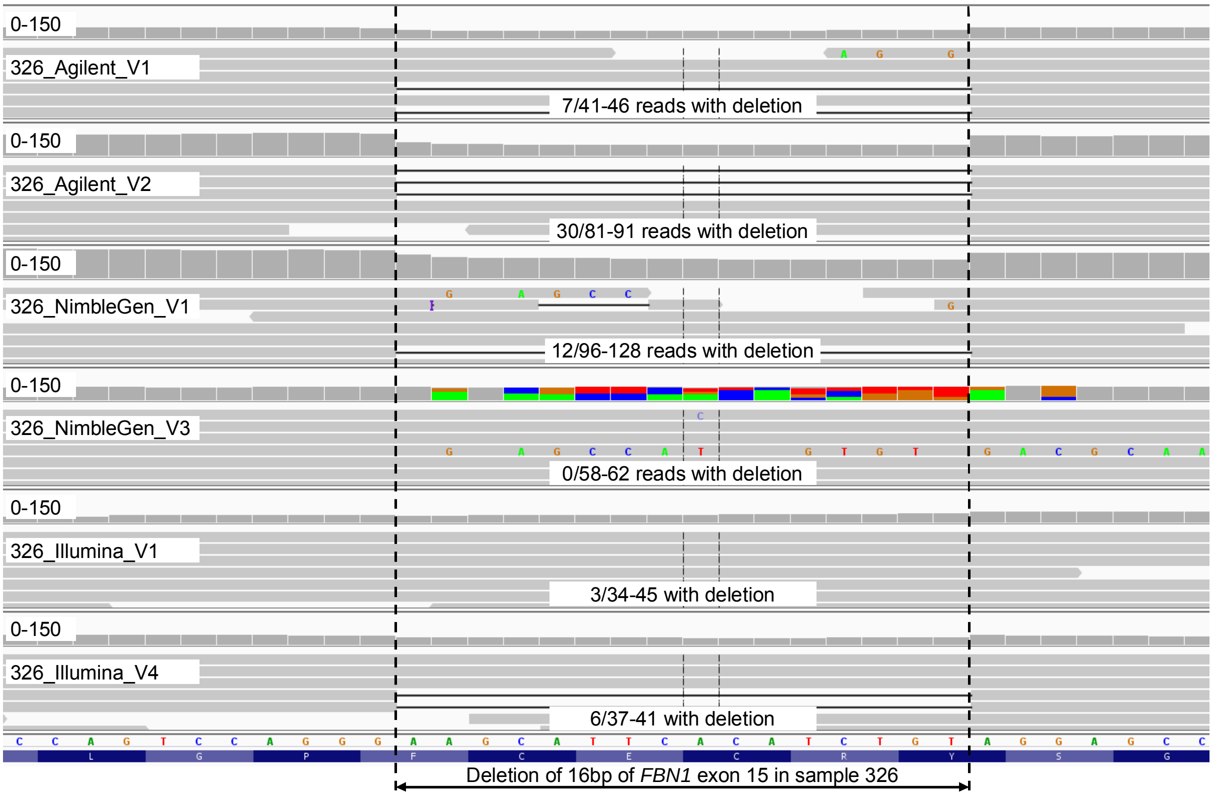

Figure S11. Aligned reads for a heterozygous exonic deletion of 16 bp. Sections of six reads per data set covering the deleted region in sample 326 are presented using the Integrative Genomics Viewer (IGV). Gray bars/arrows denote aligned reads, letters indicate mismatched bases, and black horizontal lines represent deletions. Display range for read depth is set to 0-150 and counts only called bases (no deletions). Coloured bars indicate called mismatches. Note that in the data of V3 using NimbleGen the deleted alleles are called as a series/consecutive of mismatches rather than a deletion, most likely due to the bioinformatics data analysis workflow of V3 (e.g., improper realignment of the region) and that V2 using Agilent called the largest fraction of reads with this deletion.

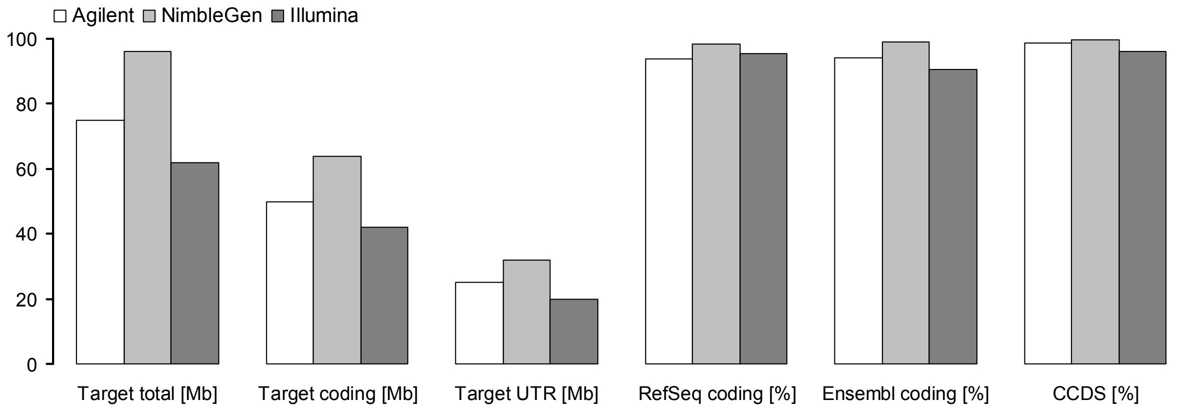

Figure S12. Designed target regions of the three updated exome enrichment platforms. Total number of bases within the designed target region (target total), its distribution on coding (target coding) and untranslated regions (target UTR), and coverage of coding exons of the gene databases RefSeq, Ensembl, and CCDS are presented for Agilent (SureSelect v5+UTR), NimbleGen (SeqCap v3+UTR), and Illumina (Nextera Expanded Exome) according to the companies' specifications (Supplementary Table S2). CCDS, Consensus Coding Sequences.

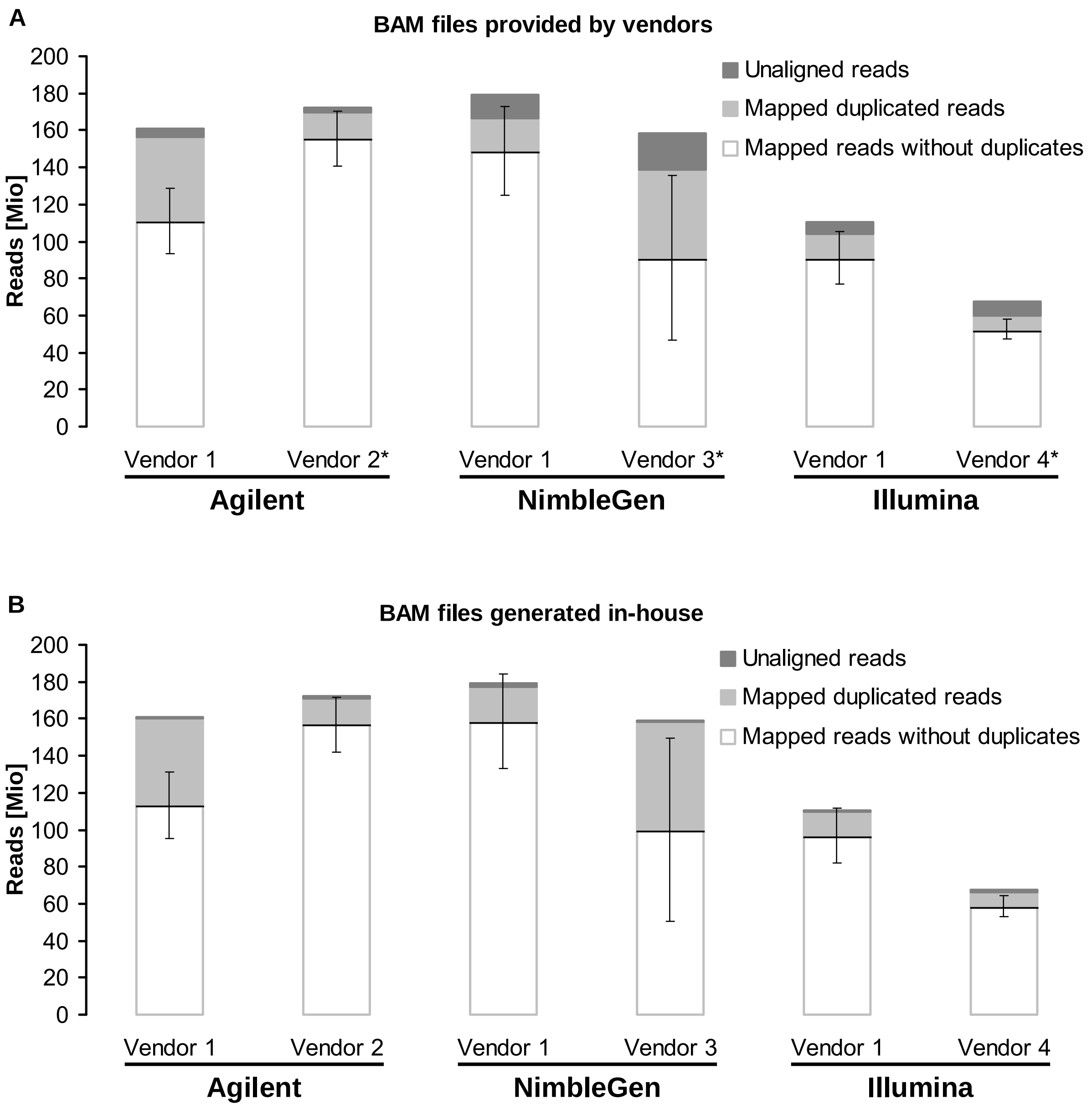

Figure S13. Reads generated using the three different enrichment platforms (Agilent, NimbleGen, and Illumina) applied by different vendors. (**A**, **B**) Number of unaligned reads, mapped duplicated reads, and remaining reads used for analysis (mapped reads without duplicates) according to BAM files provided by vendors (**A**) and in-house generated BAM files using the same bioinformatics pipeline (**B**). *Note that bioinformatics workflow is different among vendors and that vendor 3 and vendor 4 provided unique mapped reads in contrast to total mapped reads (cf. Supplementary Table S3). Given are means of all six DNA samples (n=6); error bars indicate 95% confidence intervals.

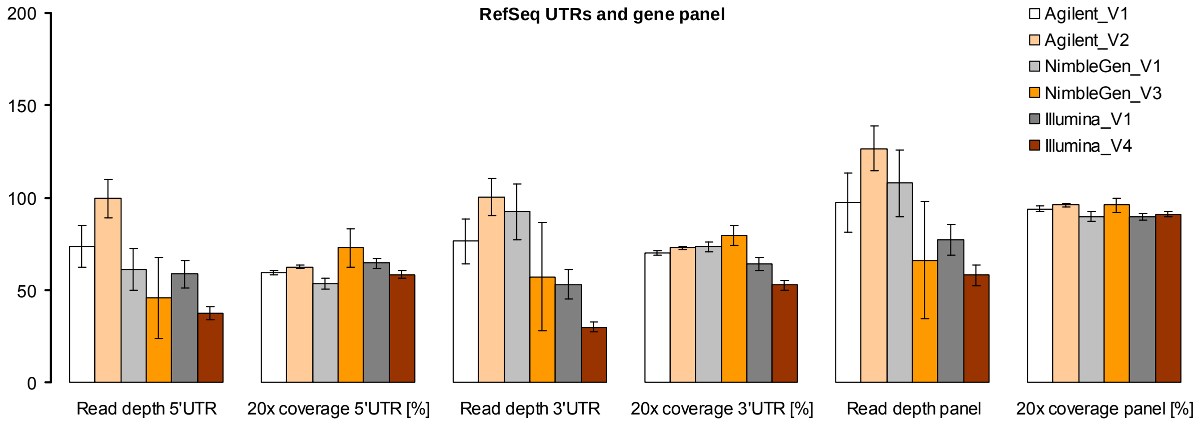

Figure S14. Enrichment efficiency of the three updated exome enrichment platforms (Agilent, NimbleGen, and Illumina) performed by different vendors (V1, V2, V3, and V4) for UTR of RefSeq exons and a panel of eight genes (cf. Supplementary Figure S27 for data on a larger set of clinically relevant exons). Mean read depth and percentage of coverage at 20× for 5'UTR and 3'UTR of the RefSeq database as well as for exons (coding and UTR) of our panel of eight genes (panel) specified in Supplementary Table S1. Given are means of all six DNA samples; error bars indicate 95% confidence intervals. Values were calculated using the SeqMonk program (www.bioinformatics.babraham.ac.uk/projects/seqmonk) and are presented in Supplementary Tables S6, S12, S14, and S15.

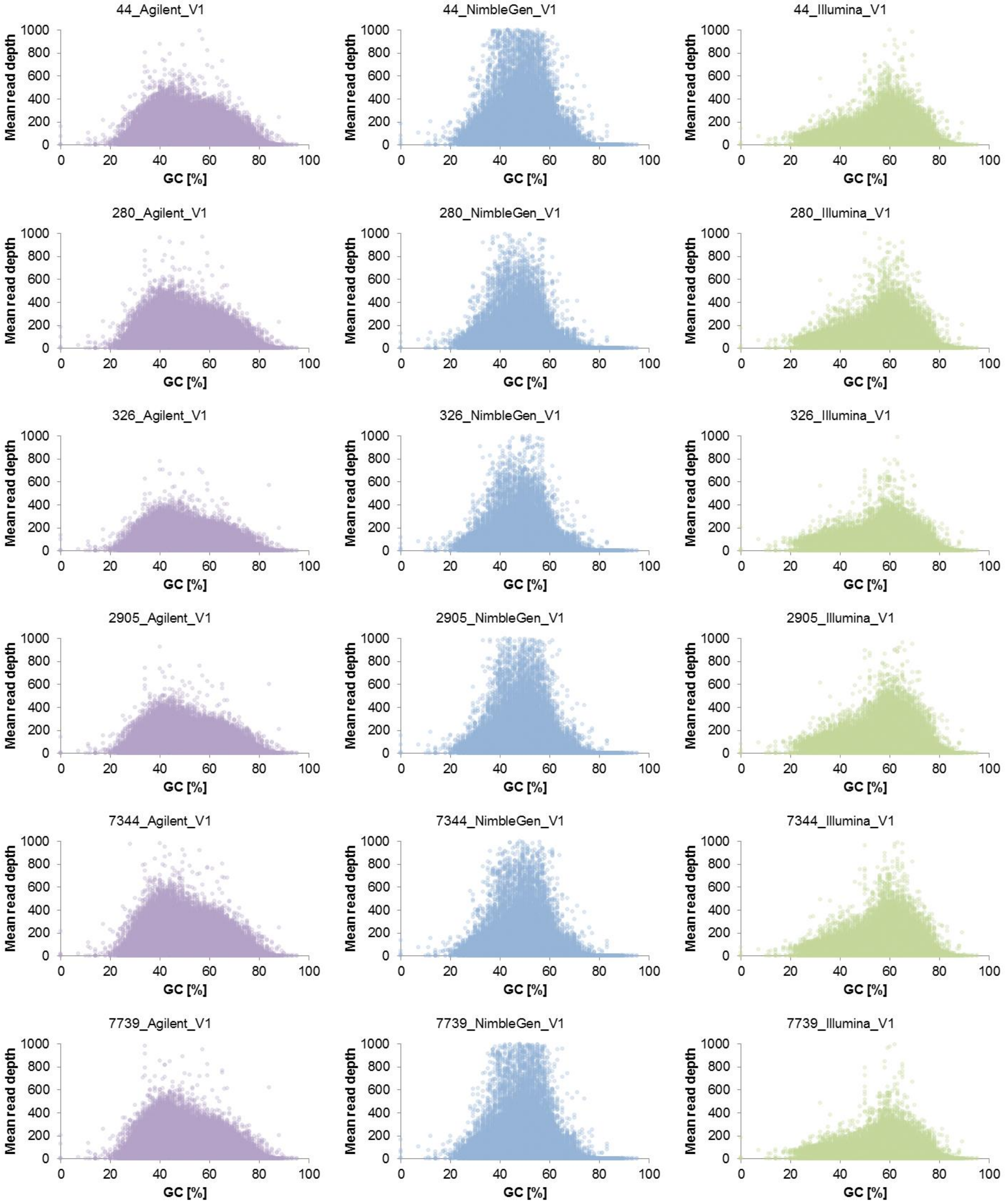

Figure S15. Enrichment bias in terms of read depth owing to GC content for the three enrichment platforms (Agilent, NimbleGen, Illumina) performed by the same vendor (V1). Scatter plots showing GC content and achieved read depth of RefSeq exons (coding and UTR).

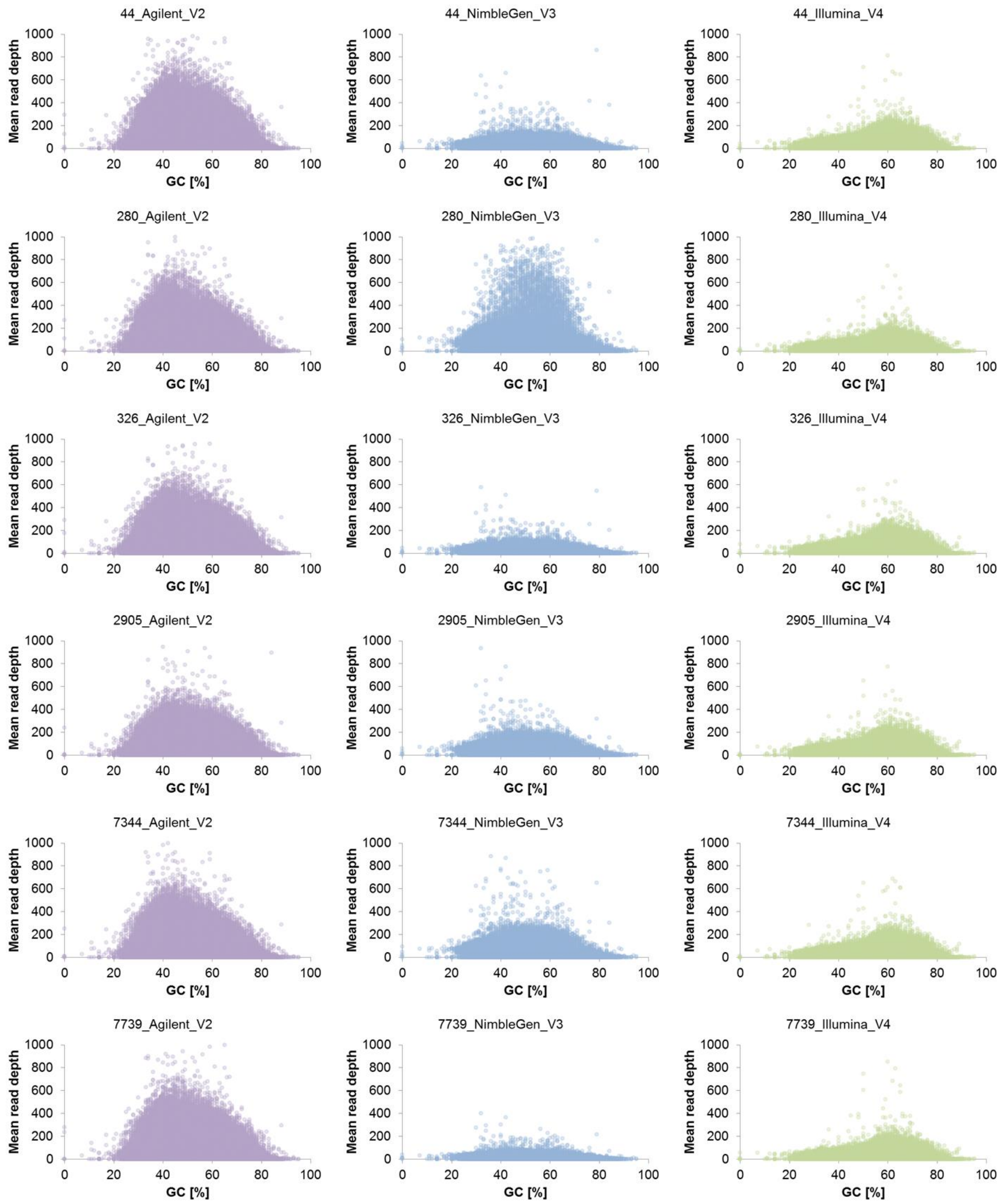

Figure S16. Enrichment bias in terms of read depth owing to GC content for the three enrichment platforms (Agilent, NimbleGen, Illumina) performed by different vendors (V2-V4). Scatter plots showing GC content and achieved read depth of RefSeq exons (coding and UTR).

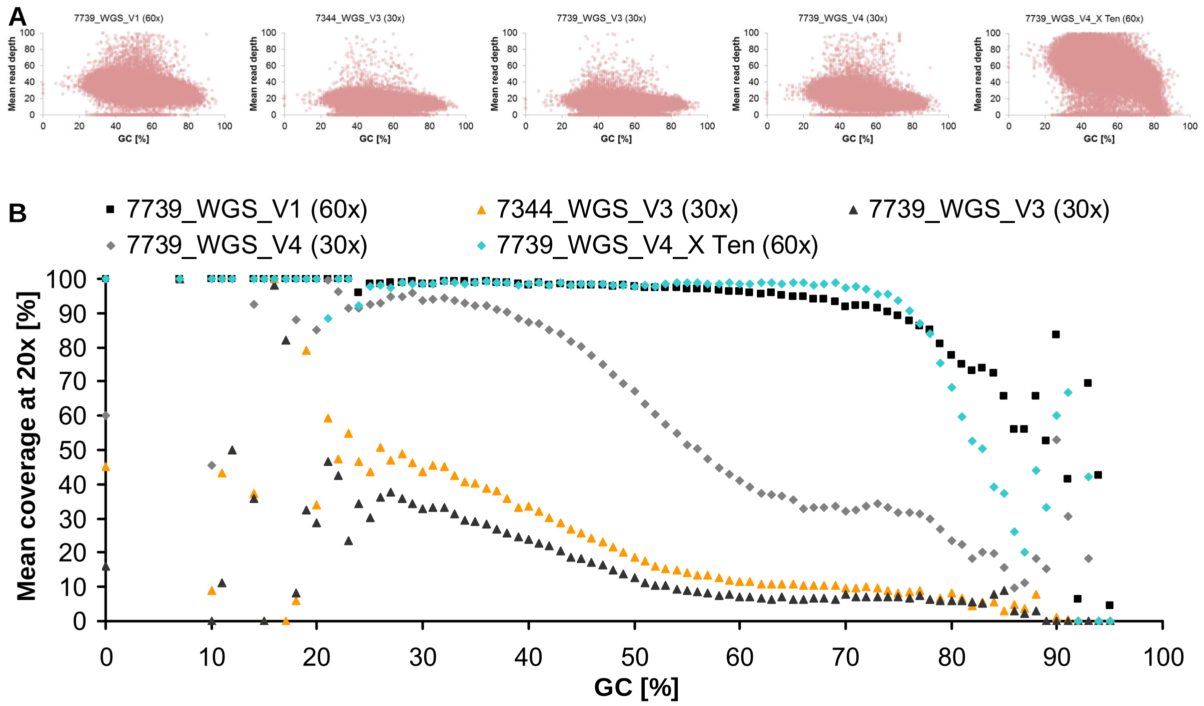

Figure S17. Influence of GC content on the mean read depth and coverage of RefSeq exons in whole genome sequencing (WGS). (**A**) Scatter plots showing GC content and achieved mean read depth. (**B**) Mean coverage at 20× per GC content. Data are shown for RefSeq exons (coding and UTR) for all five WGS data sets, i.e. for 7739 sequenced by V1 at 60× and by V3 and V4 at 30× as well as for 7344 sequenced by V3 at 30× on HiSeq2000/2500 using Illumina's TruSeq DNA PCR-Free Sample Preparation Kit and for 7739 sequenced by V4 at 60× on a HiSeq X Ten system using Illumina's TruSeq Nano DNA Sample Preparation Kit. Note: GC-rich exons were better covered by WGS than by WES, thereby WGS performed by V3 resulted in comparable coverage for both samples in spite of different DNA sources, whereas V4 showed better WGS performance at 30× than V3 and, as expected, WGS at 60× was superior to sequencing at 30× (cf. PCR-free sample preparation (V1) performed better than TruSeq Nano (V4_X Ten), especially above 80% GC content).

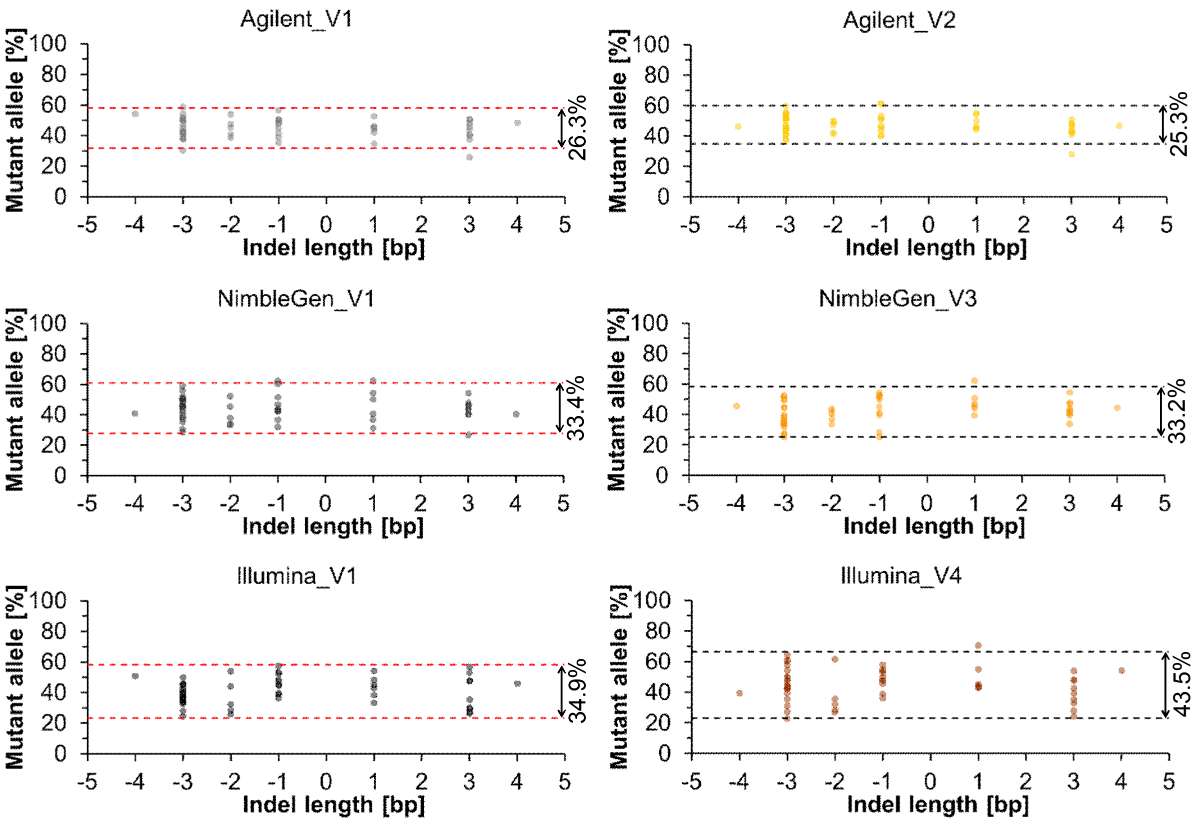

Figure S18. Relative proportion of non-reference (mutant) alleles for indels called in the VCF files provided by vendors (V1-V4). The analysis was restricted to shared heterozygous variants within the designed target regions of the three platforms (Agilent, NimbleGen, and Illumina) located in exons completely (100%) covered at 20× by all six platform-vendor combinations. Heterozygous indels listed according to their length, thereby negative and positive values indicate deletions and insertions, respectively. Shown are values of all six DNA samples. Dashed lines indicate an interval within which 95% of the percentage values of non-reference alleles lie (calculated according to the Student's *t* distribution as the mean of *n* percentage values ± critical *t* value ($t_{crit,n-1}$) × *SD* using n = 51, t_{crit} = 2.009).

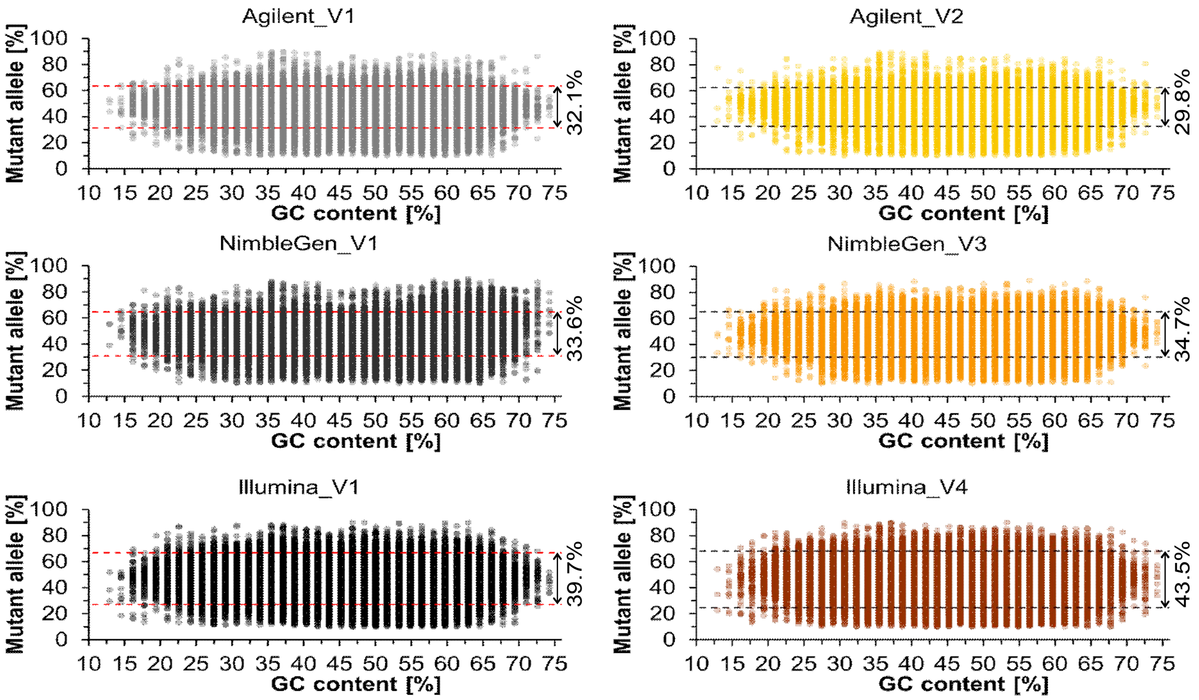

Figure S19. Percentages of non-reference (mutant) alleles for heterozygous SNVs called in our gVCF files generated by the same in-house bioinformatics pipeline for all six platform-vendor combinations. Fraction of biallelic non-reference alleles for shared heterozygous variants within the platforms' designed target region and 50-bp flanking sequences achieving ≥20 reads and >30 quality scores by all six platform-vendor combinations are displayed according to the GC content of 30-bp flanking sequences. Shown are values of all six DNA samples. Variant positions with more than one different non-reference allele (non-biallelic) as well as variant calls with alternative allele percentages outside 10-90% were excluded. Dashed lines indicate an interval within which 95% of the percentage values of non-reference alleles lie (calculated according to the Student's *t* distribution as the mean of *n* percentage values ± critical *t* value ($t_{crit,n-1}$) × *SD* using n = 92'158, t_{crit} = 1.960).

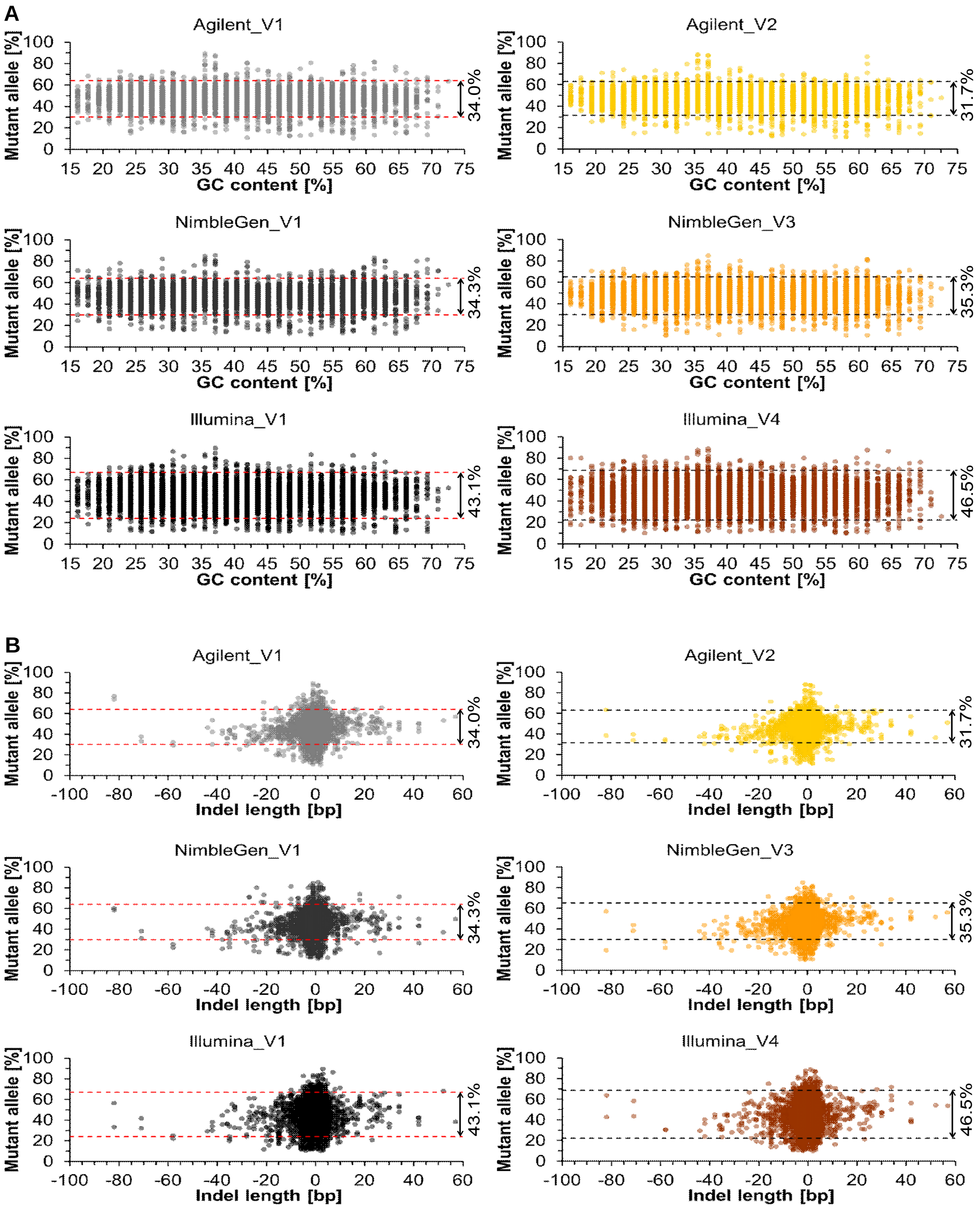

Figure S20. Percentages of non-reference (mutant) alleles for heterozygous indels called in our gVCF files generated by the same in-house bioinformatics pipeline for all six platform-vendor combinations. Fraction of biallelic non-reference alleles for shared heterozygous variants within the platforms' designed target region and 50-bp flanking sequences achieving ≥20 reads and >30 quality scores by all six platform-vendor combinations are displayed. (**A**) Diagrammed according to the GC content of 30-bp flanking sequences. (**B**) Diagrammed according to their length, thereby negative and positive values indicate deletions and insertions, respectively. Shown are values of all six DNA samples. Variant positions with more than one different non-reference allele (non-biallelic) as well as variant calls with alternative allele percentages outside 10-90% were excluded. Dashed lines indicate an interval within which 95% of the percentage values of non-reference alleles lie (calculated according to the Student's *t* distribution as the mean of *n* percentage values ± critical *t* value ($t_{crit,n-1}$) × *SD* using *n* = 5'645, t_{crit} = 1.960).

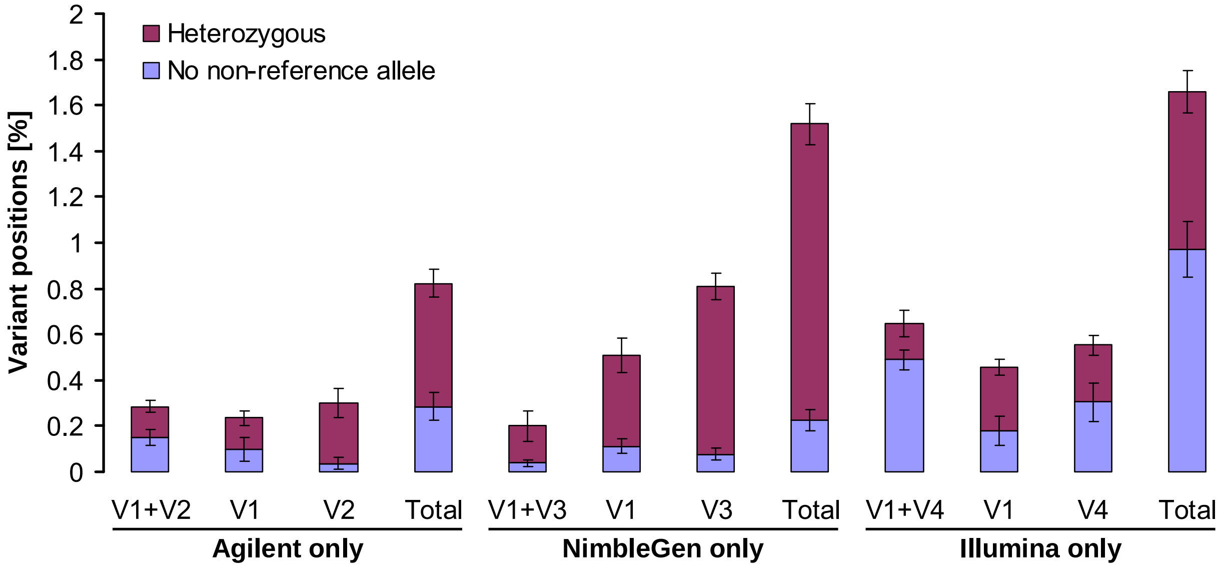

Figure S21. Differences in variant calling among platforms (Agilent, NimbleGen, Illumina) and vendors (V1-V4) in our gVCF files generated by the same in-house bioinformatics pipeline. Displayed are the relative proportions of heterozygous variant positions either called (heterozygous) or missed (no non-reference allele) by only one of the three platforms (i.e. called as heterozygous by only one or by all but one platform). Genomic positions within the platforms' designed target regions and 50-bp flanking sequences which achieved ≥20 reads and >30 quality scores for all six platform-vendor combinations were considered for this analysis (cf. Supplementary Table S3), thereby excluding variant positions with more than one different alternative allele (non-biallelic) or heterozygous calls with allele fractions outside the range of 10-90%. For values see Supplementary Table S28. Error bars indicate 95% confidence intervals.

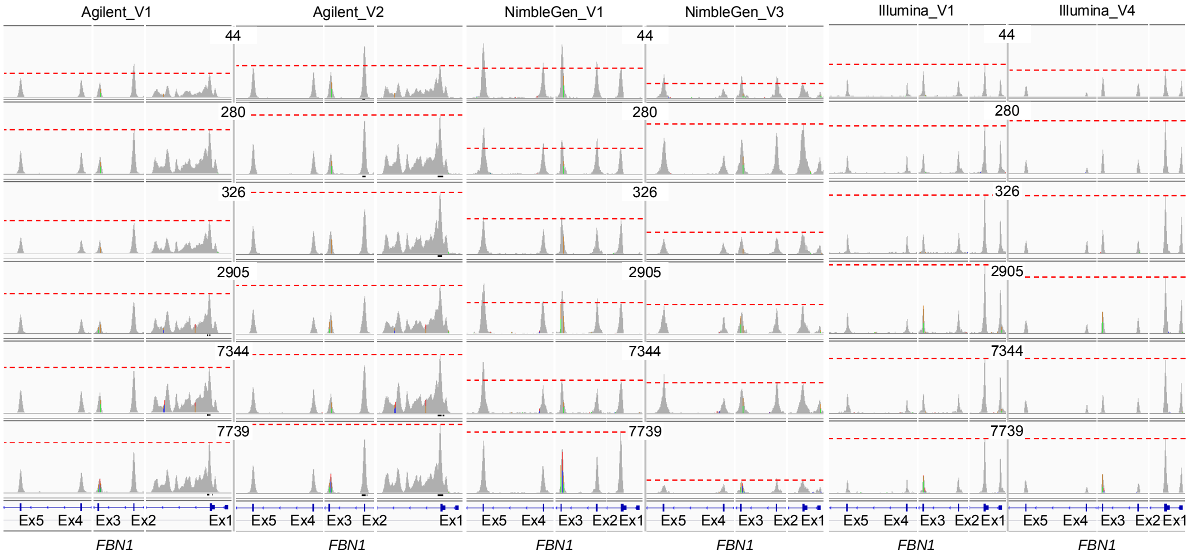

Figure S22. Coverage tracks displaying read depth for exons 1-5 of the *FBN1* gene. Note reduced read depth for deleted exon 1 in sample 44. Red dashed line indicates level of highest read depth of exon 1. Intronic regions between exons 1 and 2 as well as exons 3 and 4 are not shown. Coverage tracks are visualized by the Integrative Genomics Viewer (IGV) and display ranges are set to 0-500 reads for Agilent, 0-200 for NimbleGen, and 0-250 for Illumina.

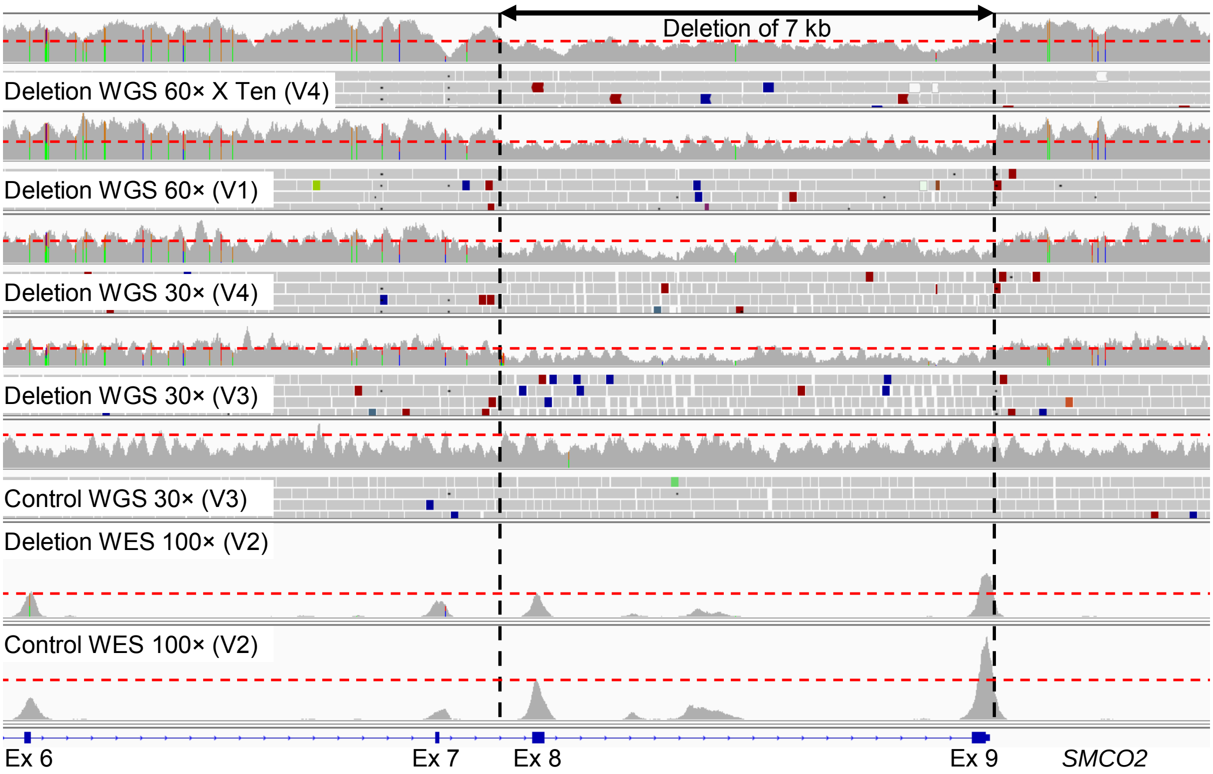

Figure S23. Deletion identification using WGS and WES. The genomic region of a deletion (sample 7739) detected by array CGH (4.2M NimbleGen aCGH, data not shown) is displayed by the Integrative Genomics Viewer (IGV) for both WGS and WES data. WGS was performed by V4 (HiSeq X Ten) and V1 at 60× as well as by V3 and V4 at 30×, whereas WES was carried out by V2 using Agilent SureSelect v5+UTR at 100×. For WES data, coverage track is shown only. Red dashed lines indicate level of highest read depth of exon 8. Display range of coverage tracks are set to 0-100 (V4 HiSeq X Ten), 0-60 (V1), 0-50 (V4), and 0-40 (V3) reads for WGS and 0-350 for WES.

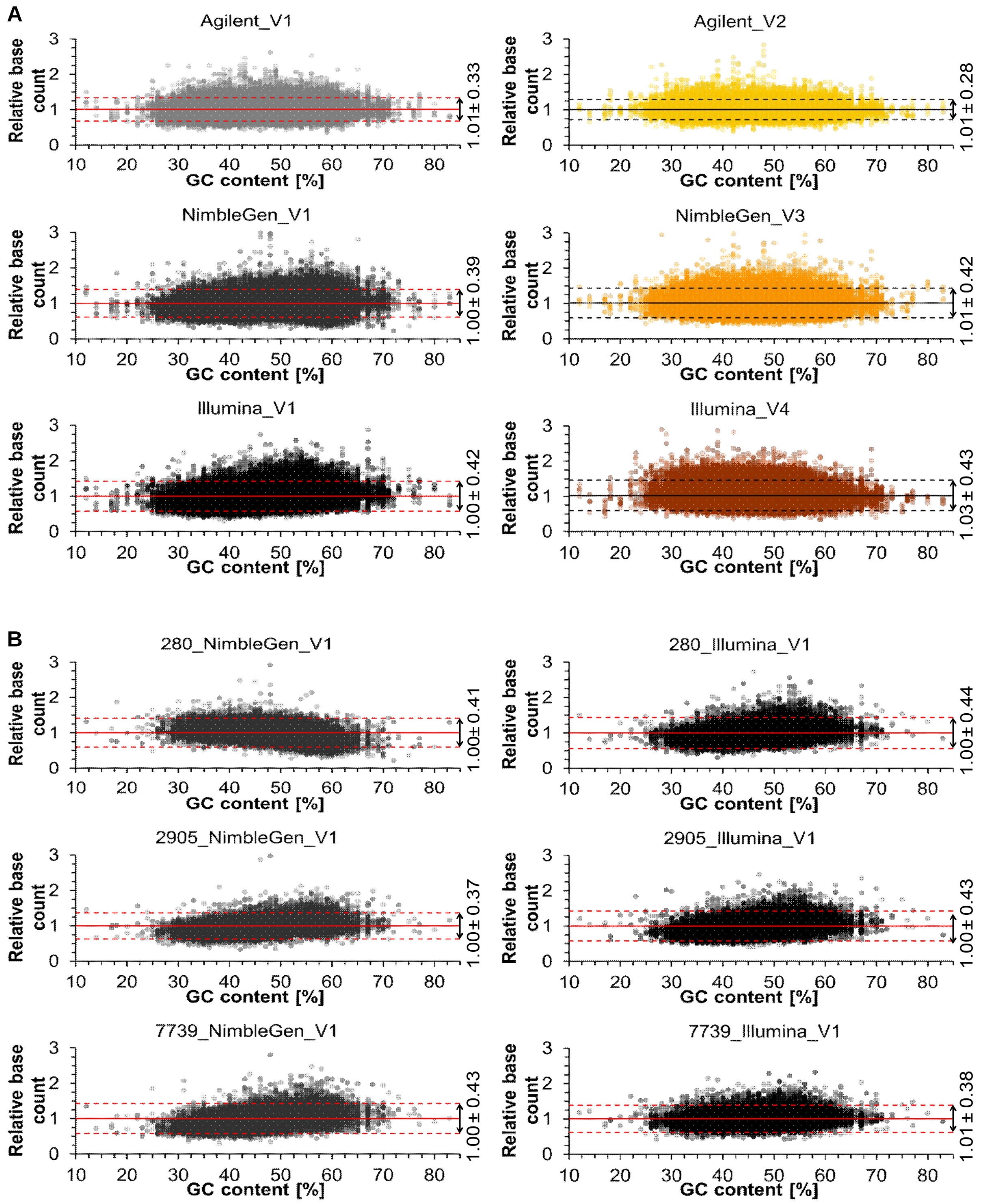

Figure S24. Relative WES base counts of 21’769 RefSeq exons completely (100%) covered at 20× in all 36 platform-vendor-sample combinations (plotted relative to the corresponding base counts of sample 326 and against the GC content of the respective exon). (**A**) Superposition of all five samples (44, 280, 2905, 7344, and 7739) per platform-vendor combination. (**B**) Examples for individual samples (280, 2905, and 7739) of NimbleGen and Illumina both performed by vendor V1 (V1). Note that the distribution of relative base counts for 280_NimbleGen_V1 and 7739_Illumina_V1 differ from the patterns observed in the other two exemplified samples for the same platform-vendor combination. Solid line indicates mean and dashed lines indicate an interval within which 95% of the relative base counts lie (calculated according to the Student's *t* distribution as the mean of *n* values ± critical *t* value ($t_{crit,n-1}$) × *SD* using n = 108’845 and 21’769 in (A) and (B), respectively, t_{crit} = 1.960). Values per sample and the number of dots with relative base counts >3 not shown in this figure are given in Supplementary Table S29.

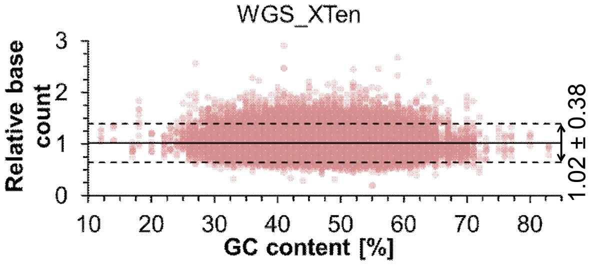

Figure S25. Superposition of the relative WGS base counts of 21’769 RefSeq exons (Supplementary Figure S24) of sample 7739 and four additional DNA samples plotted against the GC content of the respective exon. WGS was performed by V4 at 60× on a HiSeq X Ten system. Solid line indicates mean and dashed lines indicate an interval within which 95% of the relative base counts lie (calculated according to the Student's *t* distribution as the mean of *n* values ± critical *t* value ($t_{crit,n-1}$) × *SD* using n = 108’845, t_{crit} = 1.960). Nine dots have relative base counts >3 and are therefore not shown in this figure.

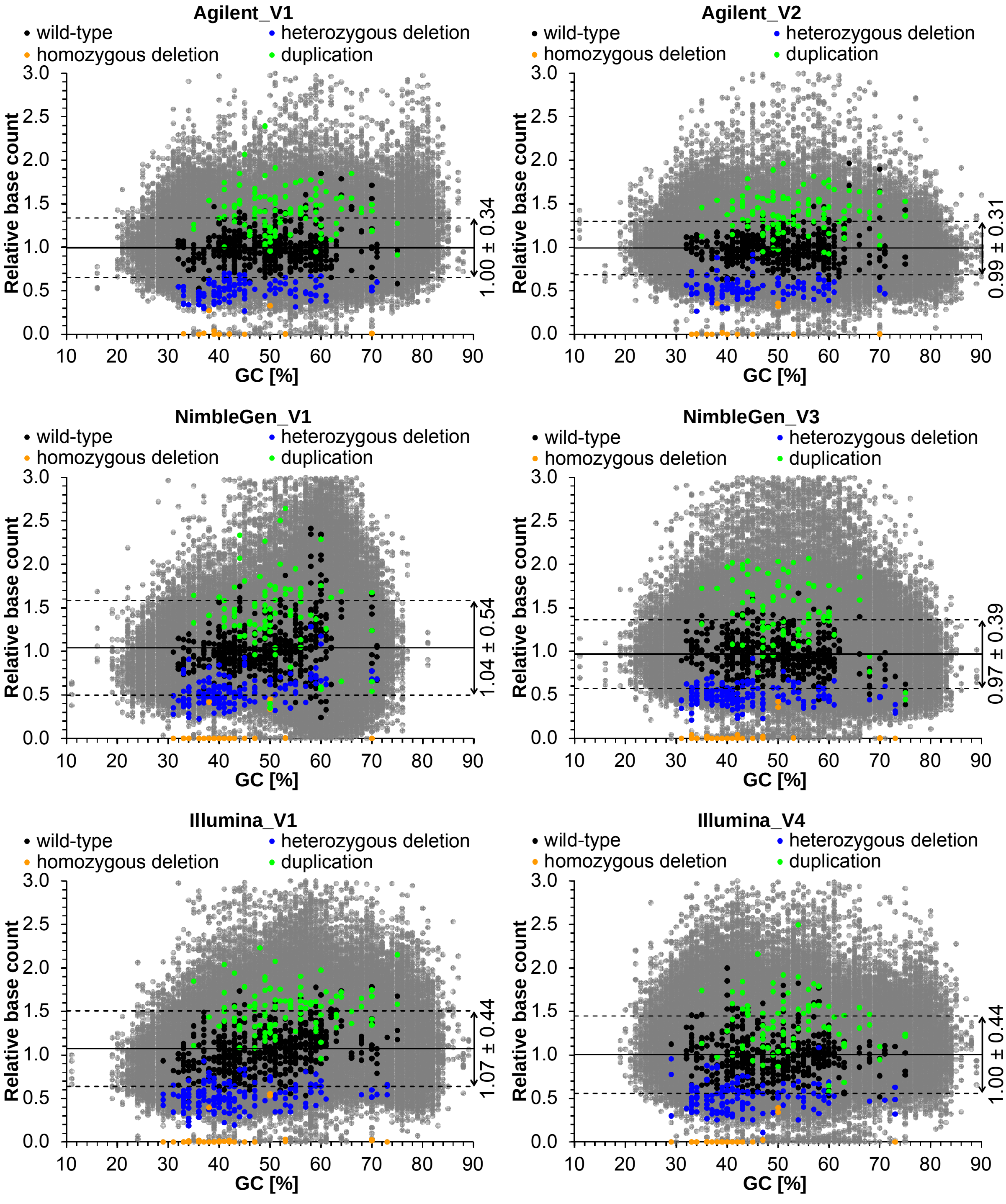

Figure S26. Relative WES base counts of 182 RefSeq exons with copy numbers known from array CGH (black/coloured dots according to the genotype given in Supplementary Table S21). For all six platform-vendor combinations, base counts are normalised by means of 21'769 RefSeq exons (Supplementary Figure S24) and plotted relative to the corresponding base counts of sample 326 and against the GC content of the respective exon. Relative WES base counts of ~160'000 RefSeq exons not used for normalisation with at least one base covered at 20× and a total base count of ≥1'000 in sample 326 are included and indicated (grey dots). Solid line indicates mean of the relative base counts of exons with two copies (wild-type, black dots) and dashed lines indicate an interval within which 95% of the relative base counts of these exons lie (calculated according to the Student's *t* distribution as the mean of *n* values ± critical *t* value ($t_{crit,n-1}$) × *SD* using *n* = 541, 549, 533, 534, 566, and 520 for Agilent_V1, Agilent_V2, NimbleGen_V1, NimbleGen_V3, Illumina_V1, and Illumina_V4, respectively and t_{crit} = 1.960). Values per sample and the number of dots with relative base counts >3 not shown in this figure are given in Supplementary Table S30.

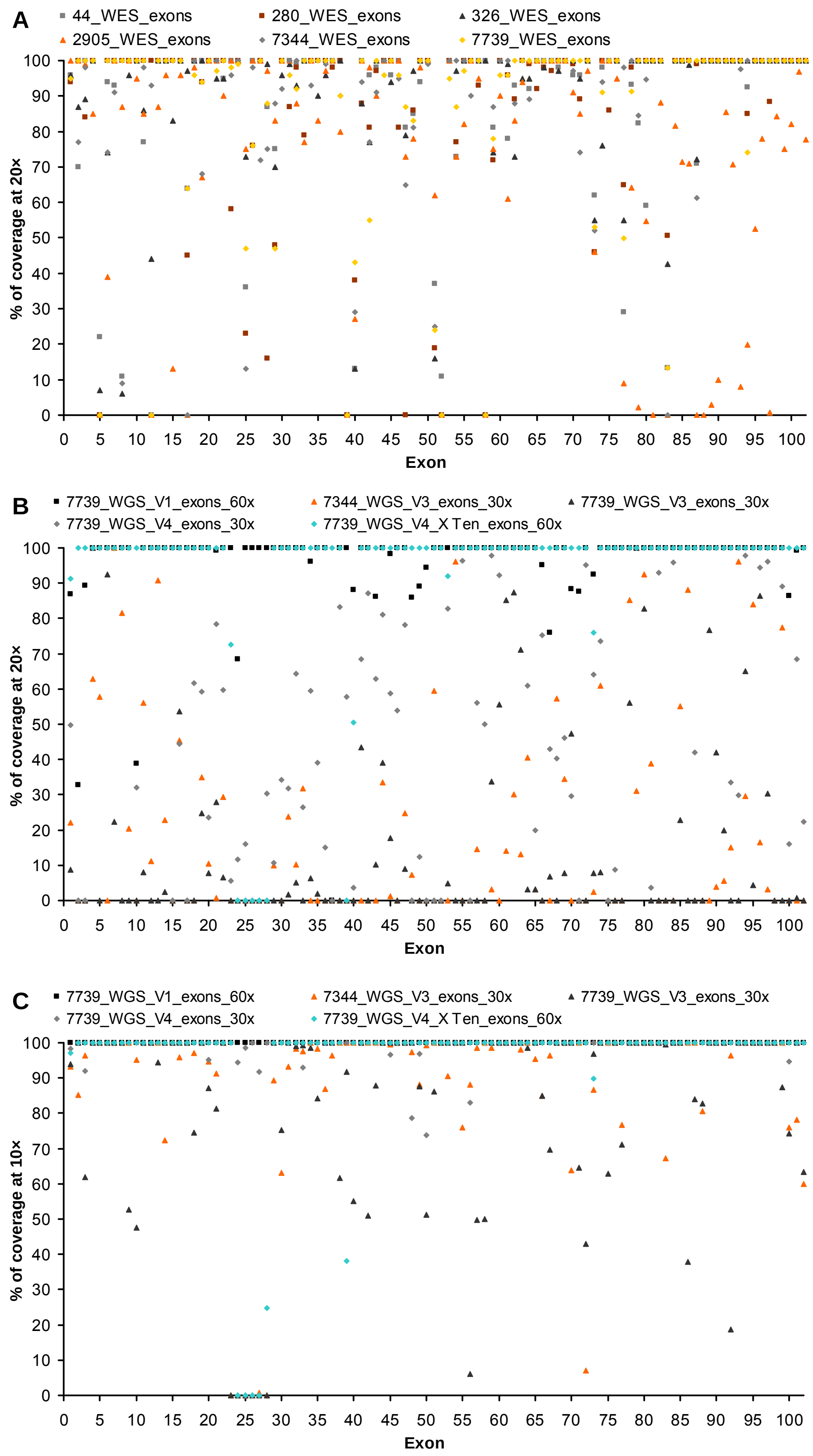

Figure S27. Coverage of selected RefSeq exons of 42 genes affected in monogenic disorders. (**A**) Percentage of coverage at 20× for WES data of the six DNA samples sequenced at 100× by V2 using Agilent. (**B**, **C**) Percentage of coverage at 20× (**B**) and at 10× (**C**) for WGS data of two DNA samples performed by V1 and V4 (HiSeq X Ten) at 60× as well as by V3 and V4 at 30×. Displayed RefSeq exons were covered <100% in at least one WES data set (102 out of 1’113 exons).

Table S1. Panel of eight selected genes and number of variants detected by Sanger sequencing in these genes in at least one of the six DNA samples used in this study. The numbers of heterozygous SNVs and indels (SNVs/indels) are summarized for each gene (all) as well as given for our region of interest (ROI, coding exons with -50-bp and +20-bp flanking intronic sequences) and UTR separately.

		**Number of SNVs/indels**	
**Genes (NM number)**	**All**	**ROI**	**UTR**
COL3A1 (NM_000090)	28/1	11/0	0/0
FBN1 (NM_000138)	35/8	13/4	2/0
FLCN (NM_144997)	0/0*	0/0*	0/0
SLC2A10 (NM_030777)	1/0	1/0	0/0
SMAD3 (NM_005902)	5/0	1/0	1/0
TGFB2 (NM_001135599)	1/0	0/0	1/0
TGFBR1 (NM_004612)	5/2	1/1	1/0
TGFBR2 (NM_003242)	3/0	3/0	0/0
**Total**	**78/11**	**30/5**	**5/0**

*Only two homozygous SNVs, which were excluded from analysis.

Table S2. Details on the design of the three exome enrichment platforms used in this study.

Platform	**Agilent SureSelect Human All Exon kit v5+UTR**			**NimbleGen SeqCap EZ Exome v3+UTR**		**Illumina Nextera Rapid Capture Expanded Exome**	
Bait length (hybridisation temperature)	90/120 bp RNA (65°C)			55-105 bp DNA (47°C)		95 bp DNA (58°C)	
Number of baits	~881'870			2'100'000		340'427	
Bait density	Adjacent, partially overlapping baits across designed target region			High-density overlapping baits; every base covered multiple times		Gaps between the baits	
Region	Designed target region	Hybridisation probes**	Padded***	Designed target region	Hybridisation probes	Designed target region	Hybridisation probes
Total region (coding + UTR)	75 Mb (50 + 25 Mb)	NA	NA	96 Mb (64 + 32 Mb)	NA	62 Mb (42 + 20 Mb)	NA
Calculated total region	***75 Mb***	***73 Mb***	***117 Mb***	***96 Mb***	***99 Mb***	***62 Mb***	***32 Mb***
Number of genes	21'522	NA	NA	>20'000	NA	20'794	NA
Number of exons	359'555	NA	NA	NA	NA	201'121	NA
Coverage of RefSeq coding exons	93.7%*	NA	NA	98.4%	NA	95.3%	NA
Calculated coverage of RefSeq coding exons	***90.1%***	***89.6%***	***98.0%***	***98.3%***	***96.5%***	***94.9%***	***67.0%***
Calculated proportion of RefSeq coding exons 100% covered	***54.2%***	***52.8%***	***97.5%***	***98.3%***	***70.1%***	***94.0%***	***24.0%***
Calculated proportion of RefSeq coding exons 0% covered	***2.4%***	***2.6%***	***1.8%***	***1.6%***	***1.7%***	***4.9%***	***7.2%***
Calculated coverage of 50-bp intronic sequences upstream of RefSeq coding exons	***64.2%***	***63.7%***	***97.1%***	***77.5%***	***86.6%***	***8.7%***	***12.3%***
Calculated coverage of 20-bp intronic sequences downstream of RefSeq coding exons	***70.3%***	***69.7%***	***97.6%***	***93.2%***	***95.2%***	***10.6%***	***18.4%***
Coverage of RefSeq 5'UTR and 3'UTR	71.3%*	NA	NA	NA	NA	>88%	NA
Coverage of Gencode/Encode coding exons	97.9%*	NA	NA	96.7%	NA	91.6%	NA
Coverage of Ensembl coding exons	94.1%*	NA	NA	99.0%	NA	90.6%	NA
Coverage of CCDS exons	98.8%*	NA	NA	99.8%	NA	96.0%	NA
Coverage of miRBase	96.0%*	NA	NA	98.7%	NA	>77.0%	NA
Input gDNA	200 ng (low input), 3 µg (standard input)			1 µg		50 ng	

Information based on documents provided by companies or on correspondence with a company representative as marked by asterisk (*), except for those in bold italic which were calculated from design files downloaded from companies' websites using the SeqMonk program (http://www.bioinformatics.babraham.ac.uk/projects/seqmonk/) and a recent release of the RefSeq database (version December 2013). Note that according to Agilent's definition the designed target region is completely covered by probes, whereas NimbleGen and Illumina define it as the region intended/designed to be enriched, which may explain lower coverage values in Agilent's designed target region (cf. also Supplementary Figure S4). **Some of Aligent's designed target regions are missing in the hybridisation probe file; ***Agilent provides an additional file with a so called padded region including designed target region as well as 100-bp flanking sequences for which sufficient coverage can be expected according to Agilent; CCDS, Consensus Coding Sequences; NA, not available; files for designed target region: Agilent: S04380219_Regions.bed, NimbleGen: track "Target Regions" in 120430_HG19_ExomeV3_UTR_EZ_HX1.bed, Illumina: nexterarapidcapture_expandedexome_targetedregions.bed; files for genomic positions of hybridisation probes: Agilent: S04380219_Probes.txt, NimbleGen: track "Tiled Regions" in 120430_HG19_ExomeV3_UTR_EZ_HX1.bed, Illumina: nexterarapidcapture_expandedexome_probes.txt); file for Agilent's padded region: S04380219_Padded.bed.

Table S3. Data analysis workflow and software used in this study.

	Vendor 1 (V1)* (Agilent, NimbleGen, Illumina)	Vendor 2 (V2)* (Agilent)	Vendor 3 (V3)* (NimbleGen)	Vendor 4 (V4)* (Illumina)	In-house generated gVCF files
Pre-processing	Demultiplexing with CASAVA v1.8.2; adapter clipping; quality trimming (removal of reads containing more than one N; removal of bases or complete reads with sequencing errors; trimming of reads at 3'-end to get a minimum mean Phred quality score of 10 over a window of ten bases; reads with final length <20 bases were discarded)	Read files (FASTQ) generated from the sequencing platform via the manufacturer's proprietary software	CASAVA v1.8.2	RTA v.1.1.2.4; CASAVA v1.8.2	---
Alignment	BWA v.0.7.5a; Picard v1.92 MarkDuplicates; GATK v.1.6-11, GATK resource bundle v2.5 for the human genome release b37: realignment around known and identified indels and recalibration of qualities using known variants to correct for biases	BWA v.0.6.2 (hg19/37); Picard v.1.98 MarkDuplicates; GATK v.1.6: local realignment of the mapped reads around potential indel sites and base quality (Phred scale) scores recalibration (GATK's covariance recalibration); SAMtools v.0.1.18: additional BAM file manipulations	ELANDv2e	BWA, v.0.5.9; SAMtools: extract mappable reads, extract on-target reads****	BWA, v.0.7.10-r789; Picard v1.80 MarkDuplicates; GATK v3.1.1: indel realignment and base quality scores recalibration; SAMtools v0.1.19: BAM file manipulations
Provided/generated BAM files	Unfiltered BAM files with marked duplicates**	Unfiltered BAM files with marked duplicates**	Mapped unique reads without duplicates	Mapped unique on-target reads**** without duplicates	Unfiltered BAM files with marked duplicates**
Variant calling	GATK v.1.6-11, GATK resource bundle v2.5 for the human genome release b37: variant discovery and genotyping using the GATK Unified Genotyper and variant quality score recalibration using known true and false positive variant calls from the 1000 GP	GATK v.1.6: SNP and indel variants called using the GATK Unified Genotyper, dbSNP release 135 to improve quality of calls; SNP novelty determined against dbSNP release 132	CASAVA v1.8.2; annotation using dbSNP, 1000 GP percentage, ESP percentage, and HGMD	SAMtools: extract SNVs and indels, filter SNVs and indels, and annotation; variant databases: dbSNP and 1000 GP	GATK v. 3.1.1: Haplotype Caller
Region for variant calling	Platforms' designed target region	Platforms' designed target region with 100-bp flanking sequences	No restriction	Platforms' designed target region with 100-bp flanking sequences	Platforms' designed target region with 50-bp flanking sequences
Variants/positions in unfiltered VCF files	Quality >30, read depth >1	Quality >30, read depth >1	Quality ≥20, read depth >2	Quality >3, read depth >1	Quality >30, read depth ≥20
Variant filter and recalibration settings in filtered VCF files	SNVs: ABHet >1.0, DP <10, QD <5.0, QUAL <30, and FS >60<br>Indels: DP <10, HRun >5, ReadPosRankSum <-20.0, and FS >200.0<br>Recalibration: TruthSensitivityTranches 99.00 to 99.90, 99.90 to 100+ as well as 99.90 to 100***	SNVs: MQRankSum <-12.5, ReadPosRankSum <-8.0, QD <5.0, MQ <40.0, FS >60.0, HaplotypeScore >13<br>Indel: QD <2.0, FS >200.0, ReadPosRankSum <-20.0***<br>No recalibration	No filtered and recalibrated VCF file available	No filtered and recalibrated VCF file available	No filtered and recalibrated VCF file available

*Information on bioinformatics pipelines of vendors V1-V4 are according to supplied documentation and information stored in provided filtered VCF files. Note that for the different analysis steps, quality and quantity of information on both data analysis pipeline and platform metrics differed among vendors. Most information on data analysis workflow was provided by V1 and V2, whereas information from V3 was only obtained upon request and the report of V4 was rather poor.
**Marked duplicates were removed from the provided/generated BAM files using Picard v1.108/1.118 MarkDuplicates.
***Nomenclature according to VCF v4.1 format.
****V4 defined on-target reads as reads within platforms' designed target region and 100-bp flanking sequences, number of aligned reads is given as total number of mapped unique reads without restriction on on-target reads (cf. Figure 1A, Supplementary Figure S13, and Supplementary Tables S5 and S24).
BWA, Burrows-Wheeler Aligner; CASAVA, Consensus Assessment of Sequence and Variation; GATK, Genome Analysis Tool Kit; RTA, real time analysis; 1000 GP, 1000 genome project; ESP, exome sequencing project; HGMD, human genome mutation database; ---, not performed; NA, not available.

Table S4. Enrichment efficiency for the designed target regions of the three enrichment platforms performed by the same vendor (V1).

**Agilent_V1_designed target region**	**44**	**280**	**326**	**2905**	**7344**	**7739**	**Mean**
Total number of designed targets	286'754	286'754	286'754	286'754	286'754	286'754	**286'754**
Total number of aligned reads	110'584'430	119'682'978	87'403'593	96'387'713	133'734'129	118'295'260	**111'014'684**
Approximate mean number of reads per designed target	270	289	211	233	322	290	**269**
Mean read depth	101	107	79	87	120	109	**100**
Mean % coverage at 1×	99.69	99.67	99.55	99.77	99.71	99.65	**99.67**
Mean % coverage at 20×	95.63	96.36	93.43	94.28	96.78	96.00	**95.41**
Approximate number of reads on target*	77'352'358	82'771'475	60'593'256	66'819'600	92'345'161	83'213'677	**77'182'588**
Approximate % of reads on target*	69.95	69.16	69.33	69.32	69.05	70.34	**69.52**
Approximate number of reads on target* ±500 bp	94'568'991	102'773'417	75'321'697	82'878'078	114'881'869	101'429'148	**95'308'866**
Approximate % of reads off target**	14.48	14.13	13.82	14.02	14.10	14.26	**14.15**
**NimbleGen_V1_designed target region**	44	280	326	2905	7344	7739	**Mean**
Total number of designed targets	237'172	237'172	237'172	237'172	237'172	237'172	**237'172**
Total number of aligned reads	186'538'676	129'934'094	122'855'749	151'654'787	143'047'403	159'150'546	**148'863'543**
Approximate mean number of reads per designed target	474	326	322	377	352	425	**379**
Mean read depth	106	71	71	84	78	96	**84**
Mean % coverage at 1×	91.30	86.62	89.31	90.29	90.93	90.98	**89.91**
Mean % coverage at 20×	81.73	73.46	77.14	79.15	79.72	80.53	**78.62**
Approximate number of reads on target*	112'460'374	77'223'935	76'439'364	89'323'164	83'535'838	100'795'033	**89'962'951**
Approximate % of reads on target*	60.29	59.43	62.22	58.90	58.40	63.33	**60.43**
Approximate number of reads on target* ±500 bp	141'280'259	98'855'834	97'276'602	113'270'989	105'729'484	125'022'431	**113'572'600**
Approximate % of reads off target**	24.26	23.92	20.82	25.31	26.09	21.44	**23.71**
**Illumina_V1_designed target region**	44	280	326	2905	7344	7739	**Mean**
Total number of designed targets	201'071	201'071	201'071	201'071	201'071	201'071	**201'071**
Total number of aligned reads	88'290'788	95'545'396	79'962'090	109'038'759	101'342'654	72'695'510	**91'145'866**
Approximate mean number of reads per designed target	197	218	180	242	223	165	**204**
Mean read depth	80	85	74	96	87	67	**81**
Mean % coverage at 1×	99.36	99.32	99.19	99.57	99.47	99.18	**99.35**
Mean % coverage at 20×	89.96	91.34	88.57	92.72	91.87	86.99	**90.24**
Approximate number of reads on target*	39'675'247	43'734'230	36'185'233	48'569'940	44'852'503	33'136'433	**41'025'598**
Approximate % of reads on target*	44.94	45.77	45.25	44.54	44.26	45.58	**45.01**
Approximate number of reads on target* ±500 bp	52'331'403	58'686'892	46'659'126	64'833'841	60'567'552	43'352'624	**54'405'240**
Approximate % of reads off target**	40.73	38.58	41.65	40.54	40.23	40.36	**40.31**

*Only reads within designed target region are considered as on target; **Off-target enrichment was assessed as a fraction of total aligned reads without duplicates which mapped more than 500 bp outside the designed target regions.

Table S5. Enrichment efficiency for the designed target regions of the three enrichment platforms performed by different vendors (V2-V4).

**Agilent_V2_designed target region**	**44**	**280**	**326**	**2905**	**7344**	**7739**	**Mean**
Total number of designed targets	286'754	286'754	286'754	286'754	286'754	286'754	**286'754**
Total number of aligned reads	172'118'729	160'900'330	157'585'695	129'503'393	156'320'331	156'695'689	**155'520'695**
Approximate mean number of reads per designed target	393	367	356	291	350	359	**353**
Mean read depth	148	137	133	108	131	134	**132**
Mean % coverage at 1×	99.73	99.72	99.77	99.86	99.72	99.73	**99.75**
Mean % coverage at 20×	97.97	97.79	97.81	96.78	97.61	97.67	**97.60**
Approximate number of reads on target*	112'787'851	105'277'451	102'187'865	83'425'965	100'486'466	102'954'139	**101'186'623**
Approximate % of reads on target*	65.53	65.43	64.85	64.42	64.28	65.70	**65.06**
Approximate number of reads on target* ±500 bp	140'118'470	133'378'927	129'248'040	106'320'714	128'338'725	128'600'703	**127'667'597**
Approximate % of reads off target**	18.59	17.10	17.98	17.90	17.90	17.93	**17.91**
**NimbleGen_V3_designed target region**	44	280	326	2905	7344	7739	**Mean**
Total number of designed targets	237'172	237'172	237'172	237'172	237'172	237'172	**237'172**
Total number of aligned reads***	72'899'287	158'324'581	61'382'269	90'817'153	121'277'235	41'985'030	**91'114'259**
Approximate mean number of reads per designed target	203	446	172	250	334	115	**253**
Mean read depth	46	94	39	55	72	26	**55**
Mean % coverage at 1×	98.56	98.70	98.48	98.74	98.70	98.36	**98.59**
Mean % coverage at 20×	92.12	95.51	89.17	91.48	94.60	71.83	**89.12**
Approximate number of reads on target*	48'213'734	105'669'383	40'763'348	59'221'672	79'236'343	27'159'248	**60'043'955**
Approximate % of reads on target*	66.14	66.74	66.41	65.21	65.33	64.69	**65.90**
Approximate number of reads on target* ±500 bp	64'166'634	139'984'744	54'364'051	79'815'713	106'988'793	37'100'565	**80'403'417**
Approximate % of reads off target**	11.98	11.58	11.43	12.11	11.78	11.63	**11.76**
**Illumina_V4_designed target region**	44	280	326	2905	7344	7739	**Mean**
Total number of designed targets	201'071	201'071	201'071	201'071	201'071	201'071	**201'071**
Total number of aligned reads***	53'245'236	45'562'196	56'364'527	57'138'610	55'996'771	47'498'550	**52'634'315**
Approximate mean number of reads per designed target	137	114	140	143	139	121	**132**
Mean read depth	54	46	57	57	54	48	**53**
Mean % coverage at 1×	98.49	98.32	98.50	98.63	98.58	98.41	**98.49**
Mean % coverage at 20×	85.85	81.45	86.24	86.00	82.44	82.44	**84.07**
Approximate number of reads on target*	27'610'149	22'994'522	28'087'064	28'707'234	27'898'446	24'301'298	**26'599'785**
Approximate % of reads on target*	51.85	50.47	49.83	50.24	49.82	51.16	**50.54**
Approximate number of reads on target* ±500 bp	33'196'467	27'625'088	33'570'343	34'504'989	33'608'122	29'249'735	**31'959'124**
Approximate % of reads off target**	37.65	39.37	40.44	39.61	39.98	38.42	**39.28**

*Only reads within designed target region are considered as on target; **Off-target enrichment was assessed as a fraction of total aligned reads without duplicates which mapped more than 500 bp outside the designed target regions; ***only unique reads (cf. Supplementary Table S3).

Table S6. Enrichment efficiency of the three enrichment platforms performed by the same vendor (V1) for RefSeq exons.

**Agilent_V1_RefSeq_all**	**44**	**280**	**326**	**2905**	**7344**	**7739**	**Mean**
Total number of exons	232'619	232'619	232'619	232'619	232'619	232'619	**232'619**
Approximate number of reads on exons	62'700'606	67'193'698	49'197'375	54'225'588	75'061'028	67'190'204	**62'594'750**
Approximate mean number of reads per exon	270	289	211	233	323	289	**269**
Mean read depth	90	95	70	77	106	96	**89**
Mean % coverage at 1×	91.18	91.35	90.47	90.96	91.49	91.19	**91.11**
Mean % coverage at 20×	83.57	84.27	81.67	82.43	84.74	83.79	**83.41**
**NimbleGen_V1_RefSeq_all**	**44**	**280**	**326**	**2905**	**7344**	**7739**	**Mean**
Total number of exons	232'619	232'619	232'619	232'619	232'619	232'619	**232'619**
Approximate number of reads on exons	88'680'868	60'566'348	60'014'994	70'443'258	65'994'615	79'544'377	**70'874'077**
Approximate mean number of reads per exon	381	260	258	303	284	342	**305**
Mean read depth	111	73	74	87	82	101	**88**
Mean % coverage at 1×	88.92	83.36	86.01	87.67	88.40	88.15	**87.09**
Mean % coverage at 20×	77.45	68.82	72.83	75.00	75.72	76.31	**74.36**
**Illumina_V1_RefSeq_all**	**44**	**280**	**326**	**2905**	**7344**	**7739**	**Mean**
Total number of exons	232'619	232'619	232'619	232'619	232'619	232'619	**232'619**
Approximate number of reads on exons	43'672'521	48'168'416	39'641'712	53'453'760	49'393'423	36'401'395	**45'121'871**
Approximate mean number of reads per exon	188	207	170	230	212	156	**194**
Mean read depth	73	78	68	88	80	61	**75**
Mean % coverage at 1×	94.40	94.43	93.85	95.34	95.11	93.62	**94.46**
Mean % coverage at 20×	81.61	82.94	80.23	84.31	83.48	78.81	**81.90**
**Agilent_V1_RefSeq_coding region**	**44**	**280**	**326**	**2905**	**7344**	**7739**	**Mean**
Total number of exons	202'044	202'044	202'044	202'044	202'044	202'044	**202'044**
Approximate number of reads on exons	39'019'740	41'231'642	30'210'489	33'413'675	46'161'327	41'534'098	**38'595'162**
Approximate mean number of reads per exon	193	204	150	165	228	206	**191**
Mean read depth	106	113	82	91	126	114	**105**
Mean % coverage at 1×	98.88	98.89	98.74	98.93	98.93	98.86	**98.87**
Mean % coverage at 15×	96.34	96.70	95.22	95.73	96.94	96.41	**96.22**
Mean % coverage at 20×	94.89	95.52	92.97	93.72	95.96	95.13	**94.70**
**NimbleGen_V1_RefSeq_coding region**	**44**	**280**	**326**	**2905**	**7344**	**7739**	**Mean**
Total number of exons	202'044	202'044	202'044	202'044	202'044	202'044	**202'044**
Approximate number of reads on exons	44'148'941	28'580'556	28'882'087	34'833'826	32'716'041	40'243'688	**34'900'857**
Approximate mean number of reads per exon	219	141	143	172	162	199	**173**
Mean read depth	118	77	78	92	87	108	**93**
Mean % coverage at 1×	91.66	86.20	89.39	90.39	91.22	91.35	**90.04**
Mean % coverage at 15×	83.76	74.96	79.37	81.45	82.41	82.77	**80.79**
Mean % coverage at 20×	82.23	73.03	77.42	79.64	80.55	81.11	**79.00**
**Illumina_V1_RefSeq_coding region**	**44**	**280**	**326**	**2905**	**7344**	**7739**	**Mean**
Total number of exons	202'044	202'044	202'044	202'044	202'044	202'044	**202'044**
Approximate number of reads on exons	25'832'596	28'353'998	23'513'955	31'645'766	29'003'715	21'497'098	**26'641'188**
Approximate mean number of reads per exon	128	140	116	157	144	106	**132**
Mean read depth	77	83	72	93	84	64	**79**
Mean % coverage at 1×	97.64	97.64	97.38	98.10	97.94	97.30	**97.67**
Mean % coverage at 15×	90.05	91.13	88.85	92.05	91.57	88.05	**90.28**
Mean % coverage at 20×	86.62	88.18	85.31	89.47	88.61	83.75	**86.99**
**Agilent_V1_RefSeq_5'UTR**	**44**	**280**	**326**	**2905**	**7344**	**7739**	**Mean**
Total number of exons	54'367	54'367	54'367	54'367	54'367	54'367	**54'367**
Approximate number of reads on exons	6'377'126	6'675'046	4'884'380	5'431'411	7'389'371	6'520'103	**6'212'906**
Approximate mean number of reads per exon	117	123	90	100	136	120	**114**
Mean read depth	75	79	57	64	88	78	**74**
Mean % coverage at 1×	76.09	76.42	73.59	75.17	76.82	75.95	**75.67**
Mean % coverage at 20×	59.76	60.43	57.51	58.69	60.80	59.30	**59.41**
**NimbleGen_V1_RefSeq_5'UTR**	**44**	**280**	**326**	**2905**	**7344**	**7739**	**Mean**
Total number of exons	54'367	54'367	54'367	54'367	54'367	54'367	**54'367**
Approximate number of reads on exons	8'353'576	5'446'837	5'498'776	6'667'355	6'228'849	7'488'157	**6'613'925**
Approximate mean number of reads per exon	154	100	101	123	115	138	**122**
Mean read depth	77	50	51	61	58	70	**61**
Mean % coverage at 1×	72.68	65.89	68.41	71.05	71.97	71.52	**70.25**
Mean % coverage at 20×	56.37	48.31	51.72	54.04	54.53	55.22	**53.37**
**Illumina_V1_RefSeq_5'UTR**	**44**	**280**	**326**	**2905**	**7344**	**7739**	**Mean**
Total number of exons	54'367	54'367	54'367	54'367	54'367	54'367	**54'367**
Approximate number of reads on exons	5'909'735	6'236'696	5'328'908	7'026'514	6'536'708	4'863'018	**5'983'597**
Approximate mean number of reads per exon	109	115	98	129	120	89	**110**
Mean read depth	58	60	54	68	63	48	**59**
Mean % coverage at 1×	86.76	86.66	85.53	88.56	88.26	85.03	**86.80**
Mean % coverage at 20×	64.53	65.10	62.36	67.45	66.56	61.05	**64.51**
**Agilent_V1_RefSeq_3'UTR**	**44**	**280**	**326**	**2905**	**7344**	**7739**	**Mean**
Total number of exons	38'600	38'600	38'600	38'600	38'600	38'600	**38'600**
Approximate number of reads on exons	22'781'870	25'188'734	18'416'436	20'179'031	28'116'725	25'049'443	**23'288'706**
Approximate mean number of reads per exon	590	653	477	523	728	649	**603**
Mean read depth	76	82	60	66	92	82	**76**
Mean % coverage at 1×	82.58	83.15	81.69	82.32	83.36	82.82	**82.65**
Mean % coverage at 20×	70.26	71.20	68.70	69.37	71.76	70.56	**70.31**
**NimbleGen_V1_RefSeq_3'UTR**	**44**	**280**	**326**	**2905**	**7344**	**7739**	**Mean**
Total number of exons	38'600	38'600	38'600	38'600	38'600	38'600	**38'600**
Approximate number of reads on exons	43'473'480	31'653'358	30'648'749	34'766'931	32'483'625	38'331'765	**35'226'318**
Approximate mean number of reads per exon	1126	820	794	901	842	993	**913**
Mean read depth	116	80	79	91	86	103	**92**
Mean % coverage at 1×	90.39	85.73	86.86	89.62	89.80	88.68	**88.51**
Mean % coverage at 20×	75.98	69.25	72.10	74.00	74.00	74.77	**73.35**
**Illumina_V1_RefSeq_3'UTR**	**44**	**280**	**326**	**2905**	**7344**	**7739**	**Mean**
Total number of exons	38'600	38'600	38'600	38'600	38'600	38'600	**38'600**
Approximate number of reads on exons	14'879'463	16'813'223	13'545'316	18'384'906	17'152'881	12'513'243	**15'548'172**
Approximate mean number of reads per exon	385	436	351	476	444	324	**403**
Mean read depth	51	57	47	63	58	43	**53**
Mean % coverage at 1×	88.02	88.35	86.51	90.25	89.79	86.13	**88.17**
Mean % coverage at 20×	63.36	66.11	60.35	67.72	66.96	59.38	**63.98**

Table S7. Enrichment efficiency of the three enrichment platforms performed by the same vendor (V1) for intronic sequences (regions) in our region of interest (RefSeq coding exons and -50-bp and +20-bp flanking intronic sequences).

**Agilent_V1_RefSeq_coding_-50 bp**	**44**	**280**	**326**	**2905**	**7344**	**7739**	**Mean**
Total number of regions	199'107	199'107	199'107	199'107	199'107	199'107	**199'107**
Approximate number of reads on regions	7'920'308	8'502'572	6'214'050	6'888'265	9'510'776	8'449'249	**7'914'203**
Approximate mean number of reads per region	40	43	31	35	48	42	**40**
Mean read depth	80	85	62	69	96	85	**79**
Mean % coverage at 15×	91.30	92.98	89.04	90.12	93.85	91.69	**91.50**
Mean % coverage at 20×	87.17	89.56	83.61	85.22	90.96	87.95	**87.41**
**NimbleGen_V1_RefSeq_coding_-50 bp**	**44**	**280**	**326**	**2905**	**7344**	**7739**	**Mean**
Total number of regions	199'107	199'107	199'107	199'107	199'107	199'107	**199'107**
Approximate number of reads on regions	9'159'017	6'170'791	6'173'059	7'270'276	6'809'552	8'260'991	**7'307'281**
Approximate mean number of reads per region	46	31	31	37	34	41	**37**
Mean read depth	92	62	62	73	68	83	**73**
Mean % coverage at 15×	83.22	75.16	78.93	80.70	81.39	82.00	**80.23**
Mean % coverage at 20×	81.15	72.49	76.15	78.06	78.56	79.63	**77.67**
**Illumina_V1_RefSeq_coding_-50 bp**	**44**	**280**	**326**	**2905**	**7344**	**7739**	**Mean**
Total number of regions	199'107	199'107	199'107	199'107	199'107	199'107	**199'107**
Approximate number of reads on regions	3'853'422	4'356'279	3'427'392	4'798'041	4'455'867	3'175'805	**4'011'134**
Approximate mean number of reads per region	19	22	17	24	22	16	**20**
Mean read depth	39	44	34	48	45	32	**40**
Mean % coverage at 15×	69.47	74.60	64.63	76.83	75.49	63.12	**70.69**
Mean % coverage at 20×	59.39	65.20	54.87	68.03	66.04	52.51	**61.01**
**Agilent_V1_RefSeq_coding_+20 bp**	**44**	**280**	**326**	**2905**	**7344**	**7739**	**Mean**
Total number of regions	197'564	197'564	197'564	197'564	197'564	197'564	**197'564**
Approximate number of reads on regions	3'518'506	3'754'173	2'751'585	3'037'132	4'200'888	3'771'193	**3'505'579**
Approximate mean number of reads per region	18	19	14	15	21	19	**18**
Mean read depth	89	95	70	77	106	95	**89**
Mean % coverage at 15×	93.40	94.42	91.40	92.21	95.12	93.56	**93.35**
Mean % coverage at 20×	90.15	91.73	86.87	88.15	92.88	90.62	**90.07**
**NimbleGen_V1_RefSeq_coding_+20 bp**	**44**	**280**	**326**	**2905**	**7344**	**7739**	**Mean**
Total number of regions	197'564	197'564	197'564	197'564	197'564	197'564	**197'564**
Approximate number of reads on regions	4'278'498	2'817'235	2'843'002	3'359'239	3'161'171	3'852'469	**3'385'269**
Approximate mean number of reads per region	22	14	14	17	16	19	**17**
Mean read depth	108	71	72	85	80	97	**86**
Mean % coverage at 15×	83.23	74.56	78.73	80.88	81.70	82.13	**80.21**
Mean % coverage at 20×	81.51	72.47	76.56	78.81	79.55	80.24	**78.19**
**Illumina_V1_RefSeq_coding_+20 bp**	**44**	**280**	**326**	**2905**	**7344**	**7739**	**Mean**
Total number of regions	197'564	197'564	197'564	197'564	197'564	197'564	**197'564**
Approximate number of reads on regions	1'766'131	1'992'962	1'992'962	2'203'580	2'039'491	1'459'744	**1'909'145**
Approximate mean number of reads per region	9	10	10	11	10	7	**10**
Mean read depth	45	50	50	56	52	37	**48**
Mean % coverage at 15×	76.66	80.59	73.27	82.81	81.58	71.35	**77.71**
Mean % coverage at 20×	67.63	72.55	64.19	75.46	73.50	61.19	**69.09**

Table S8. Uniformity of the coverage of RefSeq coding exons.

**Agilent_V1_RefSeq_coding region_uniformity**	**44**	**280**	**326**	**2905**	**7344**	**7739**	**Mean**
Total number of exons	202'044	202'044	202'044	202'044	202'044	202'044	**202'044**
Mean read depth	106	113	82	91	126	114	**105**
Exons with 100% coverage at 20×	175'281	179'977	166'032	169'766	183'167	176'838	**175'177**
Exons with 100% coverage at 20× [%]	86.75	89.08	82.18	84.02	90.66	87.52	**86.70**
Exons with average read depth within ±70% of mean read depth	160'167	161'721	161'019	159'423	160'356	160'068	**160'459**
Exons with average read depth within ±70% of mean read depth [%]	79.27	80.04	79.70	78.91	79.37	79.22	**79.42**
**NimbleGen_V1_RefSeq_coding region_uniformity**	**44**	**280**	**326**	**2905**	**7344**	**7739**	**Mean**
Total number of exons	202'044	202'044	202'044	202'044	202'044	202'044	**202'044**
Mean read depth	118	77	78	92	87	108	**93**
Exons with 100% coverage at 20×	153'113	134'901	142'875	147'416	149'468	149'806	**146'263**
Exons with 100% coverage at 20× [%]	75.78	66.77	70.71	72.96	73.98	74.15	**72.39**
Exons with average read depth within ±70% of mean read depth	131'419	111'898	125'842	126'789	131'882	129'236	**126'178**
Exons with average read depth within ±70% of mean read depth [%]	65.04	55.38	62.28	62.75	65.27	63.96	**62.45**
**Illumina_V1_RefSeq_coding region_uniformity**	**44**	**280**	**326**	**2905**	**7344**	**7739**	**Mean**
Total number of exons	202'044	202'044	202'044	202'044	202'044	202'044	**202'044**
Mean read depth	77	83	72	93	84	64	**79**
Exons with 100% coverage at 20×	124'912	134'570	117'424	141'082	136'283	111'031	**127'550**
Exons with 100% coverage at 20× [%]	61.82	66.60	58.12	69.83	67.45	54.95	**63.13**
Exons with average read depth within ±70% of mean read depth	150'075	149'421	154'065	149'234	149'318	151'963	**150'679**
Exons with average read depth within ±70% of mean read depth [%]	74.28	73.95	76.25	73.86	73.90	75.21	**74.58**
**Agilent_V2_RefSeq_coding region_uniformity**	**44**	**280**	**326**	**2905**	**7344**	**7739**	**Mean**
Total number of exons	202'044	202'044	202'044	202'044	202'044	202'044	**202'044**
Mean read depth	156	144	140	114	137	142	**139**
Exons with 100% coverage at 20×	188'839	188'779	188'675	183'648	188'298	187'339	**187'596**
Exons with 100% coverage at 20× [%]	93.46	93.43	93.38	90.90	93.20	92.72	**92.85**
Exons with average read depth within ±70% of mean read depth	161'636	162'627	162'929	161'077	162'061	161'861	**162'032**
Exons with average read depth within ±70% of mean read depth [%]	80.00	80.49	80.64	79.72	80.21	80.11	**80.20**
**NimbleGen_V3_RefSeq_coding region_uniformity**	**44**	**280**	**326**	**2905**	**7344**	**7739**	**Mean**
Total number of exons	202'044	202'044	202'044	202'044	202'044	202'044	**202'044**
Mean read depth	51	104	42	59	79	28	**61**
Exons with 100% coverage at 20×	184'605	193'678	175'023	181'855	191'318	114'754	**173'539**
Exons with 100% coverage at 20× [%]	91.37	95.86	86.63	90.01	94.69	56.80	**85.89**
Exons with average read depth within ±70% of mean read depth	192'342	182'511	192'255	185'321	187'782	190'225	**188'406**
Exons with average read depth within ±70% of mean read depth [%]	95.20	90.33	95.16	91.72	92.94	94.15	**93.25**
**Illumina_V4_RefSeq_coding region_uniformity**	**44**	**280**	**326**	**2905**	**7344**	**7739**	**Mean**
Total number of exons	202'044	202'044	202'044	202'044	202'044	202'044	**202'044**
Mean read depth	53	45	55	55	52	46	**51**
Exons with 100% coverage at 20×	112'625	95'971	112'640	114'902	113'604	100'317	**108'343**
Exons with 100% coverage at 20× [%]	55.74	47.50	55.75	56.87	56.23	49.65	**53.62**
Exons with average read depth within ±70% of mean read depth	152'575	154'422	153'579	152'341	152'382	152'967	**153'044**
Exons with average read depth within ±70% of mean read depth [%]	75.52	76.43	76.01	75.40	75.42	75.71	**75.75**

Table S9. Coverage of RefSeq coding exons by whole genome sequencing (WGS).

WGS_RefSeq_coding region	7739_V1 (60×)	7344_V3 (30×)	7739_V3 (30×)	7739_V4 (30×)	7739_V4_X Ten (60×)
Total number of exons	202'044	202'044	202'044	202'044	202'044
Approximate number of reads on exons	11'674'285	5'461'115	5'113'945	7'708'299	22'846'600
Approximate mean number of reads per exon	58	27	25	38	75
Mean read depth	33	16	15	22	65
Mean % coverage at 10×	99.63	91.60	88.56	98.37	99.02
Mean % coverage at 20×	97.57	21.49	14.83	64.21	98.68
Exons with average read depth within ±70% of mean read depth [%]	99.33	97.54	97.27	98.84	98.14
Exons with <100% coverage at 20× [%]	12.55	96.92	98.21	69.81	1.84
Exons with <100% coverage at 15× [%]	2.65	79.05	85.42	37.26	1.55
Exons with <100% coverage at 10× [%]	0.74	32.17	40.45	9.12	1.31

X Ten, HiSeq X Ten system.

Table S10. Proportions of RefSeq coding exons not completely (100%) covered at 20× neither by using the three platforms alone nor in any combination. In the last row, the proportions of exons completely covered by all six platform-vendor combinations (shared exons covered at 20×) are given in italics as well. If not otherwise indicated, data of all corresponding vendors are included.

Platform(s)	44	280	326	2905	7344	7739	Mean*
Agilent (vendor V1)	13.25%	10.92%	17.82%	15.98%	9.34%	12.48%	**13.30±3.31%**
NimbleGen (vendor V1)	24.22%	33.23%	29.29%	27.04%	26.02%	25.85%	**27.61±3.38%**
Illumina (vendor V1)	38.18%	33.40%	41.88%	30.17%	32.55%	45.05%	**36.87±6.11%**
Agilent (vendor V2)	6.54%	6.57%	6.62%	9.10%	6.80%	7.28%	**7.15±1.05%**
NimbleGen (vendor V3)	8.63%	4.14%	13.37%	9.99%	5.31%	43.20%	**14.11±15.36%**
Illumina (vendor V4)	44.26%	52.50%	44.25%	43.13%	43.77%	50.35%	**46.38±4.19%**
Agilent (vendors V1 and V2)	6.36%	6.21%	6.54%	8.51%	6.19%	6.87%	**6.78±0.93%**
NimbleGen (vendors V1 and V3)	6.33%	3.25%	10.24%	7.92%	4.19%	20.74%	**8.78±6.7%**
Illumina (vendors V1 and V4)	31.20%	29.90%	33.34%	25.96%	27.38%	37.12%	**30.82±4.27%**
Agilent and NimbleGen	2.08%	1.78%	2.49%	2.41%	1.98%	3.19%	**2.32±0.53%**
Agilent and Illumina	2.95%	2.83%	3.10%	3.32%	2.63%	3.48%	**3.05±0.33%**
NimbleGen and Illumina	3.45%	2.13%	4.91%	3.47%	2.48%	8.37%	**4.13±2.40%**
All three platforms	1.58%	1.39%	1.78%	1.55%	1.50%	2.11%	**1.65±0.27%**
Shared exons covered at 20× [%]	*32.24%*	*25.60%*	*27.68%*	*33.01%*	*34.15%*	*19.65%*	***28.72±5.81%***

*Indicated ranges for mean values (±) represent 95% confidence intervals.

Table S11. Coverage of RefSeq coding exons of ~7600 genes targeted by the Accuracy and Content Enhanced (ACEv2) clinical exome platform of Personalis (www.personalis.com) achieved using WES at 100×, WGS at 30× and 60×, and Personalis ACEv2 at 60×.

	Total exons	Mean read depth	Mean coverage at 20× [%]	Exons with <100% coverage at 20× [%]	Exons with <100% coverage at 15× [%]	Exons with <100% coverage at 10× [%]
7739_Agilent_V1	99'236	115	95.98	11.50	7.50	4.36
7739_Agilent_V2	99'236	143	97.41	6.31	4.10	2.55
7739_NimbleGen_V1	99'236	109	82.59	24.26	22.35	20.33
7739_NimbleGen_V3	99'236	28	81.37	41.69	20.51	8.29
7739_Illumina_V1	99'236	63	83.79	46.07	35.60	23.76
7739_Illumina_V4	99'236	47	81.79	47.52	34.74	21.62
7739_WGS_V3_30×	99'236	15	15.47	97.98	85.04	39.23
7739_WGS_V4_30×	99'236	22	65.18	69.35	36.09	8.55
7739_WGS_V1_60×	99'236	34	97.72	11.83	2.39	0.71
7739_WGS_XTen_V4_60× (non-PCR-free)	99'236	66	98.91	1.50	1.26	1.08
7739_Personalis	99'236	68	96.75	12.54	5.48	1.73
374_Personalis (additional DNA 1)	99'236	72	97.44	9.83	4.02	1.21
7498_Personalis (additional DNA 2)	99'236	68	96.53	12.39	5.32	1.56

Table S12. Enrichment efficiency of the three enrichment platforms performed by different vendors (V2-V4) for RefSeq exons.

Agilent_V2_RefSeq_all	44	280	326	2905	7344	7739	Mean
Total number of exons	232'619	232'619	232'619	232'619	232'619	232'619	**232'619**
Approximate number of reads on exons	91'956'375	85'988'017	83'572'762	68'364'391	82'213'511	83'948'932	**82'673'998**
Approximate mean number of reads per exon	395	370	359	294	353	361	**355**
Mean read depth	132	121	118	96	116	120	**117**
Mean % coverage at 1×	95.15	94.48	94.72	94.57	94.78	94.63	**94.72**
Mean % coverage at 20×	85.93	85.79	85.77	84.83	85.65	85.58	**85.59**
**NimbleGen_V3_RefSeq_all**	**44**	**280**	**326**	**2905**	**7344**	**7739**	**Mean**
Total number of exons	232'619	232'619	232'619	232'619	232'619	232'619	**232'619**
Approximate number of reads on exons	37'142'009	83'642'818	31'319'114	45'838'652	61'584'881	20'887'479	**46'735'825**
Approximate mean number of reads per exon	160	360	135	197	265	90	**201**
Mean read depth	48	98	40	56	74	26	**57**
Mean % coverage at 1×	95.53	96.10	95.26	95.81	96.00	94.99	**95.61**
Mean % coverage at 20×	89.58	92.04	87.36	88.74	91.25	73.80	**87.13**
**Illumina_V4_RefSeq_all**	**44**	**280**	**326**	**2905**	**7344**	**7739**	**Mean**
Total number of exons	232'619	232'619	232'619	232'619	232'619	232'619	**232'619**
Approximate number of reads on exons	29'121'995	24'223'385	29'600'776	30'262'529	29'426'507	25'627'386	**28'043'763**
Approximate mean number of reads per exon	125	104	127	130	127	110	**121**
Mean read depth	48	41	50	51	48	42	**47**
Mean % coverage at 1×	88.10	87.93	88.11	88.22	88.18	88.02	**88.09**
Mean % coverage at 20×	76.25	72.23	76.57	76.74	76.39	73.18	**75.23**
**Agilent_V2_RefSeq_coding region**	**44**	**280**	**326**	**2905**	**7344**	**7739**	**Mean**
Total number of exons	202'044	202'044	202'044	202'044	202'044	202'044	**202'044**
Approximate number of reads on exons	57'570'213	52'867'135	51'777'832	42'473'247	50'455'925	52'303'873	**51'241'371**
Approximate mean number of reads per exon	285	262	256	210	250	259	**254**
Mean read depth	156	144	140	114	137	142	**139**
Mean % coverage at 1×	99.19	99.13	99.19	99.26	99.16	99.14	**99.18**
Mean % coverage at 15×	97.59	97.49	97.49	97.07	97.42	97.42	**97.42**
Mean % coverage at 20×	96.96	96.82	96.81	95.92	96.69	96.67	**96.65**
**NimbleGen_V3_RefSeq_coding region**	**44**	**280**	**326**	**2905**	**7344**	**7739**	**Mean**
Total number of exons	202'044	202'044	202'044	202'044	202'044	202'044	**202'044**
Approximate number of reads on exons	18'364'643	39'619'058	15'302'804	21'727'594	29'212'627	10'084'786	**22'385'252**
Approximate mean number of reads per exon	91	196	76	108	145	50	**111**
Mean read depth	51	104	42	59	79	28	**61**
Mean % coverage at 1×	98.63	98.72	98.56	98.76	98.75	98.52	**98.66**
Mean % coverage at 15×	96.71	97.65	95.78	96.26	97.34	89.99	**95.62**
Mean % coverage at 20×	95.39	97.14	93.37	94.51	96.61	79.76	**92.80**
**Illumina_V4_RefSeq_coding region**	**44**	**280**	**326**	**2905**	**7344**	**7739**	**Mean**
Total number of exons	202'044	202'044	202'044	202'044	202'044	202'044	**202'044**
Approximate number of reads on exons	18'228'955	15'256'346	18'765'557	19'068'256	18'356'073	15'984'498	**17'609'947**
Approximate mean number of reads per exon	90	76	93	94	91	79	**87**
Mean read depth	53	45	55	55	52	46	**51**
Mean % coverage at 1×	94.30	94.23	94.32	94.41	94.34	94.26	**94.31**
Mean % coverage at 15×	88.09	85.39	88.24	88.33	88.27	86.20	**87.42**
Mean % coverage at 20×	83.17	79.06	83.67	83.73	83.28	79.88	**82.13**
**Agilent_V2_RefSeq_5'UTR**	**44**	**280**	**326**	**2905**	**7344**	**7739**	**Mean**
Total number of exons	54'367	54'367	54'367	54'367	54'367	54'367	**54'367**
Approximate number of reads on exons	9'544'557	8'639'899	8'605'274	7'141'824	8'259'030	8'543'370	**8'455'659**
Approximate mean number of reads per exon	176	159	158	131	152	157	**156**
Mean read depth	113	102	101	83	97	101	**100**
Mean % coverage at 1×	88.33	86.25	86.87	86.31	87.06	86.78	**86.93**
Mean % coverage at 20×	62.99	62.54	62.80	61.97	62.30	62.34	**62.49**
**NimbleGen_V3_RefSeq_5'UTR**	**44**	**280**	**326**	**2905**	**7344**	**7739**	**Mean**
Total number of exons	54'367	54'367	54'367	54'367	54'367	54'367	**54'367**
Approximate number of reads on exons	4'208'508	8'995'809	3'490'069	4'840'571	6'547'724	2'248'176	**5'055'143**
Approximate mean number of reads per exon	77	165	64	89	120	41	**93**
Mean read depth	39	79	32	44	59	21	**46**
Mean % coverage at 1×	91.62	92.85	90.96	91.94	92.50	90.20	**91.68**
Mean % coverage at 20×	76.14	81.70	71.76	73.82	79.44	54.12	**72.83**
**Illumina_V4_RefSeq_5'UTR**	**44**	**280**	**326**	**2905**	**7344**	**7739**	**Mean**
Total number of exons	54'367	54'367	54'367	54'367	54'367	54'367	**54'367**
Approximate number of reads on exons	3'836'677	3'138'739	3'895'716	3'940'321	3'835'576	3'385'497	**3'672'088**
Approximate mean number of reads per exon	71	58	72	72	71	62	**68**
Mean read depth	39	32	40	40	39	34	**37**
Mean % coverage at 1×	75.70	75.33	75.72	75.75	75.81	75.59	**75.65**
Mean % coverage at 20×	59.32	54.96	59.24	59.55	59.51	56.63	**58.20**
**Agilent_V2_RefSeq_3'UTR**	**44**	**280**	**326**	**2905**	**7344**	**7739**	**Mean**
Total number of exons	38'600	38'600	38'600	38'600	38'600	38'600	**38'600**
Approximate number of reads on exons	32'871'014	32'041'868	30'475'740	24'734'719	30'719'883	30'428'127	**30'211'892**
Approximate mean number of reads per exon	852	830	790	641	796	788	**783**
Mean read depth	111	106	101	82	101	102	**100**
Mean % coverage at 1×	89.20	88.12	88.65	88.11	88.73	88.22	**88.51**
Mean % coverage at 20×	73.02	73.07	72.96	71.98	72.98	72.66	**72.78**
**NimbleGen_V3_RefSeq_3'UTR**	**44**	**280**	**326**	**2905**	**7344**	**7739**	**Mean**
Total number of exons	38'600	38'600	38'600	38'600	38'600	38'600	**38'600**
Approximate number of reads on exons	17'649'879	41'978'965	15'133'918	23'142'414	30'974'043	10'299'582	**23'196'467**
Approximate mean number of reads per exon	457	1088	392	600	802	267	**601**
Mean read depth	45	102	38	56	75	26	**57**
Mean % coverage at 1×	89.12	90.60	88.53	89.87	90.33	87.93	**89.39**
Mean % coverage at 20×	80.67	83.84	79.13	81.26	83.08	69.53	**79.59**
**Illumina_V4_RefSeq_3'UTR**	**44**	**280**	**326**	**2905**	**7344**	**7739**	**Mean**
Total number of exons	38'600	38'600	38'600	38'600	38'600	38'600	**38'600**
Approximate number of reads on exons	9'237'507	7'648'746	9'160'291	9'529'750	9'436'549	8'171'583	**8'864'071**
Approximate mean number of reads per exon	239	198	237	247	244	212	**230**
Mean read depth	31	26	31	32	31	27	**30**
Mean % coverage at 1×	75.45	74.74	75.34	75.63	75.72	75.16	**75.34**
Mean % coverage at 20×	54.27	48.44	53.00	54.55	55.19	50.81	**52.71**

Table S13. Enrichment efficiency of the three enrichment platforms performed by different vendors (V2-V4) for intronic sequences (regions) in our region of interest (RefSeq coding exons and -50-bp and +20-bp flanking intronic sequences).

Agilent_V2_RefSeq_coding_-50 bp	44	280	326	2905	7344	7739	Mean
Total number of regions	199'107	199'107	199'107	199'107	199'107	199'107	**199'107**
Approximate number of reads on regions	11'745'824	10'987'395	10'673'000	8'776'634	10'526'929	10'697'536	**10'567'886**
Approximate mean number of reads per region	59	55	54	44	53	54	**53**
Mean read depth	118	110	107	88	106	107	**106**
Mean % coverage at 15×	95.36	95.59	95.49	94.14	95.51	94.97	**95.18**
Mean % coverage at 20×	93.20	93.58	93.35	90.96	93.40	92.60	**92.85**
**NimbleGen_V3_RefSeq_coding_-50 bp**	**44**	**280**	**326**	**2905**	**7344**	**7739**	**Mean**
Total number of regions	199'107	199'107	199'107	199'107	199'107	199'107	**199'107**
Approximate number of reads on regions	4'193'288	8'643'131	3'535'739	4'957'626	6'606'299	2'341'553	**5'046'273**
Approximate mean number of reads per region	21	43	18	25	33	12	**25**
Mean read depth	42	87	36	50	66	24	**51**
Mean % coverage at 15×	95.04	97.23	93.01	94.58	96.68	81.11	**92.94**
Mean % coverage at 20×	91.34	96.36	86.93	91.25	95.34	64.00	**87.54**
**Illumina_V4_RefSeq_coding_-50 bp**	**44**	**280**	**326**	**2905**	**7344**	**7739**	**Mean**
Total number of regions	199'107	199'107	199'107	199'107	199'107	199'107	**199'107**
Approximate number of reads on regions	2'636'469	2'207'057	2'618'019	2'748'245	2'670'685	2'324'392	**2'534'145**
Approximate mean number of reads per region	13	11	13	14	13	12	**13**
Mean read depth	26	22	26	28	27	23	**25**
Mean % coverage at 15×	59.01	52.53	58.01	60.18	59.84	54.55	**57.35**
Mean % coverage at 20×	47.52	41.00	46.69	49.09	48.31	42.68	**45.88**
**Agilent_V2_RefSeq_coding_+20 bp**	**44**	**280**	**326**	**2905**	**7344**	**7739**	**Mean**
Total number of regions	197'564	197'564	197'564	197'564	197'564	197'564	**197'564**
Approximate number of reads on regions	5'151'344	4'799'004	4'665'424	3'820'170	4'585'481	4'691'331	**4'618'792**
Approximate mean number of reads per region	26	24	24	19	23	24	**23**
Mean read depth	130	121	118	97	116	119	**117**
Mean % coverage at 15×	96.39	96.42	96.39	95.43	96.31	96.11	**96.17**
Mean % coverage at 20×	94.90	94.89	94.84	93.11	94.70	94.36	**94.47**
**NimbleGen_V3_RefSeq_coding_+20 bp**	**44**	**280**	**326**	**2905**	**7344**	**7739**	**Mean**
Total number of regions	197'564	197'564	197'564	197'564	197'564	197'564	**197'564**
Approximate number of reads on regions	1'841'585	3'836'958	1'553'456	2'177'374	2'914'931	1'021'349	**2'224'276**
Approximate mean number of reads per region	9	19	8	11	15	5	**11**
Mean read depth	47	97	39	55	74	26	**56**
Mean % coverage at 15×	96.11	97.42	94.77	95.52	97.03	86.34	**94.53**
Mean % coverage at 20×	93.99	96.79	91.08	93.09	96.08	72.77	**90.63**
**Illumina_V4_RefSeq_coding_+20 bp**	**44**	**280**	**326**	**2905**	**7344**	**7739**	**Mean**
Total number of regions	197'564	197'564	197'564	197'564	197'564	197'564	**197'564**
Approximate number of reads on regions	1'270'409	1'059'550	1'275'194	1'325'726	1'287'699	1'119'616	**1'223'032**
Approximate mean number of reads per region	6	5	6	7	7	6	**6**
Mean read depth	32	27	32	34	33	28	**31**
Mean % coverage at 15×	72.12	65.46	71.51	72.77	72.80	68.02	**70.45**
Mean % coverage at 20×	61.06	53.39	60.71	62.41	61.81	55.83	**59.20**

Table S14. Enrichment efficiency of the three platforms performed by the same vendor (V1) for our panel of eight genes (exons including UTR).

**Agilent_V1_gene panel**	**44**	**280**	**326**	**2905**	**7344**	**7739**	**Mean**
Total number of exons	169	169	169	169	169	169	**169**
Approximate number of reads on exons	43'286	47'277	35'254	39'553	53'291	49'016	**44'612**
Approximate mean number of reads per exon	256	280	209	234	315	290	**264**
Mean read depth	95	103	76	85	117	108	**97**
Mean % coverage at 1×	97.54	97.54	97.43	97.62	97.93	98.28	**97.72**
Mean % coverage at 20×	93.85	95.29	91.75	93.18	94.96	94.43	**93.91**
**NimbleGen_V1_gene panel**	**44**	**280**	**326**	**2905**	**7344**	**7739**	**Mean**
Total number of exons	169	169	169	169	169	169	**169**
Approximate number of reads on exons	66'968	48'757	47'518	55'857	49'783	60'856	**54'956**
Approximate mean number of reads per exon	396	289	281	331	295	360	**325**
Mean read depth	133	92	91	110	99	124	**108**
Mean % coverage at 1×	96.36	94.48	96.11	96.51	96.42	96.09	**96.00**
Mean % coverage at 20×	92.48	85.32	88.16	90.46	91.05	91.32	**89.80**
**Illumina_V1_gene panel**	**44**	**280**	**326**	**2905**	**7344**	**7739**	**Mean**
Total number of exons	169	169	169	169	169	169	**169**
Approximate number of reads on exons	26'343	29'196	25'726	32'869	29'335	23'761	**27'872**
Approximate mean number of reads per exon	156	173	152	194	174	141	**165**
Mean read depth	73	80	74	90	79	66	**77**
Mean % coverage at 1×	98.63	98.87	98.61	98.91	98.91	98.80	**98.79**
Mean % coverage at 20×	89.48	91.22	88.69	89.90	91.03	87.13	**89.58**

Table S15. Enrichment efficiency of the three platforms performed by different vendors (V2-V4) for our panel of eight genes (exons including UTR).

**Agilent_V2_gene panel**	**44**	**280**	**326**	**2905**	**7344**	**7739**	**Mean**
Total number of exons	169	169	169	169	169	169	**169**
Approximate number of reads on exons	62'943	60'283	58'613	47'919	58'348	59'046	**57'859**
Approximate mean number of reads per exon	372	357	347	284	345	349	**342**
Mean read depth	140	130	128	105	127	130	**127**
Mean % coverage at 1×	98.81	99.35	99.07	98.40	99.23	98.91	**98.96**
Mean % coverage at 20×	95.77	96.54	95.98	95.12	96.38	95.82	**95.94**
**NimbleGen_V3_gene panel**	**44**	**280**	**326**	**2905**	**7344**	**7739**	**Mean**
Total number of exons	169	169	169	169	169	169	**169**
Approximate number of reads on exons	27'184	62'876	23'432	35'907	46'672	16'157	**35'371**
Approximate mean number of reads per exon	161	372	139	212	276	96	**209**
Mean read depth	53	115	45	66	86	31	**66**
Mean % coverage at 1×	99.99	100.00	100.00	100.00	100.00	99.79	**99.96**
Mean % coverage at 20×	96.42	98.53	96.31	96.51	98.63	88.86	**95.88**
**Illumina_V4_gene panel**	**44**	**280**	**326**	**2905**	**7344**	**7739**	**Mean**
Total number of exons	169	169	169	169	169	169	**169**
Approximate number of reads on exons	21'118	17'873	22'377	22'273	21'260	19'030	**20'655**
Approximate mean number of reads per exon	125	106	132	132	126	113	**122**
Mean read depth	59	51	64	63	59	52	**58**
Mean % coverage at 1×	98.93	98.73	98.77	98.71	98.81	98.52	**98.74**
Mean % coverage at 20×	90.59	89.01	91.84	93.09	91.21	89.76	**90.92**

Table S16. Number of heterozygous SNVs and indels identified by Sanger sequencing in the six DNA samples of this study. The number of homozygous SNVs is given in parentheses.

**Sample**	**SNVs**				**Indels**			
	**Total**	**ROI**	**Coding**	**UTR**	**Total**	**ROI**	**Coding**	**UTR**
44	16 (2)	4 (1)	1 (0)	2 (1)	3 (1)	0 (0)	0 (0)	0 (0)
280	10 (0)	4 (0)	0 (0)	1 (0)	3 (0)	1 (0)	1 (0)	0 (0)
326	1 (2)	1 (1)	0 (0)	0 (0)	1 (0)	1 (0)	1 (0)	0 (0)
2905	42 (2)	16 (2)	6 (0)	1 (0)	6 (0)	2 (0)	0 (0)	0 (0)
7344	39 (2)	18 (1)	8 (0)	1 (1)	6 (1)	3 (0)	1 (0)	0 (0)
7739	17 (3)	6 (0)	2 (0)	2 (0)	1 (0)	0 (0)	0 (0)	0 (0)
**Total**	**125 (11)**	**49 (5)**	**17 (0)**	**7 (2)**	**20 (2)**	**7 (0)**	**3 (0)**	**0 (0)**

ROI: region of interest (coding region with 50-bp upstream and 20-bp downstream intronic sequences).

Table S17. Efficiency and reproducibility of the three enrichment platforms for different heterozygous SNVs detected by Sanger sequencing. In the number pairs (m/n), the first number (m) represents number of different heterozygous SNVs obtained in at least one sample with sufficient number of reads (≥20). The second number (n) represents number of heterozygous positions detected by Sanger sequencing, which were identified in particular data sets with correct genotype and by at least one read.

**Total**																		
	**Total SNVs**						**SNVs in designed target region**						**SNVs in designed target region of all three platforms**					
Heterozygous	**AG (V1)**	**AG (V2)**	**NG (V1)**	**NG (V3)**	**ILL (V1)**	**ILL (V4)**	**AG (V1)**	**AG (V2)**	**NG (V1)**	**NG (V3)**	**ILL (V1)**	**ILL (V4)**	**AG (V1)**	**AG (V2)**	**NG (V1)**	**NG (V3)**	**ILL (V1)**	**ILL (V4)**
1/6 samples	30/44	36/43	39/42	38/44	28/42	21/32	21/26	24/26	30/30	30/31	9/9	8/9	8/8	8/8	8/8	8/8	8/8	7/8
2/6 samples	21/24	22/25	23/24	22/25	12/24	10/16	13/13	13/13	17/17	16/17	6/6	6/6	6/6	6/6	6/6	6/6	6/6	6/6
3/6 samples	7/7	7/7	7/7	5/6	1/5	1/5	3/3	3/3	6/6	5/6	1/1	1/1	1/1	1/1	1/1	1/1	1/1	1/1
4/6 samples	2/2	2/2	2/2	0/1	0/2	0	1/1	1/1	0	0	0	0	0	0	0	0	0	0
>1 sample	*30/33*	*31/34*	*32/33*	*27/32*	*13/31*	*11/21*	*17/17*	*17/17*	*23/23*	*21/23*	*7/7*	*7/7*	*7/7*	*7/7*	*7/7*	*7/7*	*7/7*	*7/7*
Positions with CR	2	1	1	4	2	5	0	0	0	2	0	0	0	0	0	0	0	0
True results	77	77	75	76	73	53	43	43	53	54	16	16	15	15	15	15	15	15
False and NA results	1	1	3	2	5	25	0	0	1	0	0	0	0	0	0	0	0	0
**Region of interest (ROI, coding region with 50-bp upstream and 20-bp downstream intronic sequences)**																		
	**Total SNVs**						**SNVs in designed target region**						**SNVs in designed target region of all three platforms**					
Heterozygous	**AG (V1)**	**AG (V2)**	**NG (V1)**	**NG (V3)**	**ILL (V1)**	**ILL (V4)**	**AG (V1)**	**AG (V2)**	**NG (V1)**	**NG (V3)**	**ILL (V1)**	**ILL (V4)**	**AG (V1)**	**AG (V2)**	**NG (V1)**	**NG (V3)**	**ILL (V1)**	**ILL (V4)**
1/6 samples	13/16	14/16	16/16	16/16	14/16	14/16	12/15	13/15	16/16	16/16	6/6	6/6	6/6	6/6	6/6	6/6	6/6	6/6
2/6 samples	9/9	9/9	9/9	8/9	7/9	8/9	9/9	9/9	9/9	8/9	4/4	4/4	4/4	4/4	4/4	4/4	4/4	4/4
3/6 samples	5/5	5/5	5/5	4/5	1/5	1/5	2/2	2/2	5/5	4/5	1/1	1/1	1/1	1/1	1/1	1/1	1/1	1/1
4/6 samples	0	0	0	0	0	0	0	0	0	0	0	0	0	0	0	0	0	0
>1 sample	14/14	14/14	14/14	12/14	8/14	9/14	11/11	11/11	14/14	12/14	5/5	5/5	5/5	5/5	5/5	5/5	5/5	5/5
Positions with CR	0	0	0	2	1	3	0	0	0	2	0	0	0	0	0	0	0	0
True results	30	30	30	30	30	30	26	26	30	30	11	11	11	11	11	11	11	11
False and NA results	0	0	0	0	0	0	0	0	0	0	0	0	0	0	0	0	0	0
**Untranslated regions (UTR)**																		
	**Total SNVs**						**SNVs in designed target region**						**SNVs in designed target region of all three platforms**					
Heterozygous	**AG (V1)**	**AG (V2)**	**NG (V1)**	**NG (V3)**	**ILL (V1)**	**ILL (V4)**	**AG (V1)**	**AG (V2)**	**NG (V1)**	**NG (V3)**	**ILL (V1)**	**ILL (V4)**	**AG (V1)**	**AG (V2)**	**NG (V1)**	**NG (V3)**	**ILL (V1)**	**ILL (V4)**
1/6 samples	2/3	2/3	2/2	2/3	3/3	2/3	2/2	2/2	2/2	2/3	3/3	2/3	2/2	2/2	2/2	2/2	2/2	1/2
2/6 samples	2/2	2/2	2/2	2/2	2/2	2/2	2/2	2/2	2/2	2/2	2/2	2/2	2/2	2/2	2/2	2/2	2/2	2/2
3/6 samples	0	0	0	0	0	0	0	0	0	0	0	0	0	0	0	0	0	0
4/6 samples	0	0	0	0	0	0	0	0	0	0	0	0	0	0	0	0	0	0
>1 sample	2/2	2/2	2/2	2/2	2/2	2/2	2/2	2/2	2/2	2/2	2/2	2/2	2/2	2/2	2/2	2/2	2/2	2/2
Positions with CR	0	0	0	0	0	0	0	0	0	0	0	0	0	0	0	0	0	0
True results	5	5	4	5	5	5	4	4	4	5	5	5	4	4	4	4	4	4
False and NA results	0	0	1	0	0	0	0	0	1	0	0	0	0	0	0	0	0	0

SNV: single nucleotide variant; AG: Agilent; NG: NimbleGen; ILL: Illumina; CR: conflicting results, indicating number of heterozygous variants with ≥20 reads in at least one sample, for which in other sample(s) this read depth was not reached; NA: not applicable (here: zero reads).

Table S18. Heterozygous SNVs identified by Sanger sequencing but not detected by at least one of the three WES platforms. SNVs are specified by gene, gDNA position, number of samples, enrichment platform (vendor) with false result, and read depth. In the right half of the table, true results at the same positions obtained in remaining data sets for the same sample(s) are presented.

Gene	DNA position	Within ROI	No. of samples	Platform	Design	Result (no. of results /no. of samples)	Change	NOR (sample)	Platform	Design	Result (no. of results /no. of samples)	NOR (sample)	Other info
COL3A1	Chr2:189857993T>C	no	2	ILL (V4)	flanking	false positive (2/2)	P--->PH	1 (2905), 2 (7344)	AG (V2)	flanking	true (2/2)	16 (2905), 28 (7344)	OK with V1; AG (V2 and V1): CR (2905)
									NG (V3)	flanking	true (2/2)	67 (2905), 56 (7344)	
	Chr2:189860630A>C	no	1	ILL (V4)	---	NA (1/1)	P--->NA	0 (2905)	AG (V2)	flanking	true (1/1)	20 (2905)	OK with V1; AG (V1) NOR<20
									NG (V3)	flanking	true(1/1)	28 (2905)	
	Chr2:189862352G>A	no	1	ILL (V4)	flanking	NA (1/1)	P--->NA	0 (2905)	AG (V2)	in design	true (1/1)	60 (2905)	OK with V1; ILL (V1) NOR<20
									NG (V3)	in design	true(1/1)	52 (2905)	
	Chr2:189862890G>A	no	1	ILL (V4)	---	NA (1/1)	P--->NA	0 (2905)	AG (V2)	flanking	true (1/1)	33 (2905)	OK with V1; ILL (V1) NOR<20
									NG (V3)	flanking	true(1/1)	33 (2905)	
	Chr2:189867882C>T	no	1	ILL (V4)	flanking	false positive (1/1)	P--->PH	1 (7344)	AG (V2)	flanking	true (1/1)	22 (7344)	OK with V1; AG (V1): NOR<20
									NG (V3)	---	true(1/1)	78 (7344)	
	Chr2:189871573A>G	no	1	ILL (V4)	flanking	false negative (1/1)	P--->WT	1 (2905)	AG (V2)	flanking	true (1/1)	16 (2905)	OK with V1; AG (V1) and ILL (V1) NOR<20
									NG (V3)	in design	true(1/1)	45 (2905)	
	Chr2:189875237T>G	no	1	ILL (V4)	---	NA (1/1)	P--->NA	0 (2905)	AG (V2)	---	true (1/1)	30 (2905)	OK with V1; AG (V1) NOR<20
									NG (V3)	flanking	true(1/1)	74 (2905)	
FBN1	Chr15:48903126A>G	no	4	ILL (V4)	---	NA (4/4)	P--->NA	0 (44), 0 (280), 0 (7344), 0 (7739)	AG (V2)	in design	true (4/4)	113 (44), 96 (280), 80 (7344), 78 (7739)	OK with V1; ILL NOR<20; NG (V3): CR
									NG (V3)	flanking	true (4/4)	13 (44), 50 (280), 42 (7344), 15 (7739)	
	Chr15:48813107G>T	no	2	ILL (V4)	flanking	NA (1/2)	P--->NA	0 (2905)	AG (V2)	flanking	true (2/2)	48 (2905), 31 (7344)	OK with V1; ILL (V1) NOR<20; NG (V1 and V3): CR
									NG (V3)	flanking	true (2/2)	14 (2905), 27 (7344)	
									ILL (V4)	flanking	true (1/2)	2 (7344)	
	Chr15:48797424C>A	no	2	ILL (V4)	flanking	false positive (1/2)	P--->PH	3 (7344)	AG (V2)	flanking	true (2/2)	22 (2905), 31 (7344)	OK with V1; AG (V1): CR; ILL (V1) NOR<20
									NG (V3)	flanking	true (2/2)	31 (2905), 49 (7344)	
									ILL (V4)	flanking	true (1/2)	7 (7344)	
	Chr15:48797124T>A	no	2	ILL (V4)	flanking	NA (2/2)	P--->NA	0 (44), 0 (2905)	AG (V2)	flanking	true (2/2)	51 (44), 35 (2905)	OK with V1; ILL (V1) NOR<20
									NG (V3)	flanking	true (2/2)	40 (44), 47 (2905)	
	Chr15:48795877G>A	no	2	ILL (V4)	---	NA (2/2)	P--->NA	0 (2905), 0 (7344)	AG (V2)	flanking	true (2/2)	9 (2905), 8 (7344)	
									NG (V3)	flanking	true (2/2)	20 (44), 34 (7344)	
				AG (V1)	flanking	NA (1/2)	P--->NA	0 (7344)	NG (V1)	flanking	true (2/2)	40 (2905), 41 (7344)	
									ILL (V1)	---	true (2/2)	15 (2905), 9 (7344)	
	Chr15:48784533G>A	no	1	ILL (V4)	---	NA (1/1)	P--->NA	0 (7739)	AG (V2)	---	true (1/1)	16 (7739)	OK with V1; all NOR<20
									NG (V3)	flanking	true (1/1)	6 (7739)	
	Chr15:48779138G>A	no	2	ILL (V4)	---	NA (2/2)	P--->NA	0 (44), 0 (7344)	AG (V2)	---	true (2/2)	10 (44), 3 (7344)	
									NG (V3)	---	true (2/2)	10 (44), 15 (7344)	
				NG (V1)	---	false negative (1/2)	P--->WT	4 (44)	AG (V1)	---	true (2/2)	6 (44), 3 (7344)	
									NG (V1)	---	true (1/2)	2 (7344)	
				ILL (V1)	---	false negative (1/2)	P--->WT	2 (44)	ILL (V1)	---	true (1/2)	2 (7344)	
	Chr15:48760391G>A	no	2	ILL (V4)*	flanking	false negative (1/2)	P--->WT	1 (2905)	AG (V2)	flanking	true (2/2)	34 (2905), 37 (7344)	OK with V1; ILL (V1) NOR<20
				ILL (V4)*	flanking	false positive (1/2)	P--->PH	2 (7344)	NG (V3)	flanking	true (2/2)	37 (2905), 46 (7344)	
	Chr15:48758132A>C	no	4	NG (V3)	flanking	false positive (1/4)	P--->PH	10 (7739)	AG (V2)	flanking	true (4/4)	43 (44), 45 (280), 50 (7344), 46 (7739)	OK with V1; ILL (V1): CR; NG (V3): also CR
									NG (V3)	flanking	true (3/4)	15 (44), 61 (280), 27 (7344)	
				ILL (V4)	flanking	false positive (1/4)	P--->PH	5 (7344)	ILL (V4)	flanking	true (3/4)	4 (44), 3 (280), 11 (7739)	
	Chr15:48736684A>T	no	3	ILL (V4)	flanking	false negative (1/3)	P--->WT	11 (280)	AG (V2)	in design	true (3/3)	137 (280), 120 (2905), 157 (7739)	
									NG (V3)	in design	true (3/3)	104 (280), 55 (2905), 31 (7739)	
									ILL (V4)	flanking	true (2/3)	7 (2905), 4 (7739)	
				ILL (V1)	flanking	false negative (1/3)	P--->WT	1 (7739)	AG (V1)	in design	true (3/3)	148 (280), 103 (2905), 133 (7739)	
									NG (V1)	in design	true (3/3)	102 (280), 100 (2905), 123 (7739)	
									ILL (V1)	flanking	true (2/3)	6 (280), 6 (2905)	
	Chr15:48736615T>C	no	2	ILL (V4)	---	NA (2/2)	P--->NA	0 (7344), 0 (7739)	AG (V2)	flanking	true (2/2)	51 (7344), 60 (7739)	OK with V1; ILL NOR<20
									NG (V3)	flanking	true (2/2)	30 (7344), 41 (7739)	
	Chr15:48729359A>T	no	2	ILL (V4)	flanking	false negative (1/2)	P--->WT	6 (2905)	AG (V2)	flanking	true (2/2)	61 (2905), 96 (7344)	OK with V1; ILL NOR<20
									NG (V3)	in design	true (2/2)	53 (2905), 75 (7344)	
									ILL (V4)	flanking	true (1/2)	5 (7344)	
	Chr15:48729030T>C	no	3	NG (V3)	flanking	false negative (1/3)	P--->WT	5 (7739)	AG (V2)	---	true (3/3)	54 (280), 31 (7344), 29 (7739)	
				ILL (V4)	---	NA (3/3)	P--->NA	0 (280), 0 (7344), 0 (7739)	NG (V3)	flanking	true (2/3)	43 (280), 30 (7344)	
									AG (V1)	---	true (3/3)	54 (280), 31 (7344), 29 (7739)	
				ILL (V1)	---	false positive (1/3)	P--->PH	1 (7739)	NG (V1)	flanking	true (2/3)	43 (280), 30 (7344)	
									ILL (V1)	---	true (2/3)	54 (280), 31 (7344), 29 (7739)	
	Chr15:48713996G>T	no	1	ILL (V4)	---	NA (1/1)	P--->NA	0 (44)	AG (V2)	flanking	true (1/1)	23 (44)	OK with V1; ILL NOR<20
									NG (V3)	flanking	true (1/1)	33 (44)	
SMAD3	Chr15:67358478G>A	UTR	1	NG (V1)	in design	NA (1/1)	P--->NA	0 (7739)	AG (V1)	flanking	true (1/1)	14 (7739)	AG (V2) and NG (V3) NOR<20, ILL (V4) NOR >20
									ILL (V1)	in design	true (1/1)	26 (7739)	
	Chr15:67457485G>C	no	1	NG (V1)	flanking	false positive (1/1)	P--->PH	1 (7739)	AG (V1)	flanking	true (1/1)	63 (7739)	AG (V2) and NG (V3) NOR<20, ILL (V4) NOR >20
									ILL (V1)	flanking	true (1/1)	11 (7739)	
	Chr15:67458930C>T	no	1	ILL (V4)	---	NA (1/1)	P--->NA	0 (7739)	AG (V2)	flanking	true (1/1)	117 (7739)	OK with V1; ILL NOR<20
									NG (V3)	---	true (1/1)	7 (7739)	
	Chr15:67459013C>T	no	1	ILL (V4)	---	NA (1/1)	P--->NA	0 (7739)	AG (V2)	flanking	true (1/1)	122 (7739)	OK with V1; all NOR>20
									NG (V3)	flanking	true (1/1)	15 (7739)	
TGFBR1	Chr9:101907279A>G	no	1	ILL (V4)	---	NA (1/1)	P--->NA	0 (44)	AG (V2)	---	true (1/1)	13 (44)	
									NG (V3)	flanking	true (1/1)	15 (44)	
				ILL (V1)	---	NA (1/1)	P--->NA	0 (44)	AG (V1)	---	true (1/1)	10 (44)	
									NG (V1)	flanking	true (1/1)	34 (44)	
	Chr9:101911321A>G	no	1	AG (V2)	---	false positive (1/1)	P--->PH	9 (44)	NG (V3)	---	true (1/1)	6 (44)	
				ILL (V4)	---	NA (1/1)	P--->NA	0 (44)	AG (V1)	---	true (1/1)	3 (44)	
				ILL (V1)	---	false positive (1/1)	P--->PH	1 (44)	NG (V1)	---	true (1/1)	2 (44)	

*In Supplementary Table S17 counted only once (i.e., one false result) due to same platform and vendor; NOR: Number of reads; AG: Agilent; NG: NimbleGen; ILL: Illumina; flanking: within 100 bp of design; OK, heterozygous position detected; CR: conflicting results, indicating number of heterozygous variants with ≥20 reads in at least one sample, for which in other sample(s) this read depth was not reached; design: designed target region; WT: wild-type; P: heterozygous SNV; PH: homozygous SNV; NA: not applicable (here: zero reads).

Table S19. False positive VCF calls in our ROI and UTR. Variants called heterozygous by WES, but wild-type in Sanger sequencing.

Sample	Gene	Chromosome	gDNA position	cDNA position	Location	WT allele	Mutant allele	Change	Platform	Vendor
44	*FBN1*	15	48'760'120	c.4747+15	intron 37	A (7)	T (2)	WT → P	Illumina	V4
7739	*FBN1*	15	48'718'056	c.7210	exon 58	C (16)	A (2)	WT → P	Illumina	V4
7739	*TGFBR2*	3	30'648'342	c.-34	5'UTR	G (5)	T (2)	WT → P	Illumina	V4

WT: wild-type; P: heterozygous SNV; calls in unfiltered VCF provided by vendors (cf. Supplementary Table S3).

Table S20. Heterozygous indels in our gene panel detected by Sanger sequencing in the six DNA samples of this study. The values are given as called by the Integrative Genomics Viewer (IGV): mean read depth across the variant (excluding reads carrying a deletion)/reads carrying the variant. Analysis was performed using unfiltered VCF files provided by vendors (cf. Supplementary Table S3).

Sample	Gene	Heterozygous indels detected by Sanger	Agilent_V1	NimbleGen_V1	Illumina_V1	Agilent_V2	NimbleGen_V3	Illumina_V4
44	*FBN1*	c.3209-72_67delTCTTTA	***106/21***[1]	***115/25***[1]	**16/5**[2]	***141/58***[1]	*58/0*[1]	**15/4**[1]
	FBN1	c.3589+36_40delTTTTA	**29/6**[2]	3/3[2]	**20/8**[2]	**46/10**[1]	23/0[2]	**16/4**[2]
	FBN1	c.3589+63_67delTTATG (homozygous)	17/5[2]	3/2[2]	5/1[2]	24/5[1]	14/0[2]	0/1[2]
	TGFBR1	c.1386+90_94delTCTTT	**26/9**[2]	**23/12**[2]	1/1[2]	**34/20**[1]	**16/15**[1]	NA[2]
280	*FBN1*	c.3209-72_67delTCTTTA	***106/26***[1]	***57/21***[1]	12/3[2]	***110/58***[1]	*99/0*[1]	20/2[1]
	FBN1	c.3589+63_67delTTATG	33/3[2]	2/0[2]	15/2[2]	23/20[1]	40/1[2]	4/1[2]
	FBN1	c.5924_5925dupAT (p.E1976MfsX5)*	***85/28***[1]	***86/29***[1]	***24/11***[1]	***93/55***[1]	***107/31***[1]	***20/5***[1]
326	*FBN1*	c.1904_1919del (p.Y635SfsX78)*	***37/7***[2]	***95/12***[2]	*37/3*[2]	***55/30***[1]	*60/0*[1]	***33/6***[1]
2905	*COL3A1*	c.2391+28delC	**13/14**[2]	***96/54***[1]	**24/15**[2]	**30/13**[1]	**58/32**[1]	**16/14**[1]
	FBN1	c.3589+36_40delTTTTA	**27/11**[2]	7/1[2]	**21/5**[2]	**26/11**[1]	33/0[2]	**28/5**[2]
	FBN1	c.3589+63_67delTTATG	18/3[2]	3/1[2]	9/1[2]	20/3[1]	19/0[2]	4/0[2]
	FBN1	c.5066-14dupT**	**119/42**[2]	***120/57***[1]	**19/5**[2]	**166/74**[1]	***67/19***[1]	**30/9**[2]
	FBN1	c.5423-30_28delCCT**	**25/12**[2]	***24/11***[1]	**59/26**[2]	**23/24**[1]	***29/17***[1]	**43/15**[1]
	FBN1	c.5671+28dupT	**67/24**[2]	***26/12***[1]	**21/11**[2]	**67/28**[1]	***34/20***[1]	**12/4**[2]
7344	*FBN1*	c.3209-72_67delTCTTTA	***123/28***[1]	***85/25***[1]	**13/6**[2]	***136/58***[1]	*69/0*[1]	**19/7**[1]
	FBN1	c.3589+36_40delTTTTA	**26/9**[2]	**7/7**[2]	17/0[2]	**30/17**[1]	41/0[2]	15/1[2]
	FBN1	c.3589+63_67delTTATG (homozygous)	15/2[2]	5/1[2]	6/3[2]	21/1[1]	22/0[2]	2/4[2]
	FBN1	c.5066-14dupT**	**150/60**[2]	***98/37***[1]	18/1[2]	**165/63**[1]	***93/25***[1]	**36/16**[2]
	FBN1	c.5423-30_28delCCT**	**33/21**[2]	***16/14***[1]	**46/25**[2]	**39/32**[1]	***21/31***[1]	**37/14**[1]
	FBN1	c.5671+28dupT	**88/35**[2]	***20/5***[2]	**14/6**[2]	**88/40**[1]	***38/17***[1]	**13/9**[2]
	TGFBR1	c.70_78del (p.A24_A26del)*	1/0[2]	*NA*[2]	<u>NA</u>[2]	5/0[2]	*4/0*[2]	<u>NA</u>[2]
7739	*FBN1*	c.3589+63_67delTTATG	14/1[2]	1/0[2]	7/0[2]	**9/4**[1]	9/0[2]	3/3[2]
Summary IGV total (unambiguous calls/total indels)			16/22	14/22	11/22	18/22	9/22	13/22
Summary VCF total (reported in VCF/total indels)			4/22***	10/22***	1/22***	21/22	13/22	8/22
**Summary IGV ROI (unambiguous calls/total indels)**			**6/7**	**6/7**	**4/7**	**6/7**	**5/7**	**6/7**
**Summary VCF ROI (reported in VCF/total indels)**			**1/7*** **	**5/7*** **	**1/7*** **	**6/7**	**6/7**	**4/7**

*exonic; **in our region of interest (ROI); ***note that in the VCF files of V1 only variants in designed target region are called (cf. Supplementary Table S3); NA: not applicable (here: zero reads); bold: unambiguous calls (>4 reads carrying indel reported by IGV); italic: in designed target region; unmarked: in region flanking to designed target region (within ±100 bp); underlined: outside the flanking region; [1]variant called in VCF; [2]variant not called in VCF.

Table S21. Genomic regions (182 exons) with copy numbers known from array CGH (highlighted in Supplementary Figure S26).

Chr	Start	End	Length [bp]	Exons	44	280	326*	2905	7344	7739
**1**	**206'315'856**	**206'407'396**	**91'541**	**9**	wt	wt	wt	het del	wt	wt
**4**	**69'373'934**	**69'491'021**	**117'088**	**6**	het del	het del	wt	wt	wt	het del
**8**	**15'950'680**	**16'023'855**	**73'176**	**7**	het del	wt	wt	wt	wt	wt
**8**	**39'232'101**	**39'387'546**	**155'446**	**30**	wt	het del	wt	het del	het del	hom del
**10**	**47'585'204**	**47'703'746**	**118'543**	**17**	dup	wt	wt	wt	wt	wt
**11**	**55'364'154**	**55'432'019**	**67'866**	**3**	wt	wt	wt	wt	het del	wt
**11**	**98'964'932**	**100'557'616**	**1'592'685**	**25**	wt	wt	wt	wt	het del	wt
**14**	**73'994'506**	**74'025'405**	**30'900**	**3**	wt	hom del	wt	het del	hom del	het del
**15**	**48'910'861**	**48'940'773**	**29'913**	**2**	het del	wt	wt	wt	wt	wt
**19**	**6'889'723**	**7'017'490**	**127'768**	**39**	wt	wt	wt	wt	wt	dup
**22**	**25'630'803**	**25'911'781**	**280'979**	**16**	het del	wt	wt	wt	wt	wt
**22**	**42'897'410**	**42'955'581**	**58'172**	**25**	wt	dup	wt	wt	wt	dup

* This sample was used as control for the relative base count quantification in WES data; wt, wild-type; het del, heterozygous deletion; hom del, homozygous deletion; dup, duplication.

Table S22. Total raw read counts reported by the four vendors (V1-V4).

**Sample**	**Agilent**		**NimbleGen**		**Illumina**	
	**V1**	**V2**	**V1**	**V3**	**V1**	**V4**
44	154'666'636	188'901'528	223'738'732	164'277'220	106'883'310	68'141'762
280	219'518'670	173'953'326	159'813'888	199'864'124	115'773'968	57'109'004
326	137'556'636	173'729'966	154'276'308	142'064'946	95'556'070	71'901'946
2905	130'559'938	144'762'290	178'017'756	149'900'120	133'200'476	72'876'912
7344	172'323'066	172'157'056	167'946'762	195'763'608	122'058'350	70'667'504
7739	151'440'766	178'561'090	190'473'376	99'625'288	89'940'532	62'495'098
**Mean**	**161'010'952**	**172'010'876**	**179'044'470**	**158'582'551**	**110'568'784**	**67'198'704**

Table S23. Proportion of duplicates reported by the four vendors (V1-V4).

Sample	Agilent		NimbleGen		Illumina	
	V1	V2	V1	V3	V1	V4
44	26.3%	8.0%	10.7%	50%*	12.3%	12.4%
280	43.9%	6.6%	12.5%	10%*	12.4%	11.1%
326	34.7%	8.2%	14.0%	50%*	11.6%	11.9%
2905	24.0%	9.5%	8.8%	30%*	13.2%	12.4%
7344	20.2%	8.2%	8.7%	30%*	12.2%	11.9%
7739	19.4%	10.0%	9.5%	50%*	11.7%	11.9%
**Mean**	**28.1%**	**8.4%**	**10.7%**	**37%***	**12.2%**	**11.9%**

*Number is estimated from provided graphs.

Table S24. Total mapped and deduplicated read counts (% of raw reads).

Sample	Agilent		NimbleGen		Illumina	
	V1	V2	V1	V3*	V1	V4*
44	110'584'430 (71.5%)	172'118'729 (91.1%)	186'538'676 (83.4%)	72'899'287 (44.4%)	88'290'788 (85.5%)	53’245'236 (78.1%)
280	119'682'978 (54.5%)	160'900'330 (92.5%)	129'934'094 (81.3%)	158'324'581 (79.2%)	95'545'396 (85.1%)	45’562'196 (79.8%)
326	87'403'593 (63.5%)	157'585'695 (90.7%)	122'855'749 (79.6%)	61'382'269 (43.2%)	79'962'090 (87.1%)	56’364'527 (78.4%)
2905	96'387'713 (73.8%)	129'503'393 (89.5%)	151'654'787 (85.2%)	90'817'153 (60.6%)	109'038'759 (84.8%)	57’138'610 (78.4%)
7344	133'734'129 (77.6%)	156'320'331 (90.8%)	143'047'403 (85.2%)	121'277'235 (62.0%)	101'342'654 (85.7%)	55’996'771 (79.2%)
7739	118'295'260 (78.1%)	156'695'689 (87.8%)	159'150'546 (83.6%)	41'985'030 (42.1%)	72'695'510 (87.0%)	47’498'550 (76.0%)
**Mean**	**111'014'684 (69.9%)**	**155'520'695 (90.4%)**	**148'863'543 (83.0%)**	**91'114'259 (55.2%)**	**91'145'866 (85.7%)**	**52â634'315 (78.3%)**

*Only unique reads (cf. Supplementary Table S3).

Table S25. Enrichment and detection of non-reference (alternative) alleles in VCF files provided by vendors (V1-V4). Fraction (%) of non-reference (alternative) alleles for shared sequence variants targeted by each platform and located within RefSeq coding exons completely (100%) covered with ≥20 reads by all six platform-vendor combinations. Analysis was performed using filtered and recalibrated (V1), filtered only (V2) or unfiltered VCF files (V3 andV4) provided by vendor V1 using the same data analysis workflow for all three platforms ensuring best comparability and vendors V2-V4 with different data analysis settings as specified in Supplementary Table S3.

**SNVs and indels**	**44**	**280**	**326**	**2905**	**7344**	**7739**	**Total**
Number of variants	2'631	1'856	2'079	2'837	2'915	1'421	**13'739**
Number of heterozygous variants	1'707	1'196	1'294	1'812	1'772	957	**8'738**
Agilent_V1 alternative allele [%]	47.98±0.31	47.89±0.33	48.08±0.38	47.96±0.32	48.10±0.28	47.97±0.38	**47.40±0.14**
Agilent_V2 alternative allele [%]*	48.41±0.25	48.28±0.31	48.23±0.29	48.34±0.29	48.36±0.26	48.18±0.35	**47.60±0.12**
NimbleGen_V1 alternative allele [%]	46.12±0.32	46.31±0.40	45.90±0.41	46.55±0.31	46.66±0.33	46.57±0.42	**47.64±0.15**
NimbleGen_V3 alternative allele [%]*	48.07±0.39	48.01±0.33	48.11±0.47	47.90±0.34	48.01±0.30	47.96±0.65	**47.77±0.16**
Illumina_V1 alternative allele [%]	47.90±0.37	47.66±0.46	48.59±0.45	47.92±0.35	48.00±0.36	48.54±0.54	**46.86±0.17**
Illumina_V4 alternative allele [%]*	46.28±0.44	45.56±0.60	45.86±0.51	45.99±0.42	46.32±0.41	45.80±0.58	**46.32±0.19**
**SNVs**	**44**	**280**	**326**	**2905**	**7344**	**7739**	**Total**
Number of variants	2'606	1'836	2'058	2'801	2'876	1'402	**13'579**
Number of heterozygous variants	1'697	1'188	1'289	1'801	1'761	951	**8'687**
Agilent_V1 alternative allele [%]	47.99±0.31	47.92±0.33	48.10±0.38	47.97±0.32	48.11±0.28	48.00±0.38	**47.42±0.14**
Agilent_V2 alternative allele [%]*	48.43±0.25	48.30±0.31	48.27±0.28	48.35±0.29	48.38±0.26	48.18±0.35	**47.62±0.12**
NimbleGen_V1 alternative allele [%]	46.16±0.32	46.38±0.40	45.96±0.41	46.56±0.31	46.73±0.33	46.56±0.42	**47.68±0.15**
NimbleGen_V3 alternative allele [%]*	48.07±0.39	48.02±0.33	48.12±0.47	47.91±0.34	48.02±0.30	47.97±0.66	**47.78±0.16**
Illumina_V1 alternative allele [%]	47.93±0.37	47.73±0.47	48.65±0.45	47.95±0.35	48.02±0.36	48.56±0.54	**46.94±0.17**
Illumina_V4 alternative allele [%]*	46.29±0.44	45.60±0.60	45.88±0.51	45.99±0.42	46.34±0.41	45.77±0.58	**46.37±0.20**
**Indels**	**44**	**280**	**326**	**2905**	**7344**	**7739**	**Total**
Number of variants	25	20	21	36	39	19	**160**
Number of heterozygous variants	10	8	5	11	11	6	**51**
Agilent_V1 alternative allele [%]	47.13±4.66	42.99±4.92	41.25±8.58	46.95±4.68	47.05±4.06	45.45±6.26	**47.11±1.81**
Agilent_V2 alternative allele [%]*	44.85±3.83	43.95±4.55	36.27±6.48	46.62±4.19	45.60±3.78	46.99±5.83	**47.20±1.58**
NimbleGen_V1 alternative allele [%]	40.39±4.77	36.63±5.88	32.78±9.27	44.21±4.53	35.69±4.69	48.87±6.99	**47.00±1.95**
NimbleGen_V3 alternative allele [%]*	48.10±5.85	47.70±4.83	44.34±10.69	45.20±5.02	47.19±4.34	45.69±10.82	**47.55±2.19**
Illumina_V1 alternative allele [%]	43.13±5.51	38.04±6.85	34.65±10.14	43.15±5.10	43.57±5.17	44.39±8.86	**45.55±2.22**
Illumina_V4 alternative allele [%]*	45.10±6.64	39.41±8.81	42.18±11.51	44.31±6.05	43.94±5.86	50.17±9.63	**45.42±2.61**

Indicated ranges for mean values (±) represent 95% confidence intervals; *since each vendor applied a different data analysis workflow (cf. Supplementary Table S3), the restriction to shared sequence variants targeted by the design of each platform and located within RefSeq coding exons completely covered at 20× by all six platform-vendor combinations should largely exclude possible false-positive allele calls.

Table S26. Enrichment and detection of non-reference (alternative) alleles in gVCF files generated by the same in-house bioinformatics pipeline. Fraction (%) of alternative alleles for shared sequence variants within the platforms' designed target region and 50-bp flanking sequences achieving ≥20 reads and >30 quality scores by all six platform-vendor combinations.

**SNVs and indels**	**44**	**280**	**326**	**2905**	**7344**	**7739**	**Total**
Number of variants	27'818	21'518	23'758	28'188	28'347	21'565	**151'194**
Number of heterozygous variants	18'152	13'971	15'142	17'876	18'163	14'499	**97'803**
Agilent_V1 alternative allele [%]	47.33±0.12	47.61±0.13	47.45±0.14	47.41±0.12	47.40±0.11	47.22±0.13	**47.40±0.05**
Agilent_V2 alternative allele [%]	47.58±0.11	47.69±0.12	47.64±0.12	47.58±0.12	47.56±0.11	47.55±0.13	**47.60±0.05**
NimbleGen_V1 alternative allele [%]	47.83±0.12	47.70±0.14	47.50±0.14	47.69±0.13	47.55±0.13	47.56±0.14	**47.64±0.05**
NimbleGen_V3 alternative allele [%]	47.88±0.13	47.38±0.12	47.94±0.15	47.73±0.12	47.76±0.11	47.88±0.18	**47.77±0.06**
Illumina_V1 alternative allele [%]	46.86±0.15	46.93±0.16	46.56±0.17	46.97±0.14	47.12±0.14	46.64±0.18	**46.86±0.06**
Illumina_V4 alternative allele [%]	46.53±0.16	46.10±0.19	46.04±0.18	46.40±0.16	46.50±0.16	46.21±0.18	**46.32±0.07**
**SNVs**	**44**	**280**	**326**	**2905**	**7344**	**7739**	**Total**
Number of variants	26'221	20'285	22'422	26'470	26'654	20'312	**142'364**
Number of heterozygous variants	17'138	13'163	14'289	16'793	17'082	13'693	**92'158**
Agilent_V1 alternative allele [%]	47.33±0.12	47.63±0.14	47.46±0.14	47.43±0.13	47.42±0.12	47.26±0.14	**47.42±0.05**
Agilent_V2 alternative allele [%]	47.60±0.11	47.72±0.13	47.65±0.12	47.62±0.12	47.59±0.11	47.58±0.13	**47.62±0.05**
NimbleGen_V1 alternative allele [%]	47.86±0.13	47.73±0.15	47.51±0.14	47.74±0.13	47.60±0.13	47.63±0.14	**47.68±0.06**
NimbleGen_V3 alternative allele [%]	47.91±0.14	47.40±0.12	47.96±0.16	47.74±0.13	47.78±0.11	47.86±0.18	**47.78±0.06**
Illumina_V1 alternative allele [%]	46.92±0.15	47.01±0.17	46.66±0.17	47.05±0.15	47.18±0.15	46.72±0.18	**46.94±0.07**
Illumina_V4 alternative allele [%]	46.58±0.16	46.17±0.20	46.08±0.18	46.47±0.16	46.53±0.16	46.28±0.19	**46.37±0.07**
**Indels**	**44**	**280**	**326**	**2905**	**7344**	**7739**	**Total**
Number of variants	1'597	1'233	1'336	1'718	1'693	1'253	**8'830**
Number of heterozygous variants	1'014	808	853	1'083	1'081	806	**5'645**
Agilent_V1 alternative allele [%]	47.41±0.55	47.28±0.57	47.23±0.61	47.11±0.53	47.04±0.47	46.53±0.62	**47.11±0.23**
Agilent_V2 alternative allele [%]	47.34±0.50	47.30±0.54	47.39±0.53	47.07±0.50	47.09±0.45	47.07±0.59	**47.20±0.21**
NimbleGen_V1 alternative allele [%]	47.86±0.55	47.73±0.60	47.51±0.59	47.74±0.51	47.60±0.51	47.63±0.62	**47.00±0.23**
NimbleGen_V3 alternative allele [%]	47.44±0.58	47.11±0.51	47.57±0.65	47.55±0.51	47.41±0.47	48.31±0.75	**47.55±0.23**
Illumina_V1 alternative allele [%]	45.71±0.69	45.62±0.76	44.86±0.78	45.66±0.62	46.04±0.60	45.21±0.82	**45.55±0.29**
Illumina_V4 alternative allele [%]	45.73±0.71	44.85±0.87	45.37±0.81	45.25±0.70	46.06±0.66	45.06±0.85	**45.42±0.31**

Variant positions with more than one different non-reference allele (non-biallelic) as well as variant calls with alternative allele percentages outside 10-90% were excluded; indicated ranges for the mean values (±) represent 95% confidence intervals.

Table S27. Array CGH data for three DNA samples (44, 7344, and 7739). Common coding SNVs of NimbleGen CGH/LOH array within designed target region and exons completely covered at ≥20 reads by WES performed by all platforms and vendors compared to WES variant calls (unfiltered VCF files provided by vendors, cf. Supplementary Table S3).

	**44**	**7344**	**7739**
Total shared covered coding exons	65'135	68'992	39'704
Total SNVs from array CGH in covered exons	93	101	53
Heterozygous SNPs	44*	29*	20*
Homozygous SNPs	10*	23*	6*
Wild-type SNPs	27**	41**	13**
No/false array results	12	8	14

*Correctly called regardless of WES platform and vendor; **correctly recognised as wild-type regardless of WES platform and vendor.

Table S28. Assessment of influence of laboratory workflow on variant calling for the three enrichment platforms by comparing gVCF files generated by the same in-house bioinformatics pipeline using FASTQ files provided by vendors V1-V4 for all three enrichment platforms (six data sets per DNA sample) and calling genotypes for genomic positions within the platforms' designed target regions and 50-bp flanking sequences which achieved ≥20 reads and >30 quality scores (cf. Supplementary Table S3). Positions with a heterozygous genotype call by at least one of the three platforms (variant positions) in common covered regions of all six platform-vendor combinations were analysed revealing differences in variant detection among the three platforms influenced by laboratory workflow (cf. results of V1 vs. results of V2-V4).

Heterozygous sequence variant positions	44	280	326	2905	7344	7739	Mean
Total sequence variant positions	19'051 (100%)	14'763 (100%)	15'944 (100%)	18'752 (100%)	19'068 (100%)	15'240 (100%)	**17'136 (100%)**
Heterozygous in all six platform-vendor combinations*	18'152 (95.28%)	13'971 (94.64%)	15'142 (94.97%)	17'876 (95.33%)	18'163 (95.25%)	14'499 (95.14%)	**16'301 (95.1±0.27%)**
No non-reference allele in Agilent V1 and V2 only**	24 (0.13%)	28 (0.19%)	23 (0.14%)	36 (0.19%)	20 (0.10%)	22 (0.14%)	**26 (0.15±0.04%)**
No non-reference allele in Agilent V1 only**	10 (0.05%)	9 (0.06%)	23 (0.14%)	10 (0.05%)	24 (0.13%)	23 (0.15%)	**17 (0.10±0.05%)**
No non-reference allele in Agilent V2 only**	7 (0.04%)	6 (0.04%)	4 (0.03%)	4 (0.02%)	3 (0.02%)	13 (0.09%)	**6 (0.04±0.03%)**
No non-reference allele in NimbleGen V1 and V3 only**	11 (0.06%)	5 (0.03%)	9 (0.06%)	6 (0.03%)	5 (0.03%)	4 (0.03%)	**7 (0.04±0.02%)**
No non-reference allele in NimbleGen V1 only**	18 (0.09%)	24 (0.16%)	13 (0.08%)	16 (0.09%)	23 (0.12%)	18 (0.12%)	**19 (0.11±0.03%)**
No non-reference allele in NimbleGen V3 only**	16 (0.08%)	16 (0.11%)	7 (0.04%)	16 (0.09%)	9 (0.05%)	14 (0.09%)	**13 (0.08±0.03%)**
No non-reference allele in Illumina V1 and V4 only**	90 (0.47%)	79 (0.54%)	81 (0.51%)	93 (0.50%)	79 (0.41%)	77 (0.51%)	**83 (0.49±0.04%)**
No non-reference allele in Illumina V1 only**	37 (0.19%)	21 (0.14%)	31 (0.19%)	19 (0.10%)	31 (0.16%)	42 (0.28%)	**30 (0.18±0.06%)**
No non-reference allele in Illumina V4 only**	49 (0.26%)	68 (0.46%)	48 (0.30%)	51 (0.27%)	49 (0.26%)	42 (0.28%)	**51 (0.30±0.08%)**
Heterozygous in Agilent V1 and V2 only***	21 (0.11%)	20 (0.14%)	20 (0.13%)	24 (0.13%)	34 (0.18%)	21 (0.14%)	**23 (0.14±0.02%)**
Heterozygous in Agilent V1 only***	24 (0.13%)	27 (0.18%)	16 (0.10%)	28 (0.15%)	21 (0.11%)	23 (0.15%)	**23 (0.14±0.03%)**
Heterozygous in Agilent V2 only***	59 (0.31%)	39 (0.26%)	47 (0.29%)	48 (0.26%)	60 (0.31%)	23 (0.15%)	**46 (0.27±0.06%)**
Heterozygous in NimbleGen V1 and V3 only***	45 (0.24%)	16 (0.11%)	38 (0.24%)	17 (0.09%)	32 (0.17%)	20 (0.13%)	**28 (0.16±0.07%)**
Heterozygous in NimbleGen V1 only***	77 (0.40%)	75 (0.51%)	65 (0.41%)	57 (0.30%)	66 (0.35%)	64 (0.42%)	**67 (0.40±0.07%)**
Heterozygous in NimbleGen V3 only***	142 (0.75%)	96 (0.65%)	111 (0.70%)	140 (0.75%)	155 (0.81%)	112 (0.73%)	**126 (0.73±0.06%)**
Heterozygous in Illumina V1 and V4 only***	21 (0.11%)	31 (0.21%)	39 (0.24%)	29 (0.15%)	23 (0.12%)	19 (0.12%)	**27 (0.16±0.06%)**
Heterozygous in Illumina V1 only***	54 (0.28%)	43 (0.29%)	53 (0.33%)	48 (0.26%)	54 (0.28%)	36 (0.24%)	**48 (0.28±0.03%)**
Heterozygous in Illumina V4 only***	51 (0.27%)	46 (0.31%)	32 (0.20%)	43 (0.23%)	49 (0.26%)	34 (0.22%)	**43 (0.25±0.04%)**
Further observed genotypes****	143 (0.75%)	143 (0.97%)	142 (0.89%)	191 (1.02%)	168 (0.88%)	134 (0.88%)	**154 (0.90±0.10%)**

Variant positions with more than one different alternative allele (non-biallelic) or heterozygous calls at non-reference allele fractions outside the range of 10-90% were excluded from analysis; values in parentheses indicate fraction of total analysed variant positions and ranges for the mean values (±) represent 95% confidence intervals; *to be considered as true positive calls (note that alignment errors cannot be excluded); **may harbour false negative calls (remaining platform-vendor combinations have heterozygous calls); ***may harbour false positive calls (remaining platform-vendor combinations called no non-reference allele); ****rare patterns of different calls among platform-vendor combinations not listed in this table, including also potentially homozygous positions.

Table S29. Intervals within which 95% of the relative base counts of 21'769 RefSeq exons (covered at 20× in all 36 platform-vendor-sample combinations) used for normalisation in Supplementary Figure S24 lie (calculated according to the Student's *t* distribution as the mean of *n* values ± critical *t* value ($t_{crit,n-1}$) × *SD* using *n* = 21'769 and 108'845 for individual and the total of five samples, respectively, t_{crit} = 1.960). The number of dots with relative base counts >3 and thus not shown in Supplementary Figure S24 is given in parentheses.

	44	280	326*	2905	7344	7739	Total**
**Agilent_V1**	1.01±0.34 (1)	1.01±0.32 (0)	1.00±0.00 (0)	1.01±0.34 (1)	1.01±0.32 (0)	1.01±0.33 (0)	**1.01±0.33 (2)**
**Agilent_V2**	1.00±0.28 (1)	1.01±0.28 (0)	1.00±0.00 (0)	1.01±0.29 (1)	1.01±0.28 (0)	1.01±0.28 (0)	**1.01±0.28 (2)**
**NimbleGen_V1**	1.00±0.37 (2)	1.00±0.41 (2)	1.00±0.00 (0)	1.00±0.37 (2)	1.00±0.38 (2)	1.00±0.43 (2)	**1.00±0.39 (10)**
**NimbleGen_V3**	1.02±0.41 (1)	0.99±0.46 (2)	1.00±0.00 (0)	1.01±0.41 (1)	1.01±0.38 (0)	1.03±0.43 (0)	**1.01±0.42 (4)**
**Illumina_V1**	1.00±0.41 (1)	1.00±0.44 (0)	1.00±0.00 (0)	1.00±0.43 (1)	1.00±0.46 (0)	1.01±0.38 (0)	**1.00±0.42 (2)**
**Illumina_V4**	1.02±0.42 (2)	1.04±0.46 (0)	1.00±0.00 (0)	1.03±0.42 (2)	1.02±0.42 (0)	1.03±0.43 (0)	**1.03±0.43 (4)**

* Base counts are calculated relative to this sample (i.e. 1.00±0.00 (0) is expected/trivial), **excluding sample 326.

Table S30. Intervals within which 95% of the relative base counts of wild-type RefSeq exons (two copies, displayed as black dots in Supplementary Figure S26) lie (calculated according to the Student's *t* distribution as the mean of *n* values ± critical *t* value ($t_{crit,n-1}$) × *SD* using the *n* values indicated in the table and the corresponding t_{crit} values). The number of dots with relative base counts >3 (out of a total of ~800'000 dots with relative base counts derived from ~160'000 RefSeq exons of five DNA samples, "grey dots") and thus not shown in Supplementary Figure S26 is given in parentheses.

	44	280	326*	2905	7344	7739	Total**
**Agilent_V1**	1.01±0.38 (24)<br>*n* = 112	1.00±0.31 (19)<br>*n* = 111	1.00±0.00 (0)<br>*n* = 154	1.00±0.34 (18)<br>*n* = 132	0.98±0.39 (12)<br>*n* = 114	0.99±0.27 (11)<br>*n* = 72	**1.00±0.34 (84)<br>*n* = 541**
**Agilent_V2**	0.99±0.30 (24)<br>*n* = 114	0.98±0.27 (29)<br>*n* = 112	1.00±0.00 (0)<br>*n* = 157	1.02±0.32 (21)<br>*n* = 134	0.96±0.37 (13)<br>*n* = 116	1.00±0.23 (15)<br>*n* = 73	**0.99±0.31 (102)<br>*n* = 549**
**NimbleGen_V1**	1.02±0.45 (92)<br>*n* = 121	0.94±0.51 (52)<br>*n* = 111	1.00±0.00 (0)<br>*n* = 162	1.04±0.44 (51)<br>*n* = 124	1.12±0.63 (140)<br>*n* = 105	1.12±0.69 (84)<br>*n* = 72	**1.04±0.54 (419)<br>*n* = 533**
**NimbleGen_V3**	0.98±0.41 (21)<br>*n* = 118	0.96±0.43 (476)<br>*n* = 113	1.00±0.00 (0)<br>*n* = 161	0.97±0.39 (24)<br>*n* = 124	0.96±0.39 (34)<br>*n* = 105	0.98±0.36 (16)<br>*n* = 74	**0.97±0.39 (571)<br>*n* = 534**
**Illumina_V1**	1.03±0.41 (49)<br>*n* = 129	1.05±0.45 (53)<br>*n* = 113	1.00±0.00 (0)<br>*n* = 173	1.11±0.41 (41)<br>*n* = 134	1.12±0.46 (45)<br>*n* = 115	1.03±0.45 (20)<br>*n* = 75	**1.07±0.44 (208)<br>*n* = 566**
**Illumina_V4**	1.00±0.50 (30)<br>*n* = 117	1.02±0.49 (29)<br>*n* = 111	1.00±0.00 (0)<br>*n* = 154	1.01±0.44 (28)<br>*n* = 119	0.97±0.37 (32)<br>*n* = 100	1.02±0.40 (26)<br>*n* = 73	**1.00±0.44 (145)<br>*n* = 520**

* Base counts are calculated relative to this sample (i.e. 1.00±0.00 (0) is expected/trivial), **excluding sample 326.
